# Unveiling MYCN regulatory networks in neuroblastoma via integrative analysis of heterogeneous genomics data

**DOI:** 10.18632/oncotarget.9202

**Published:** 2016-05-06

**Authors:** Chia-Lang Hsu, Hsin-Yi Chang, Jen-Yun Chang, Wen-Ming Hsu, Hsuan-Cheng Huang, Hsueh-Fen Juan

**Affiliations:** ^1^ Department of Life Science, Institute of Molecular and Cellular Biology, Graduate Institute of Biomedical Electronics and Bioinformatics, National Taiwan University, Taipei 106, Taiwan; ^2^ Department of Surgery, National Taiwan University Hospital, Taipei 100, Taiwan; ^3^ Institute of Biomedical Informatics, Center for Systems and Synthetic Biology, National Yang-Ming University, Taipei 112, Taiwan

**Keywords:** genomics, neuroblastoma, MYCN, regulatory network, microRNA

## Abstract

MYCN, an oncogenic transcription factor of the Myc family, is a major driver of neuroblastoma tumorigenesis. Due to the difficulty in drugging MYCN directly, revealing the molecules in MYCN regulatory networks will help to identify effective therapeutic targets for neuroblastoma therapy. Here we perform ChIP-sequencing and small RNA-sequencing of neuroblastoma cells to determine the MYCN-binding sites and MYCN-associated microRNAs, and integrate various types of genomic data to construct MYCN regulatory networks. The overall analysis indicated that MYCN-regulated genes were involved in a wide range of biological processes and could be used as signatures to identify poor-prognosis MYCN-non-amplified patients. Analysis of the MYCN binding sites showed that MYCN principally served as an activator. Using a computational approach, we identified 32 MYCN co-regulators, and some of these findings are supported by previous studies. Moreover, we investigated the interplay between MYCN transcriptional and microRNA post-transcriptional regulations and identified several microRNAs, such as miR-124-3p and miR-93-5p, which may significantly contribute to neuroblastoma pathogenesis. We also found MYCN and its regulated microRNAs acted together to repress the tumor suppressor genes. This work provides a comprehensive view of MYCN regulations for exploring therapeutic targets in neuroblastoma, as well as insights into the mechanism of neuroblastoma tumorigenesis.

## INTRODUCTION

Neuroblastoma (NB) is one of the most common extracranial solid tumors in infancy. These tumors occur most frequently in the adrenal medulla, but can originate anywhere along the sympathetic nervous system [1]. NB cells exhibit similar characteristics to undifferentiated cells and often metastasize to distant organs [2]. Approximately 60% of patients diagnosed with NB display a late disease stage and have very poor prognosis. Patients with high-risk NB have a five-year survival rate of less than 50%, even with aggressive therapy [3]. Several genetic alterations are commonly found in NB cells, including MYCN amplification, 1p deletion, 11q deletion, and 17q gain, and these are often associated with high-risk tumors and an unfavorable outcome [4–7]. Understanding the molecular mechanisms underlying these genetic alterations might therefore be helpful for the development of NB risk assessment and therapy.

MYCN is one of the best-known prognostic markers of NB. MYCN amplification is detected in approximately 25% of NB tumors [8]. Patients with NB tumors containing a single copy of MYCN usually have a favorable prognosis, whereas amplification and/or MYCN overexpression result in rapid disease progression and a high mortality rate [6]. MYCN belongs to the Myc family of proto-oncogenes, which have a conserved structure, including a transcriptional activation domain at the N-terminus and a basic-helix-loop-helix-zipper (bHLHZ) domain at the C-terminus. MYCN is primarily known to act as an activator by heterodimerizing with MAX to bind specific E-box DNA motifs (CANNTC). Recently, however, MYCN has also been shown to have the ability to repress the transcription of target genes through the recruitment of corepressors [9]. For example, through interaction with SP1 and MIZ1 at promoters, MYCN silences gene expression via recruitment of the histone deacetylase HDAC1 [10]. The target genes of MYCN are involved in diverse cellular functions in malignancy, including cell cycle, apoptosis, proliferation, pluripotency, differentiation, angiogenesis and immune surveillance [11].

In addition to protein-coding genes, MYCN has also been shown to bind to the promoter region of a wide range of microRNAs for regulation of their expression in NB. MicroRNAs (miRNAs) are short non-coding RNAs of 20–24 nucleotides that play important roles in many biological pathways via post-transcriptional regulation of their target mRNAs. Many studies have reported that the dysregulation of some miRNAs is associated with the pathobiology of many cancer types, including NB [12–15]. Several oncogenic miRNAs, such as the miR-17-92 cluster, are directly activated by MYCN to promote cell proliferation and inhibit apoptosis [13]. MYCN also inhibits several tumor suppressor miRNAs, such as miR-184 [12]. These findings indicate that MYCN can exert both transcriptional and post-transcriptional regulation on its targets.

It is thus clear that MYCN is the most important NB therapeutic target. However, because of the pleiotropic effects of MYCN and the difficulty in drugging transcription factors, it has been challenging to design therapeutic strategies that directly target MYCN [16]. An alternative approach is to develop drugs that inactivate MYCN partners or transcriptional targets [17]. To this end, integration of various regulatory interactions and the construction of comprehensive MYCN regulatory networks in NB are required. A few studies have used integrative omics approaches to identify the critical regulators or effector of MYCN in NB and potential therapeutic targets [18, 19]. In this study, we performed chromatin immunoprecipitation following by sequencing (ChIP-seq) and small RNA sequencing to identify MYCN binding sites and MYCN-associated miRNAs, and then used an integrative approach to dissect the MYCN regulatory networks.

## RESULTS AND DISCUSSION

### MYCN-regulated genes involved in diverse roles in neuroblastoma

To identify MYCN binding sites across the genome, we performed ChIP-seq using anti-MYCN and anti-IgG antibodies in a MYCN-amplified NB cell line, SK-N-BE(2)-C. After read alignment and peak calling, a total of 72,737 regions were significantly enriched. To obtain high-confidence MYCN binding sites, the enriched regions had to be overlapped with the binding sites of other transcription factors or regulators derived from the ENCODE project. Finally, 22,526 MYCN binding regions (positive peaks) were identified.

**Figure 1:**
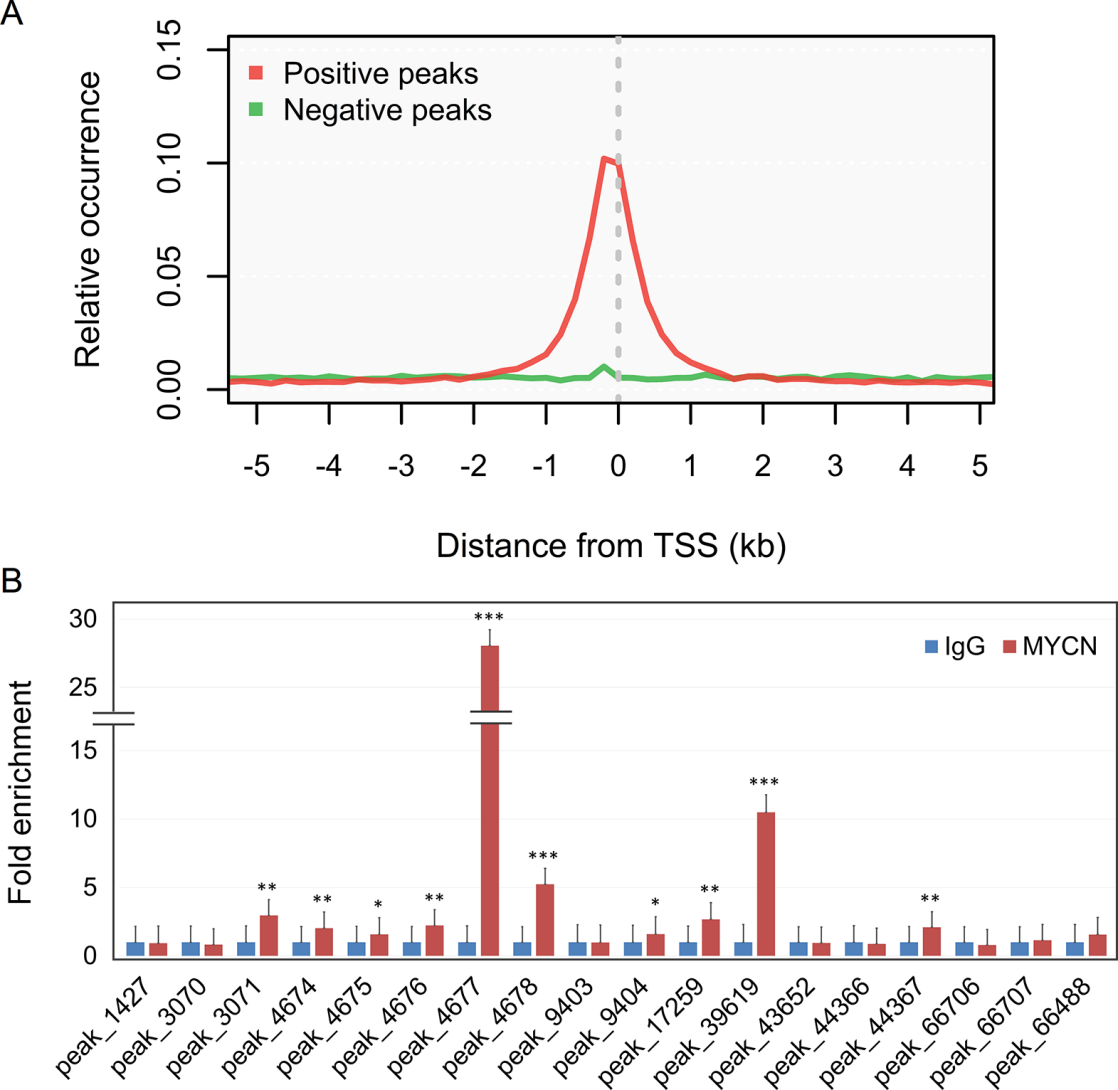
Distribution and validation of MYCN binding sites (**A**) Relative occurrence of MYCN binding peaks per 200-bp bin corresponding to the 5-kb region flanking all annotated TSSs. Positive and negative peaks denote the binding regions with and without other regulatory bindings, respectively, based on the ChIP-seq experiments of the ENCODE project. (**B**) ChIP-qPCR validation of MYCN binding to genomic regions associated with the promoters of miRNAs or miRNA-hosted genes identified in the ChIP-seq experiment. Ten out of 18 selected sites could be validated by a single gene. Error bars represent SD; *n* = 3; two-tailed Student *t*-test: *p < 0.05, **p < 0.01, ***p < 0.001.

We compared the MYCN binding regions to the other studies (Supplementary Table S1) and found that 40% of MYCN binding regions identified in the other cell lines were overlapped by that we identified (Supplementary Figure S1). In addition, several known MYCN-regulated genes (NME2 [20], CRABP2 [21], LIF [22], MDM2 [23], MIR17HG [24], PRMT1 [25], MCM7 [26], MCM8 [26], ODC1 [27], BIRC5 [28], LUC7L [29], TWIST1 [30], RAB5C [31], AURKA [31], H1F0 [31], and MYBL2 [32]) were successfully detected in the ChIP-seq experiments (Supplementary Figure S2). To study the distribution of MYCN binding around promoter sequences, we aligned the peaks with the annotated transcriptional start sites (TSSs), which were provided by RefSeq. Most of the MYCN binding sites were concentrated around TSSs, within −1 kb to +1 kb (Figure 1A), consistent with previous studies on MYCN [29, 31, 33]. Additionally, some of the MYCN binding sites were verified using ChIP-qPCR (Figure 1B). Together, these results confirm the validity of our ChIP-seq experiments.

**Figure 2:**
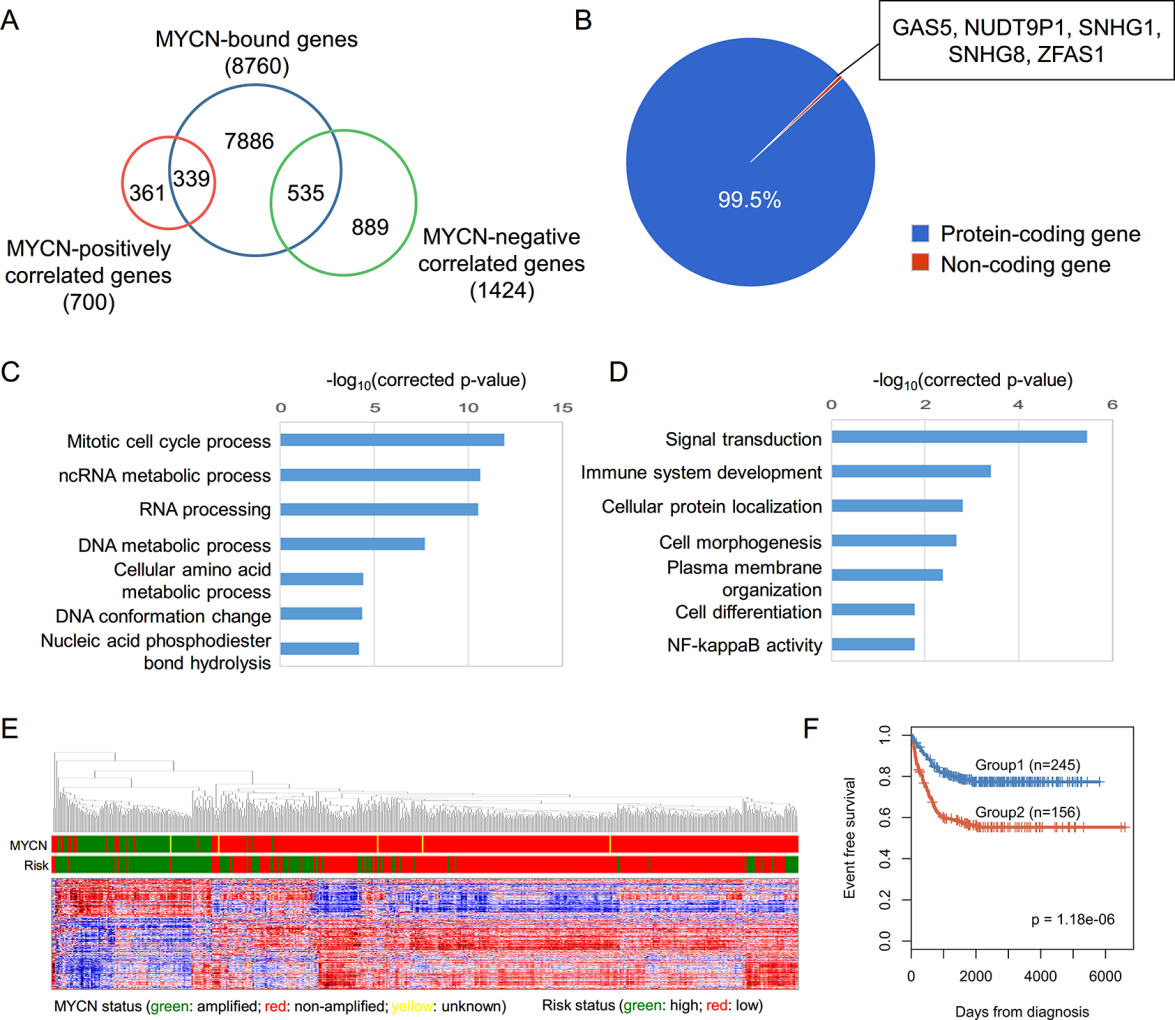
Systematic analysis of MYCN-regulated genes (**A**) Identification of MYCN-regulated genes based on the overlap of MYCN-bound genes and MYCN-correlated genes. (**B**) Pie chart depicting the percentage of different classes of MYCN-regulated genes. (C, D) GO analysis of MYCN-activated (**C**) and MYCN-repressed (**D**) genes. (**E**) A hierarchical clustering graph representing the association between the expression of MYCN-regulated genes and MYCN, as well as risk status. Data from SEQC RNA-seq are represented as a Pearson correlation metric with average linkage. (**F**) Kaplan-Meier survival curves of MYCN-non-amplified NB patient groups defined by *k*-means clustering of expression of 874 MYCN-regulated genes. The graph depicts the *p*-value as obtained from the Log-rank test. Numbers in parentheses are the number of patients in each group.

Since the exact promoter region for each gene was unclear, we used a broad window to determine the MYCN-bound genes. According to the known MYCN-regulated genes (Supplementary Figure S2), if an MYCN binding site fell within −10 kb or +2 kb of a TSS, it was defined as an MYCN-bound gene. A total of 8,760 MYCN-bound genes were identified. To clarify the regulation of the MYCN-bound genes, we used the NB gene expression data to infer gene regulation of MYCN. We calculated the Spearman correlation coefficient between MYCN and other genes, and selected the genes that were strongly positively or negatively correlated with MYCN, i.e. |Spearman correlation coefficient| ≥ 0.3 for both profiling methods. A total of 700 MYCN-positively correlated genes and 1424 MYCN-negatively correlated genes were identified (Supplementary Table S2). Merging the MYCN-bound genes and the MYCN-correlated genes, we obtained 874 direct transcriptional MYCN targets, hereafter termed MYCN-regulated genes (Figure 2A). Based on the direction of the regulation, these genes were classified into 339 MYCN-activated genes and 535 MYCN-repressed genes (Supplementary Table S3). Some of these MYCN-regulated genes are also detected by the other studies (Supplementary Figure S3).

The majority of the MYCN-regulated genes were protein-coding genes, but there were five non-coding genes: NUDT9P1, GAS5, SNHG1, SNHG8, and ZFAS1 (Figure 2B). Interestingly, expression of NUDT9P1 and SNHG1 was associated with the prognosis of MYCN-non-amplified NB patients (Supplementary Figure S4). Additionally, GAS5 and ZFAS1 have been identified as oncogenes or tumor suppressor genes in other cancer types [34, 35]. These MYCN-driven non-coding genes might also play critical roles in NB carcinogenesis.

To investigate the principal pathways in which the MYCN-regulated genes are involved, we performed a GO enrichment analysis using a Cytoscape plugin, ClueGO [36]. The MYCN-activated genes were enriched in the regulation of the cell cycle and RNA processing, and the MYCN-repressed genes were significantly related to the processes of signal transduction, cell morphogenesis and cell differentiation (Figure 2C and 2D). These data reveal that MYCN has pleiotropic roles in NB.

### MYCN-regulated genes have prognostic value in NB patients with MYCN-non-amplification

An unsupervised clustering analysis of the MYCN-regulated genes indicated that the expression signatures of MYCN-regulated genes were strongly associated with MYCN status and NB risk type (Figure 2E). Although MYCN amplification is well known to be a poor prognostic marker in NB, we wondered whether these signatures could be used to identify subtypes of MYCN-non-amplified NB patients. We performed robust *k*-means clustering (*k* = 2) over the MYCN-regulated genes to separate MYCN-non-amplified patients into two groups and Kaplan-Meier analysis to compare the survival rate. Kaplan-Meier curves revealed that the event-free survival rates differed significantly between the two groups (Log-rank test, *p* = 1.18E−6; Figure 2F). This suggests that MYCN is involved in tumorigenesis of MYCN-non-amplified NB.

### The complexity of MYCN regulatory networks via regulating other transcription factors

Notably, only ~41% of the MYCN-correlated genes were bound by MYCN. This suggests that the remaining MYCN-correlated genes were regulated by other TFs driven by MYCN. To clarify these relationships, we obtained 1,484 TFs or proteins containing DNA binding domains from UniProt, and found that a significant proportion of the MYCN-regulated genes coded for TFs or proteins with a DNA binding domain (107 out of 874, *p <* 0.001, hypergeometric test). Furthermore, we examined the correlation between the expression of MYCN-regulated TFs and MYCN-correlated genes. If the MYCN-correlated genes were also regulated by MYCN-regulated TFs, their expression would be strongly correlated with that of the TFs. We computed the Spearman correlation coefficients between the MYCN-correlated genes and the TFs as a measure of their expression correlation. In total, 107 MYCN-regulated TFs tended to have significantly higher correlations with MYCN-correlated genes than with non-MYCN-correlated genes (Supplementary Figure S5). Based on the same criterion as before (|Spearman correlation coefficient| ≥ 0.3 for both profiling methods) to identify the correlations, each MYCN-correlated gene was coexpressed with at least one MYCN-regulated TF (Supplementary Table S2). This indicates that the MYCN-correlated genes without MYCN-bound signals were regulated indirectly by MYCN.

### Association of MYCN binding sites with gene regulation

**Figure 3:**
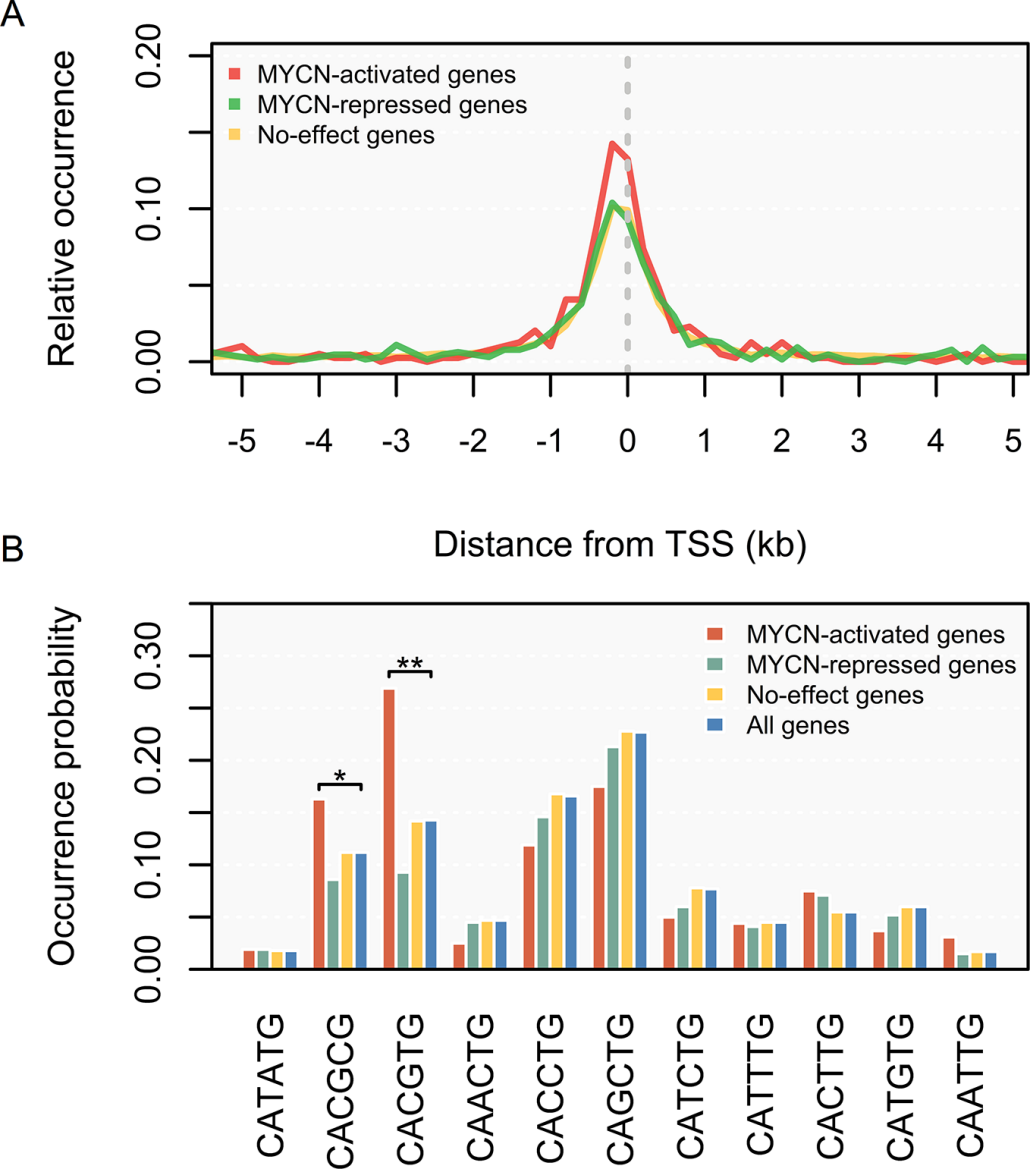
Distribution of MYCN binding and binding E-box sequences according to the direction of regulation by MYCN (**A**) Distance distribution of MYCN binding sites relative to transcription start sites (TSSs). Relative occurrence of MYCN binding peaks per 200-bp bin corresponding to the 5-kb region flanking the TSSs of MYCN-activated genes (red), MYCN-repressed genes (green), and no-effect genes (yellow). (**B**) Usage of E-box sequences by MYCN. Wilcoxon signed-rank test: *p < 0.05, **p < 0.01.

We then investigated whether the MYCN binding sites could reveal the role of MYCN in the genes it regulates. First, we re-examined the distribution of MYCN binding relative to genes and found that the MYCN binding sites were significantly enriched on the TSSs of MYCN-activated genes, relative to those of MYCN-repressed genes (*p <* 0.001, KS test; Figure 3A). This suggests that MYCN binds preferentially to up-regulated genes [33]. Next, we used the MYCN binding sequences to further address the sequence specificity of MYCN regulation. We examined all possible variants of the generic E-box motif (CANNTG). Significance was assessed using the *p*-value derived from Fisher's exact test. As illustrated in Figure 3B, we found that MYCN exhibited significant selection of the CACGTG (*p* = 4.6E–7) and CACGCG (*p* = 0.038) motifs in the promoters of MYCN-activated genes. However, none of the motifs were enriched in the promoters of MYCN-repressed genes. In c-MYC, the top two high-affinity binding motifs are CACGTG and CACGCG [37], identical to the enriched motifs in the MYCN-activated promoters. This indicates that MYCN behaves principally as an activator, while repressing its target genes by interacting or cooperating with other regulators.

### Gene regulation by MYCN is coordinated with other regulators

**Figure 4:**
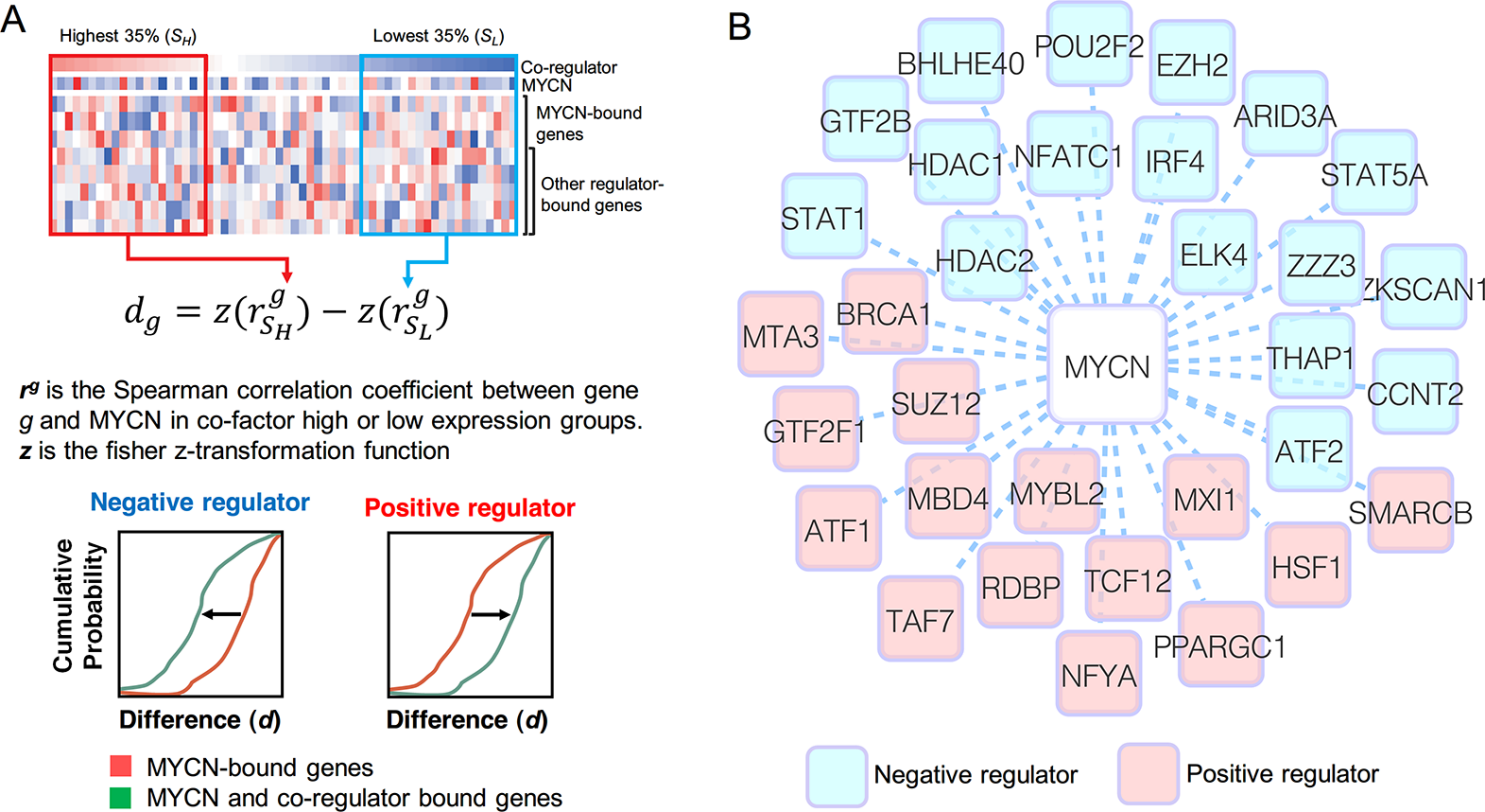
Inference of MYCN co-regulators (**A**) The schematic illustration of the method we used to infer MYCN co-regulators. See text for full details. (**B**) MYCN and its co-regulators illustrated as a graph. The blue and red nodes denote the negative and positive regulators, respectively.

MYCN might regulate gene expression by interacting or cooperating with other regulators. To understand the MYCN regulatory network in NB, it is necessary to identify MYCN's co-regulators. We proposed a computational method to infer potential MYCN co-regulators (Figure 4A). The main concept of this method is that the presence or absence of a co-regulator might alter the correlation between MYCN and its regulated genes. Using a *p*-value threshold of 0.05 and a consistent correlation pattern according to both types of gene expression data, we identified 32 potential MYCN co-regulators: 15 positive regulators and 17 negative regulators (Figure 4B). The distributions of the correlation differences of all inferred MYCN co-regulators are shown in Supplementary Figure S6.

Several MYCN co-regulators have been reported previously. For example, MYCN can repress genes through recruitment of HDAC1 and HDAC2 [39, 40], which were predicted as negative regulators in our analysis. EZH2, inferred as a negative MYCN co-regulator, has been demonstrated to physically interact with MYCN to repress tumor suppressor genes [41]. Another interesting case is MXI1. MXI1 binds MAX and E-box motifs such as c-MYC, but functions as a transcriptional repressor [42, 43]. Therefore, it is hypothesized that MXI1 antagonizes MYCN activity, as it does for c-MYC [44, 45]. In our analysis, however, MXI1 was identified as a positive regulator in MYCN regulatory networks. Although some studies have demonstrated that overexpression of MXI1 inhibits MYCN-dependent cell proliferation and activates apoptosis via a pathway independent of MYCN in NB cells [45, 46], there is no evidence that MXI1 directly represses MYCN-regulated genes. In addition, one report showed that MYCN activated MXI1 expression [47]. Overall, these findings suggest that although MXI1 might compete with MYCN for binding sites, the effect of MYCN might be greater than that of MXI1. Consequently, our analysis revealed MXI1 as an activator.

Some regulators might be indirectly coordinated with the MYCN regulatory network. ATF1, referred to as a positive regulator, has been demonstrated to increase expression of MYCN in spermatogonial stem cells [48] and gingival fibroblasts [49]. Another example is STAT1, which was identified as a negative regulator in our analysis. It is known that the c-MYC promoter region contains STAT1 binding sites, and that STAT1 increases and maintains the basal expression of MYC during tumorigenesis [50, 51]. We examined the ENCODE ChIP-seq dataset in the UCSC genome browser, but found no STAT1 binding signal in the MYCN promoter region. Additionally, many studies observed that MYCN and c-MYC may regulate each other's expression levels [20, 52, 53]. Therefore, we speculated that STAT1 might negatively regulate the MYCN regulatory network by inducing MYC.

### Identification of MYCN-regulated microRNAs

To identify MYCN-regulated miRNAs, we first carried out a small RNA-seq analysis of MYCN-knockdown SK-N-BE(2)-C cells. Two independent MYCN knockdown experiments were performed, and each was analyzed on a separate small RNA-seq. To identify differentially expressed miRNAs, the expression profiles of SK-N-BE(2)-C cells transfected with siRNA against MYCN (low MYCN) were compared with cells treated with a non-targeting control (high MYCN). We identified 45 differentially expressed miRNAs corresponding to 49 loci: 26 up-regulated and 19 down-regulated miRNAs (Table 1). Next, we examined whether these miRNAs were directly regulated by MYCN. Because the TSSs of the miRNAs were unclear, we used predicted TSSs from miRStart [54] and PROmiRNA [55]. Therefore, the promoter of a miRNA was defined as the genomic region from 10 kb upstream of the predicted TSS to the start site of the miRNA precursor, and if an MYCN binding site fell in the promoter region of a miRNA, this miRNA was considered as MYCN-regulated miRNA. Additionally, if the host gene of a miRNA was bound by MYCN, this miRNA was also considered as a MYCN-regulated miRNA. Based on these criteria, we identified 28 out of 49 miRNA as possible direct transcription targets of MYCN. These 28 miRNA loci contained 12 MYCN-activated miRNAs and 12 MYCN-repressed miRNAs (Table 2).

**Table 1:** Differentially expressed miRNAs of MYCN knockdown

miRNA	Stem-loop sequence	Average normalized read count of siMYCN	Average normalized read count of control	log2 fold-change (siMYCN/control)	Probability
miR-124-3p	mir-124-1 mir-124-2 mir-124-3	314.8	28.0	3.49	0.97
miR-410-3p	mir-410	149.8	7.9	4.24	0.96
miR-1307-3p	mir-1307	73.4	569.2	−2.95	0.96
miR-33a-5p	mir-33a	42.3	4.7	3.18	0.93
miR-1307-5p	mir-1307	18.5	2.1	3.16	0.90
miR-27b-3p	mir-27b	589.0	142.1	2.05	0.90
miR-1268a	mir-1268a	80.2	16.0	2.32	0.89
miR-27a-3p	mir-27a	68.2	14.5	2.23	0.88
mir-873	mir-873	66.4	14.1	2.23	0.88
miR-92a-1-5p	mir-92a-1	108.4	377.0	−1.80	0.87
miR-331-3p	mir-331	32.4	7.1	2.19	0.85
miR-1268b	mir-1268b	89.8	25.6	1.81	0.85
miR-130a-3p	mir-130a	78.4	21.5	1.87	0.85
miR-377-3p	mir-377	18.8	4.1	2.19	0.84
miR-221-5p	mir-221	3.7	16.9	−2.19	0.83
miR-423-5p	mir-423	2111.4	5697.1	−1.43	0.81
miR-181d-5p	mir-181d	167.7	505.2	−1.59	0.81
miR-345-5p	mir-345	10.6	2.7	1.96	0.81
miR-887-3p	mir-887	3.6	12.2	−1.77	0.80
mir-92b	miR-92b-5p	30.7	90.9	−1.56	0.79
miR-487b-3p	mir-487b	249.7	96.9	1.37	0.78
miR-181b-5p	mir-181b-1 mir-181b-2	862.6	2181.6	−1.34	0.75
miR-296-5p	mir-296	11.5	3.8	1.59	0.72
miR-496	mir-496	10.3	3.5	1.54	0.72
miR-320a	mir-320a	11660.9	24018.5	−1.04	0.70
miR-323a-3p	mir-323a	1147.6	537.4	1.09	0.70
miR-7-5p	mir-7-1 mir-7-2 mir-7-3	38.4	91.5	−1.25	0.70
miR-505-5p	mir-505	16.3	39.2	−1.27	0.67
miR-93-5p	mir-93	714.4	361.2	0.98	0.67
miR-221-3p	mir-221	131.1	257.8	−0.98	0.66
miR-181a-5p	mir-181a-1 mir-181a-2	3045.0	5580.8	−0.87	0.65
miR-2110	mir-2110	4.0	10.1	−1.34	0.65
miR-760	mir-760	12.8	28.5	−1.15	0.64
miR-377-5p	mir-377	59.8	29.6	1.02	0.64
miR-363-3p	mir-363	13.2	5.4	1.29	0.64
miR-412-5p	mir-412	4.8	11.2	−1.23	0.63
miR-24-3p	mir-24-1 mir-24-2	959.0	547.1	0.81	0.63
miR-330-3p	mir-330	58.2	106.3	−0.87	0.62
miR-1301-3p	mir-1301	106.8	185.2	−0.79	0.62
miR-382-5p	mir-382	266.3	157.3	0.76	0.61
miR-222-3p	mir-222	578.5	959.9	−0.73	0.61
miR-323b-3p	mir-323b	10.1	4.7	1.09	0.61
miR-25-3p	mir-25	1434.6	875.3	0.71	0.61
miR-376c-3p	mir-376c	70.1	39.8	0.82	0.60
miR-361-5p	mir-361	16.6	8.4	0.99	0.60

The list is sorted by “probability”. The probability derived from NOISeq indicates the “probability of differential expression”. The raw read counts were normalized by upper-quartile method.

**Table 2:** List of MYCN-regulated microRNAs

miRNA	Stem-loop sequence	Regulation of MYCN^&^	Number of target genes	3-node motif	4-node motif
miR-124-3p	mir-124-1 mir-124-3	R	2079	138^*^	296
miR-33a-5p	mir-33a	R	1212	51	214^*^
miR-1307-5p	mir-1307	R	0	0	0
miR-1268a	mir-1268a	R	31	1	1
miR-27a-3p	mir-27a	R	2264	115	303
miR-27b-3p	mir-27b	R	2307	115	308
miR-345-5p	mir-345	R	72	10^*^	7
miR-1268b	mir-1268b	R	8	0	0
miR-296-5p	mir-296	R	169	16^*^	31
miR-93-5p	mir-93	R	1916	130^*^	352^*^
miR-24-3p	mir-24-1 mir-24-2	R	1557	94^*^	365^*^
miR-25-3p	mir-25	R	1247	62	188
miR-181a-5p	mir-181a-1 mir-181a-2	A	1321	76^*^	307^*^
miR-330-3p	mir-330	A	689	45^*^	94
miR-320a	mir-320a	A	1204	74^*^	292^*^
miR-760	mir-760	A	250	14	22
miR-7-5p	mir-7-1 mir-7-2	A	1909	89	274
miR-181b-5p	hsa-mir-181b-1 hsa-mir-181b-2	A	1348	79^*^	316^*^
miR-2110	mir-2110	A	213	17^*^	30
miR-92b-5p	mir-92b	A	4	0	1
miR-181d-5p	mir-181d	A	1257	73^*^	286^*^
miR-887-3p	mir-887	A	8	0	2
miR-92a-1-5p	mir-92a-1	A	49	2	16
miR-1307-3p	mir-1307	A	2	0	0

* denotes *p*-value < 0.05.

& R: repression; A: activation.

Several pairs of miRNAs shared a common gene promoter: mir-27a and mir-24-2; mir-27b and mir-24-1; mir-25 and mir-93; mir-181a-1 and mir-181b-1; and mir-181a-2 and mir-181b-2. In addition, miRNAs in the same pair were regulated in the same direction. Interestingly, mir-1307 was differentially expressed under MYCN knockdown, but showed reversed regulation in the 5p/3p species. Although the reverse direction of 5p/3p coexpression has been reported in several studies [56, 57], the mechanism and biological significance of preferred arm selection remains unknown.

To obtain the miRNA-regulated genes, we compiled one experimentally validated and 11 predicted miRNA target databases and assigned a confidence score to each miRNA-target gene pair based on the number of supported predictions. With respect to the distribution of the confidence scores, there was a substantial drop at score 0.3 (Supplementary Figure S7). Therefore, in addition to the experimentally validated miRNA-target interactions, only the miRNA-target interactions, supported by at least four databases (i.e. confidence score > 0.3), were considered for further analysis. Each MYCN-regulated miRNA had an average of 918 targets (Table 2).

### Interplay between MYCN and microRNA regulatory networks

**Figure 5:**
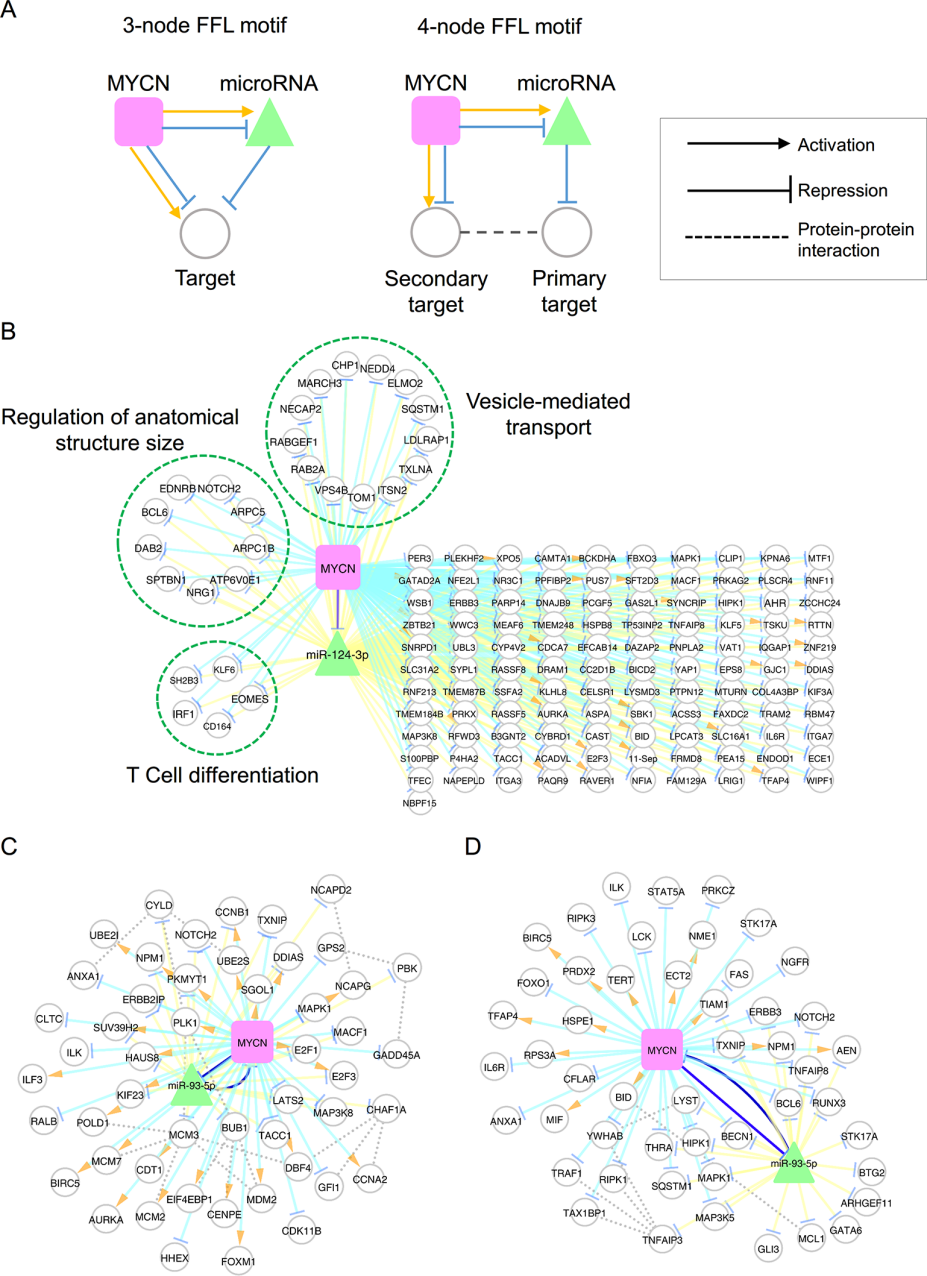
MYCN and microRNA co-regulatory motifs (**A**) Schematic illustration of three- and four-node feed-forward loop (FFL) motifs. (**B**) The co-regulatory network of MYCN and miR-124-3p. Genes involved in the same function are grouped in a dashed circle. (C–D) The co-regulatory networks of MYCN and miR-93-5p based on the following enriched functions: cell cycle (**C**); and cell death (**D**).

Since transcriptional regulation of TFs is tightly coupled with the post-transcriptional regulation of miRNAs, we investigated the coordination between MYCN and its regulated miRNAs by utilizing three- and four-node feed-forward loops (FFLs; Figure 5A), which are frequently observed network motifs in various regulatory networks [58–60]. To identify the three-node motifs, we assessed the significant common targets of miRNA and MYCN by using the hypergeometric test. For the four-node motifs, we assessed whether MYCN-regulated genes were more than representatively physically connected with miRNA targets, using the permutation test. Here, the miRNA targets should also be MYCN-correlated genes.

Using a *p*-value of 0.05 as a threshold, 11 and 7 miRNAs formed three- and four-node motifs with MYCN, respectively (Table 2). Six miRNAs significantly formed both three- and four-node motifs with MYCN: miR-93-5p, miR-24-3p, miR-181a-5p, miR-320a, miR-181b-5p, and miR-181d-5p. Among these, expression of miR-181a-5p, miR-181b-5p, and miR-320a has been reported to be associated with MYCN status and unfavorable NB [12, 13, 61].

Our previous study demonstrated that the knockdown of miR-124-3p promotes MYCN-non-amplified NB cell differentiation, cell cycle arrest and apoptosis [62]. Therefore, we were interested in the coordination between miR-124-3p and MYCN. Because miR-124-3p only significantly forms three-node motifs with MYCN, we focused on the common targets of miR-124-3p and MYCN. There were 138 such common targets, of which 26 and 112 were activated and repressed by MYCN, respectively (Figure 5B). GO enrichment analysis revealed that miR-124-3p and MYCN co-regulated genes were involved in vesicle-mediated transport, regulation of anatomical structure size, and T cell differentiation, consistent with miR-124-3p-induced phenotypes [62, 63]. Interestingly, the regulation of genes in the same functional categories was coherent, i.e. they were all repressed by MYCN.

miR-93-5p has been documented to play a role as an oncogenic miRNA in many tumor types [64–66], but has not been investigated in NB. miR-93-5p is hosted in MCM7, which is regulated by MYCN, and is also predicted to target MYCN (confidence score: 1.0). Our analysis revealed many genes that were co-regulated by miR-93-5p and MYCN. The collection of miR-93-5p and MYCN co-mediated three- and four-node motifs comprised 369 genes and 770 interactions. To dissect this co-regulatory network, we performed GO enrichment analysis and identified function-specific sub-networks. The GO enrichment analysis revealed that the majority of miR-93-5p and MYCN co-regulated genes were involved in the cell cycle and cell death processes (Figure 5C and 5D). One interesting FFL in the cell-cycle network is the MYCN/E2F1/miR-93-5p circuit. E2F1 plays a critical role in the control of cell cycle progression in many cancer types and is the known target of miR-93-5p [67]. In this FFL, MYCN activates E2F1 but represses miR-93-5p to maintain E2F1 at a high expression level. Another interesting motif is MYCN/MCM2-7/MCM3/miR-93-5p. MCM2, MCM3, and MCM7 are the members of the minichromosome maintenance (MCM) complex, and are essential in the initiation of DNA replication during the cell cycle [68]. In this circuit, MYCN activates MCM2 and MCM7 expression and inhibits miR-93-5p expression to avoid the degradation of MCM3, which forms the MCM complex with MCM2 and MCM7. These interactions might be used to ensure that the NB cell cycle functions normally. Similarly, the motif of MYCN/BID/MCL1/miR-93-5p might be an important circuit for the inhibition of apoptosis because MCL1 interacts with BID to inhibit the induction of cytochrome c release [69]. Together, these findings suggest that miR-93-5p is a useful target for inhibiting the MYCN-induced pathway.

### Integrative regulatory networks reveal potential therapeutic targets in NB

Finally, we examined the number of MYCN-regulated miRNAs targeting each MYCN-regulated gene. If the MYCN-regulated genes are critical in NB tumorigenesis, they might be targeted by a significant number of MYCN-regulated miRNAs to maintain their expression level. To identify this type of MYCN-regulated genes, we applied the hypergeometric test with Benjamini-Hochberg false discovery rate (FDR) correction. A total of 116 out of 874 MYCN-regulated genes were significantly targeted by MYCN-regulated miRNAs (adjusted *p*-value < 0.05; Supplementary Table S3). Interestingly, a large proportion of these genes (81%, 94/116) were repressed by MYCN. Moreover, among these enriched targets of MYCN-regulated miRNAs, some of them, such as KLF6 [70], RASSF8 [71], TGFBR3 [72], ARNTL [73], NDRG4 [74], PHTF1 [75], HIPK1 [76], PTGER4 [77], HECA [78], and EOMES [79], are known as tumor suppressor genes and are suggested as therapeutic targets in other cancer types. This implies that MYCN and MYCN-regulated miRNAs act together to down-regulate tumor suppressor genes.

**Figure 6:**
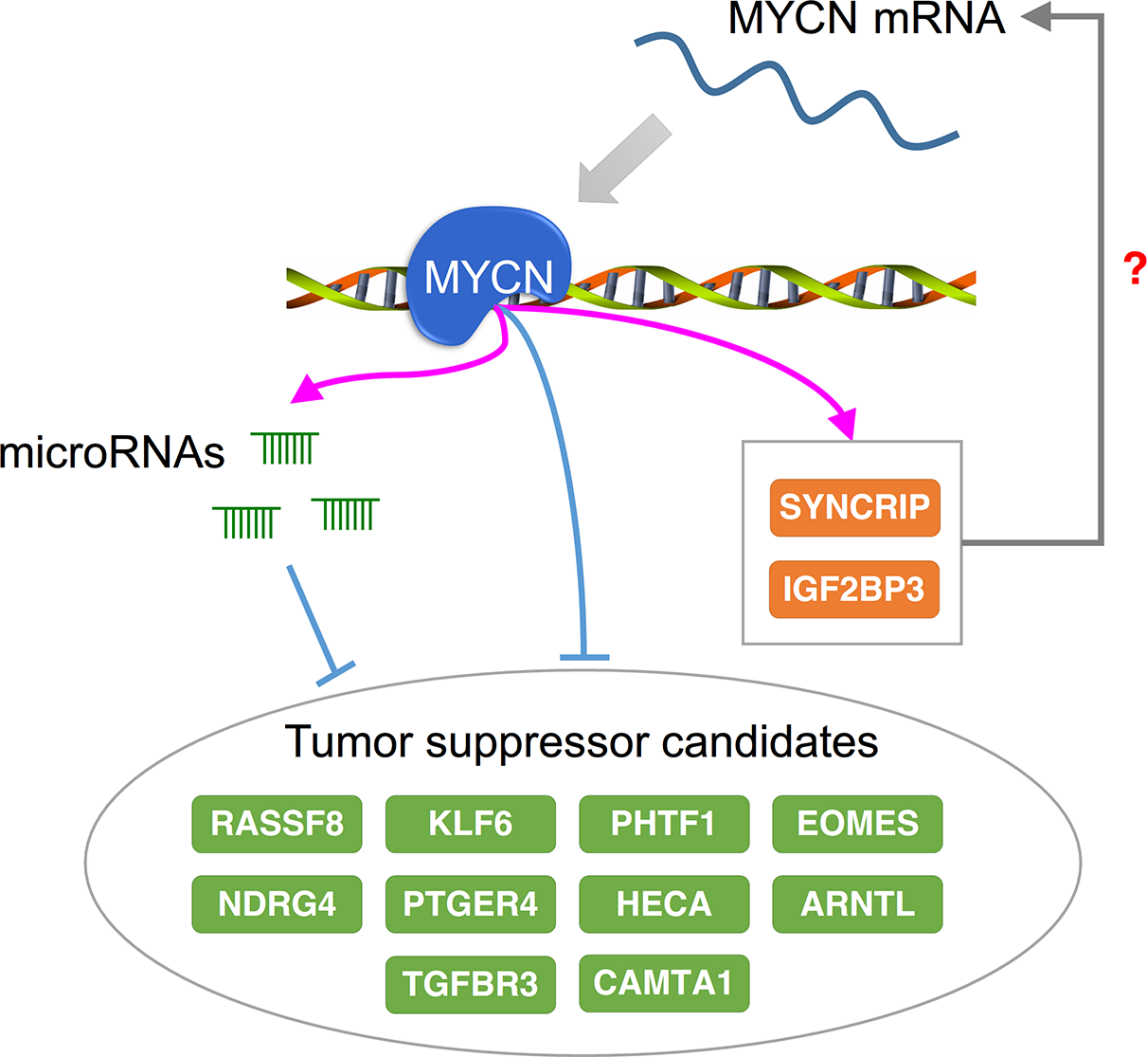
Potential therapeutic targets of NB with respect to regulation by MYCN A group of genes repressed by MYCN and MYCN-regulated microRNAs has been reported as tumor suppressor candidates in NB and other cancer types. The RNA binding proteins SYNCRIP and IGF2BP3 are up-regulated by MYCN and might bind to MYCN or MYCN-regulated genes to maintain their mRNA stability. Orange and green nodes represent the MYCN-activated and -repressed genes, respectively.

The identification of these enriched targets of MYCN-regulated miRNAs might benefit the development of NB therapy (Figure 6). As described previously, several of the targets are tumor suppressor genes that have been reported for other cancer types, and some of them might also be tumor suppressor candidates in NB. For example, calmodulin binding transcription activator 1 (CAMTA1) is located on chromosome 1p, which is often deleted in NB [80], and overexpression of CAMTA1 suppresses cell growth and induces neurite-like processes and markers of neuronal differentiation in NB cells [81]. In our MYCN regulatory network, CAMTA1 was repressed by MYCN and also targeted by several MYCN-activated miRNAs, including miR-181a-5p, miR-181b-5p, and miR-181d-5p. Because the deletion of 1p and MYCN amplification generally co-occurs in NB patients [4], this reveals the importance of CAMTA1 in NB. Some of the enriched targets might play a role in maintaining the stability of MYCN regulatory networks. Synaptotagmin binding, cytoplasmic RNA interacting protein (SYNCRIP) and insulin-like growth factor-2 mRNA-binding protein 3 (IGF2BP3) are both RNA binding proteins that are activated by MYCN and have opposing functions in controlling neuronal fates [82]. SYNCRIP has been reported to be essential in ensuring the stabilization of c-MYC mRNA [83]. Similarly, although there is no direct evidence that IGF2BP3 interacts with MYCN or c-MYC, IGF2BP1, a member of the IGF2BP family, can stabilize c-MYC mRNA and elevate the protein expression of c-MYC [84]. Although it is unclear whether SYNCRIP and IGF2BP3 also play a role in stabilizing MYCN mRNA, we speculate that they might stabilize MYCN mRNA or other genes underlying MYCN regulatory networks. To elucidate the suitability of these potential therapeutic targets of NB, advanced experiments are required.

## MATERIALS AND METHODS

### Cell culture

SK-N-BE(2)-C cells were obtained from ATCC (American Type Culture Collection, Manassas, VA). Cells were grown in DMEM/F12 (Gibco Laboratories, Grand Island, NY) supplemented with 10% fetal bovine serum (Gibco Laboratories) under 37°C and 5% CO_2_ and routinely passaged when 80–90% confluent. All cells were free of mycoplasma, as determined by a PCR-based mycoplasma detection method (MBI Fermentas, Vilnius, Lithuania).

### Chromatin immunoprecipitation (ChIP)-sequencing and analysis

The ChIP assay was performed using EZ-Magna ChIP A (Upstate-Millipore, Billerica, MA, USA). Cells were cross-linked in 1% formaldehyde (Sigma-Aldrich, St Louis, MO, USA) for 30 min. Nuclear lysates were extracted and the chromatin fraction was sheared to 200–500-bp fragments using an ultrasonic probe (Labsonic M, Sartorius, Tagelswangen, Switzerland). Immunoprecipitation was performed overnight at 4°C using 1 μg of anti-MYCN antibody (Abcam, Cambridge, UK) or 1 μg of anti-mouse IgG1κ antibody (Abcam) as a control. After washing to remove nonspecific DNA binding, the protein/DNA complexes were eluted and reverse cross-linked to free DNA fragments as described in the manual. Purified fragmented DNA was subjected to ChIP-seq analysis to identify the MYCN binding regions.

DNA fragments (150–400 bp long) were gel–purified, and the adaptors were ligated to both ends of fragments. The PCR-amplified DNA libraries were quantified on an Agilent 2100 Bioanalyzer (Agilent Technologies, Palo Alto, CA, USA) and diluted for cluster generation. The ChIP-seq libraries were assayed by single-end sequencing on an Illumina HiSeq sequencing platform at the Beijing Genomics Institute (BGI).

The 49-nt reads were aligned to the human genome GRCh37 using Bowtie 2 [85]. Only those reads that mapped uniquely to the genome were retained for binding-peak identification. The Model-based Analysis of ChIP-seq (MACS version 1.4.2) algorithm [86] was used to identify the enriched regions with a *p*-value cutoff of 0.001 and modified parameters (bw = 500).

### ChIP-qPCR

To validate the ChIP-seq results, candidate MYCN binding sequences near the transcription start site of the miRNA or miRNA-hosted gene were selected. After ChIP, the purified DNA fragments from the MYCN antibody and isotype control IgG were quantitatively amplified using iQ SYBR Green Supermix (Bio-Rad Laboratories) with a Bio-Rad CFX-96 thermocycler (Bio-Rad Laboratories), using the following PCR protocol: 2 min at 95°C, 40 cycles of 10 s at 95°C, and 30 s at 55°C. Specific primers for each amplicon are listed in Supplementary Table S4. Fold enrichment of a given antibody *k* (*FE*_k_) was calculated using the following equation:

$$FE_{k}=2^{-\left(Ct_{k}-Ct_{i}\right)},$$

where *Ct*_k_ is the readout threshold value (*Ct*) of the selected amplicon immunoprecipitated from antibody *k*, and *Ct*_i_ is that of the IgG control antibody.

### Transcriptome of neuroblastoma

NB patient gene expression data were obtained from the Sequencing Quality Control (SEQC) project (GSE47792). This project generated gene expression profiles from 498 primary NB patients using RNA-seq (GSE62564) and microarray (GSE49710). Both types of expression data were used as independent datasets.

### Survival analysis

K-means clustering was used to stratify the MYCN-non-amplified patients into two distinct groups according to their gene expression of 874 MYCN-regulated genes. To obtain robust groups, we performed 1000 time K-means with different initial centers and determined the conserved group of each sample. Kaplan-Meier survival analysis was used to compare the survival rate between groups that emerged from this k-means clustering. All analyses were performed with R software.

### Transcriptional factor binding sites

Transcriptional factor binding sites (TFBSs) were identified from the data based on the ChIP-seq experiments for 161 transcription factors across 91 cell types using the ENCODE project. We downloaded TFBSs, via the Table Browser of the UCSC Genome Bioinformatics website (http://genome.ucsc.edu/), from the “Txn Factor ChIP” track (table name is wgEncodeRegTfbsClusteredV3).

### Inference of co-regulators of MYCN

The method inspired by the modulator inference by network dynamics (MINDy) algorithm [38] was proposed to infer potential MYCN co-regulators. As illustrated in Figure 4A, for a given regulator, the samples were classified into two subsets, *S_H_* and *S_L_*, based on the expression value of the regulator. Here, we used 35% as a threshold value to separate the high and low regulator expression samples. For a given MYCN-bound gene *g*, we calculated its expression correlation with MYCN in subset *S_H_* and *S_L_*, i.e. $r_{S_{H}}^{g}$ and $r_{S_{L}}^{g}$, respectively. We used the Spearman correlation coefficient to measure the expression correlation. The correlation difference of gene *g* between *S_H_* and *S_L_* was calculated using the following formula:

$$d_{g}=z\left(r_{S_{H}}^{g}\right)-z\left(r_{S_{L}}^{g}\right),$$

where *z* is the Fisher *z*-transformation function defined as:

$$2\left(r\right)=\frac{1}{2}\text{ln}\left(\frac{1+r}{1-r}\right)\sqrt{\frac{N-3}{1.06}},$$

where *N* is the sample size. Finally, we determined whether the correlation difference distribution of genes bound by both MYCN and the regulator was significantly greater or less than that of MYCN-bound genes, using the Kolmogorov-Smirnov (KS) test. If the *p*-value was less than 0.05, this regulator was considered as a co-regulator of MYCN. Based on the distribution of correlation differences, the co-factors were classified into positive and negative regulators. The NB gene expression data generated by RNA-seq and microarray from the SEQC project and the ChIP-seq data for 161 regulators from the ENCODE project were used in this analysis.

### siRNA transfection

Cells (4 × 10^5^) were plated into 6-well plates and transiently transfected with 150 pmol double-stranded RNA (dsRNA) oligonucleotides against MYCN (Dharmacon, Lafayette, CO, USA; SMARTpool, J-003913-16, 5′-CGA GCUGGGUCACGGAGAU-3′; 5′-GAACCCAGACCUC GAGUUU-3′; 5′- GGACAGUGAGCGUCGCAGA-3′; 5′-CCUCCAUGACAGCGCUAAA-3′). Double-stranded oligonucleotide was diluted into 250 μl of serum-free DMEM, mixed with 250 μl serum-free DMEM containing 7.5 μl of Lipofectamine 2000 reagent (Invitrogen, Carlsbad, CA, USA), and incubated for 20 min at room temperature, before being added to the cells growing in 1.5 ml of complete medium. After 48 h of transfection, cells were harvested using 1 ml TRIzol reagent, and RNA was extracted using TRIzol reagent (Invitrogen) as indicated in the manufacturer's protocol.

### Small RNA sequencing and analysis

RNA concentration and purity were determined photometrically using a NanoDrop ND-1000 Spectrophotometer (NanoDrop Technologies Inc, Rockland, DE); absorbance was measured at 260 nm and the A260/A280 ratio was calculated. RNA integrity was evaluated using the Agilent 2100 Bioanalyzer (Agilent Technologies, Palo Alto, CA, USA). Total RNA (20 μg) was used for library construction following the protocol supplied with the Small RNA Sample Prep Kit (Illumina, San Diego, CA, USA), and Solexa sequencing was performed by the Beijing Genomics Institute (BGI) according to the manufacturer's instructions.

The raw reads were trimmed for an adaptor sequence using cutdapt [87], and reads shorter than 17 bases after trimming were discarded. We aligned reads to known human miRNA precursors (miRBase release 20) and counted the aligned reads for quantitative miRNA expression using the miRExpress analysis pipeline [88]. The -t parameter (alignment identity between query and reference sequences) for miRExpress was set to 0.9. The raw read counts were normalized by upper-quartile normalization. Differentially expressed miRNAs were identified using the NOISeq package from Bioconductor [89]. NOISeq differential expression statistics were calculated by comparing the M (the log_2_-ratio of two conditions) and D (the differences between two conditions) values against the noise distribution to obtain the “probability of differential expression”. We defined the MYCN-associated miRNAs as those with probability ≥ 0.6 and an average read count across two conditions of ≥ 100.

### Integration of miRNA-target relationships

We compiled 12 experimentally validated and predicted miRNA-target databases: miRTarBase [90], miRanda [91], TargetScan [92], picTar [93], PITA [94], miRDB [95], TargetMiner [96], DIANA-microT [97], RNA22 [98], CoMeTa [99], miRcode [100], and miRMap [101]. The miRNA names were mapped to miRBase (release 20), and the identifier for each target gene was mapped to Entrez Gene ID. After removal of redundancies, we obtained 8,226,628 miRNA-target relationships, between 2,037 miRNAs and 18,554 target genes. We then assigned a confidence score to each miRNA-target relationship based on the following rules: (1) if the relationship was curated in miRTarBase, which manually collects experimentally validated microRNA-target interactions from the literature, the confidence score was one; (2) if the relationship was not curated in miRTarBase but was supported by *n* prediction databases, the confidence score was *n*/10. If *n >* 10, the confidence score was nevertheless one. To restrict our analysis to high-confidence miRNA-target relationships, we considered only those with confidence scores ≥ 0.4.

### Assessment of MYCN-mediated microRNA feed-forward loop motifs

We considered two types of MYCN-mediated microRNA FFL motifs. The first was a three-node FFL motif comprising MYCN, a microRNA, and a common target gene. The second was a four-node FFL motif consisting of MYCN, a microRNA, a microRNA-target gene (primary target), and an MYCN-regulated gene (secondary target) that interacts with a primary target. Although the primary target is not directly regulated by MYCN, its expression might be associated with MYCN. Here, we specified that the primary target of the four-node motif had to be the MYCN-correlated gene.

For the three-node FFL motif, we applied the hypergeometric test to determine whether MYCN and the microRNA regulated a significant number of common genes. For the four-node FFL motifs, we performed the permutation test. The protein-protein interactions (PPIs) were collected from public databases and high-throughput experiments. For each microRNA, the number of PPIs connecting the primary and secondary targets was determined. Next, a random procedure was carried out by randomly drawing a set with the same number of primary targets as the set of MYCN-correlated genes, excluding the MYCN-regulated genes, and counting the number of PPIs connecting the random set and the secondary target. After running this procedure 1000 times, the empirical *p*-value was calculated as the proportion of random procedures for which the PPI number was larger than the observed value.

### Data availability

The raw reads from ChIP-seq and small RAN-seq generated in this study have been deposited at the Gene Expression Omnibus (GEO) under accession numbers GSE72640 and GSE72721.

## CONCLUSIONS

Through integration of heterogeneous regulatory data, this study reveals the complexity of the role of MYCN as a driving oncogene for neuroblastoma. We identified the potential regulators involved in the MYCN regulatory networks at various molecular levels, including DNA, mRNA, and miRNA. These valuable resources allow us to improve our understanding of MYCN regulation in neuroblastoma and help to develop diagnostic tools and effective therapeutic strategies for this cancer. Further dissection of the downstream effects of MYCN and identification of pivotal regulators are required to reach these goals. In particular, identifying the best target for inhibiting MYCN-driven tumorigenesis remains a challenge and requires further experimental verification.

## SUPPLEMENTARY MATERIALS FIGURES AND TABLES




