# Supplementary material for: Unveiling MYCN regulatory networks in neuroblastoma via integrative analysis of heterogeneous genomics data
Source: Oncotarget. 2016 May 6;7(24):36293–310. doi: 10.18632/oncotarget.9202 (PMC5095001; doi:10.18632/oncotarget.9202)
Supplement: Supplementary file 1 [file oncotarget-07-36293-s001.pdf]

Unveiling MYCN regulatory networks in neuroblastoma via integrative analysis of heterogeneous genomics data

Supplementary Materials

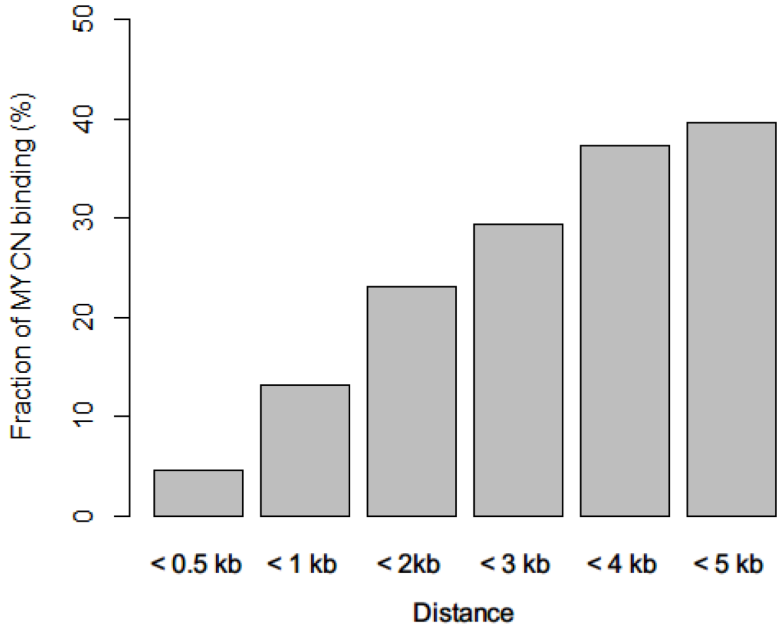

Supplementary Figure S1: The comparison of MYCN binding sites identified by our ChIP-seq data and Murphy's study. Murphy *et al.* performed anit-MYCN ChIP-chip experiments to identify the MYCN binding sites in Kelly, SK-N-AS, and TET21N neuroblastoma cell lines, and 256 common binding sites were identified [1]. Since the resolution of ChIP-seq and ChIP-chip are different, if the binding sites fell in a specific distance, we considered these binding sites were the same. With various distance values, the proportions of 256 sites overlapped by our datasets were calculated.

NME2 (NME/NM23 nucleoside diphosphate kinase 2)

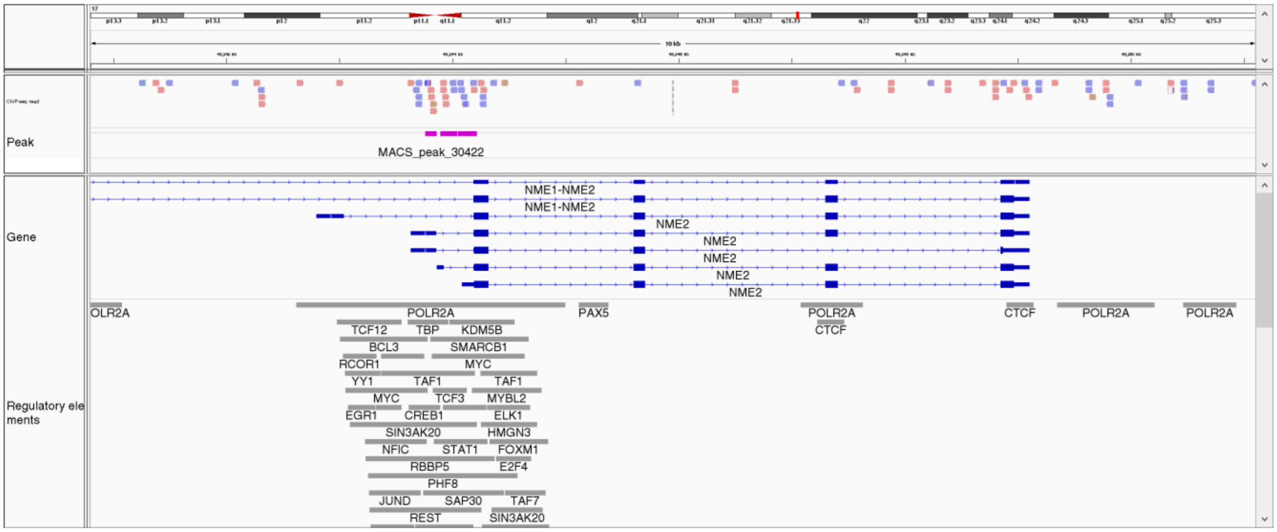

CRABP2 (cellular retinoic acid binding protein 2)

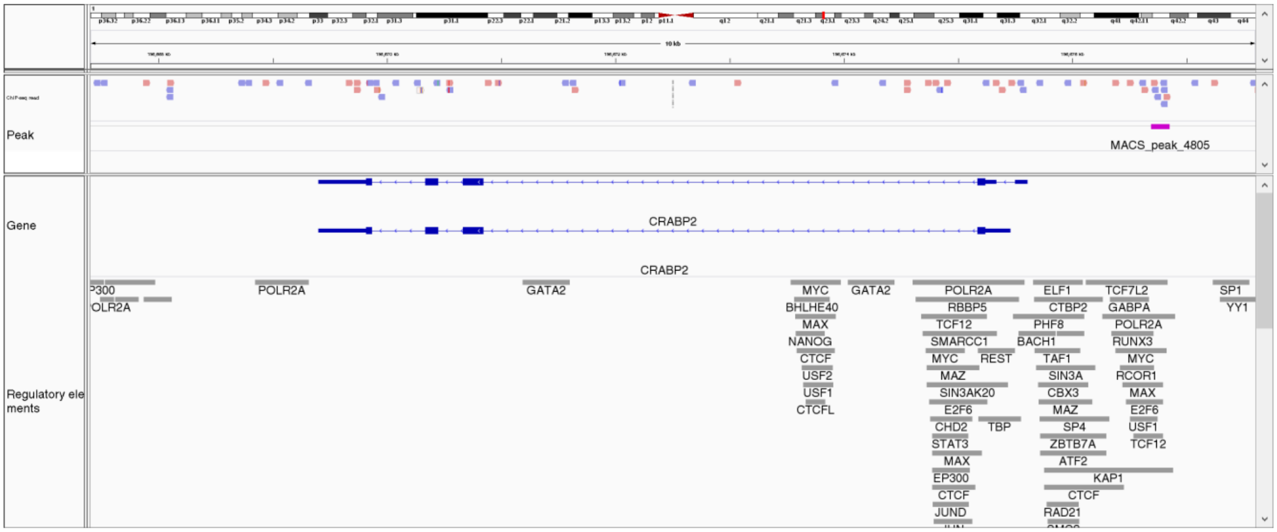

LIF (leukemia inhibitory factor)

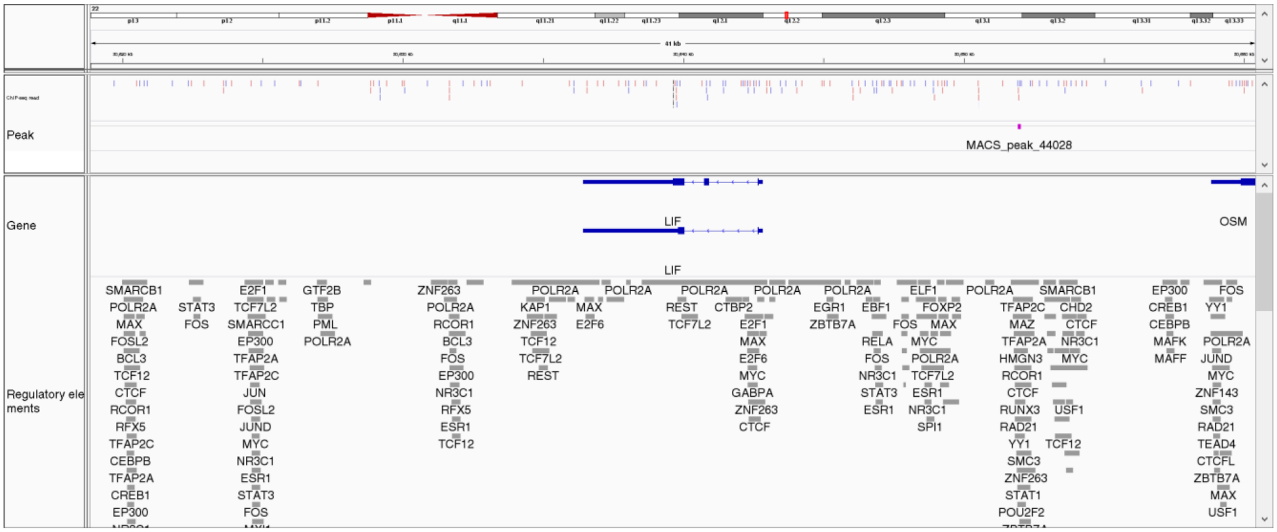

MDM2 (MDM2 proto-oncogene, E3 ubiquitin protein ligase)

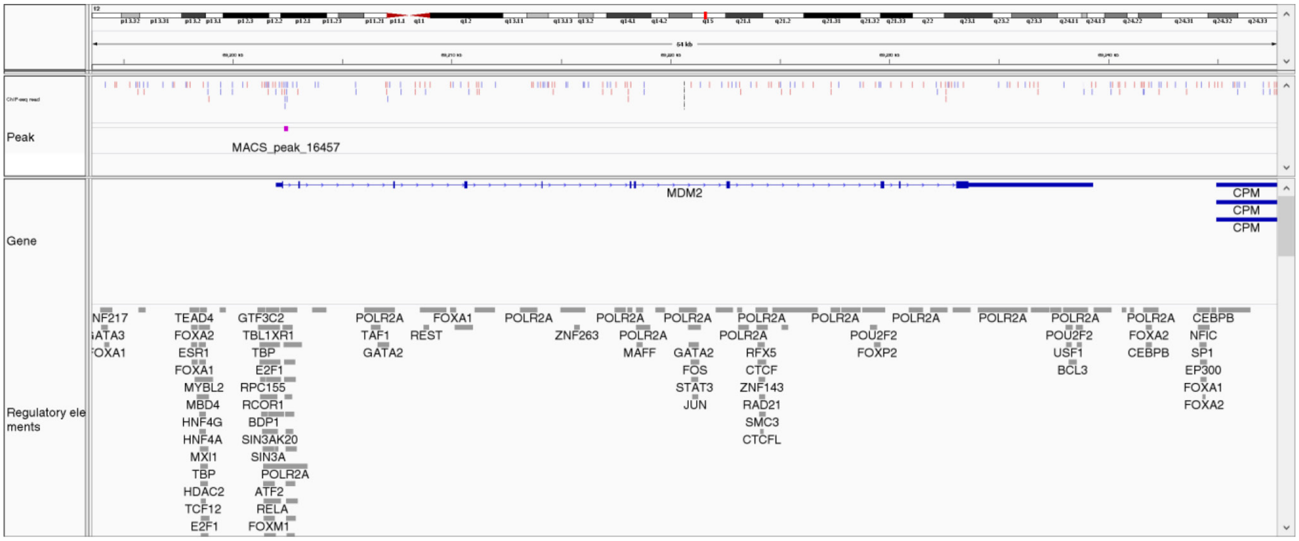

MIR17HG (miR-17-92 cluster host gene)

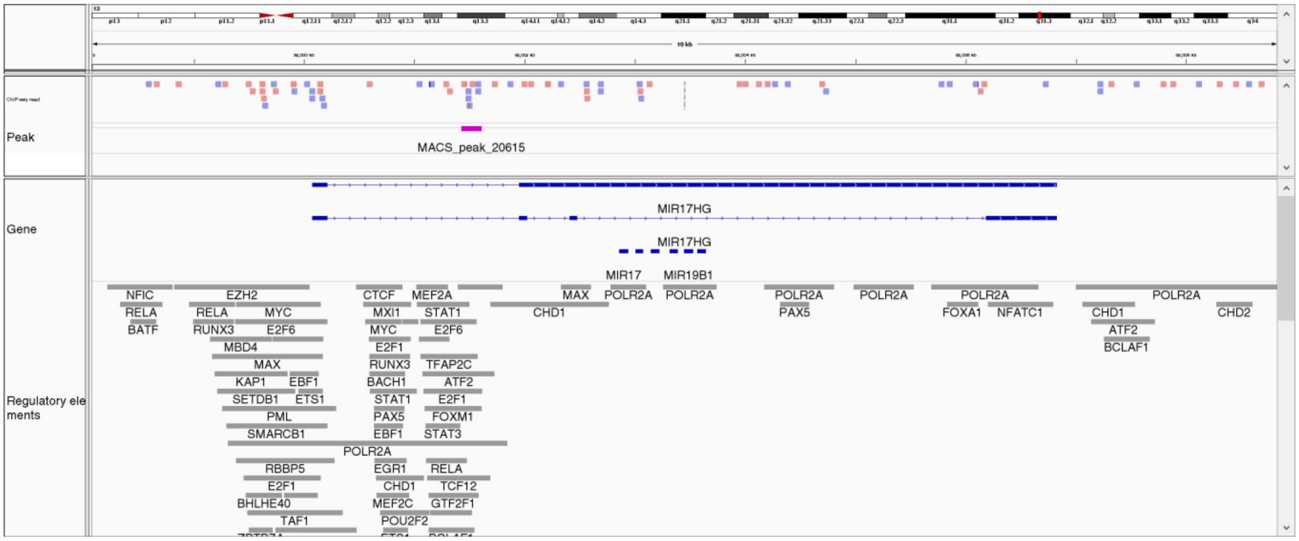

PRMT1 (protein arginine methyltransferase 1)

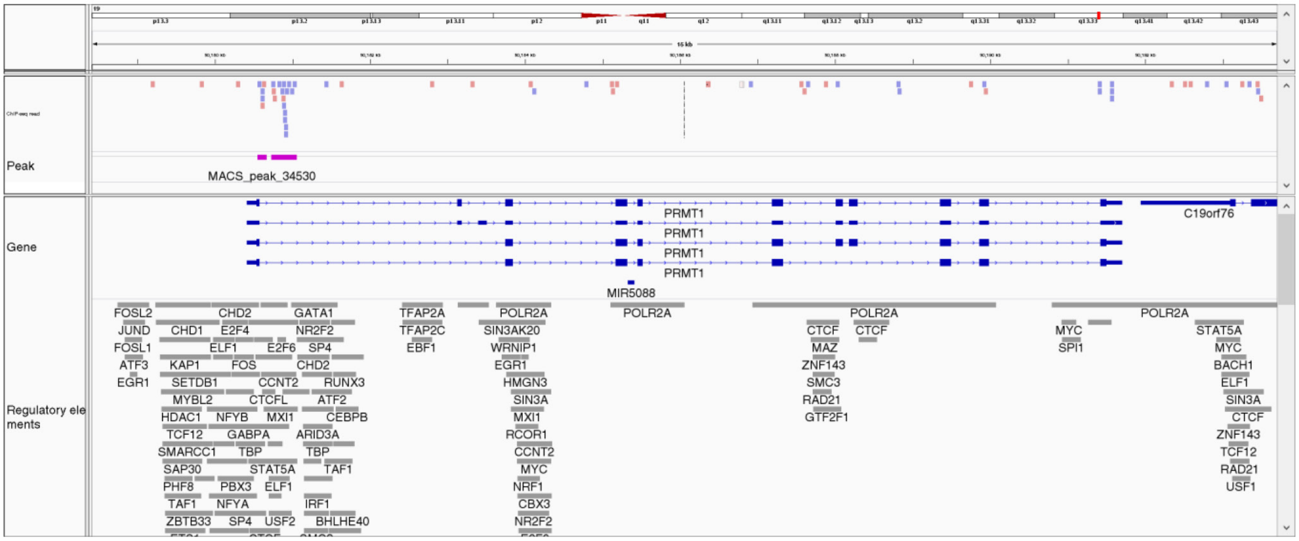

MCM7 (minichromosome maintenance complex component 7)

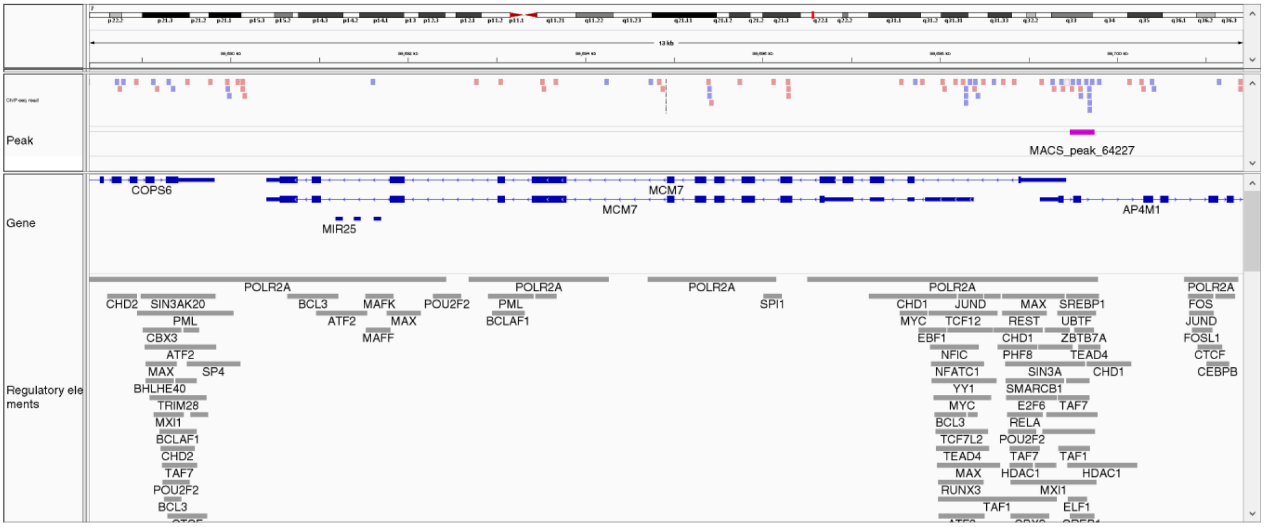

MCM8 (minichromosome maintenance complex component 8)

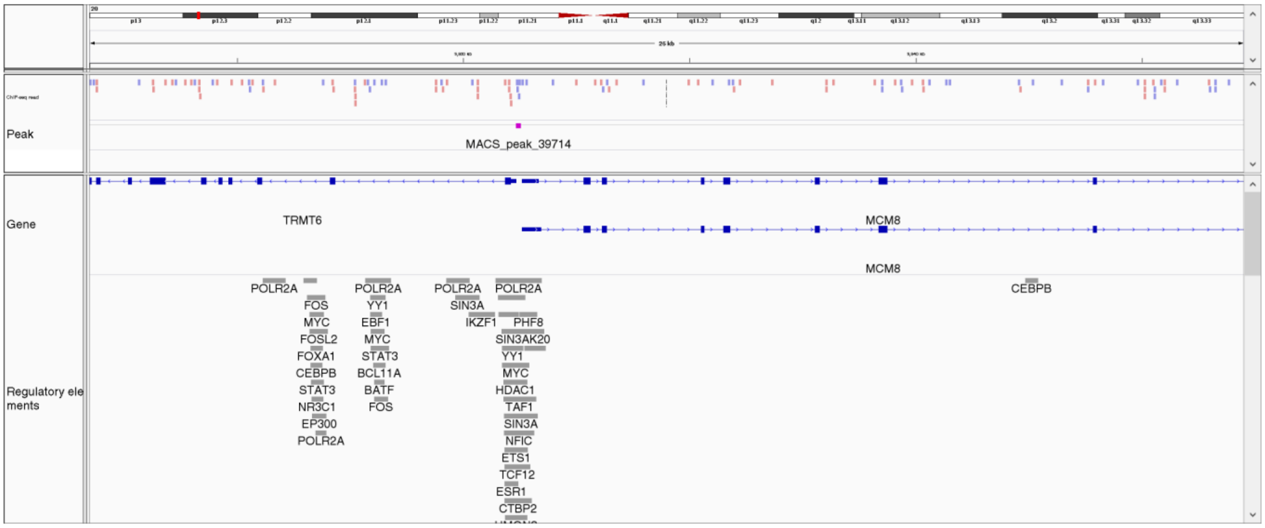

ODC1 (ornithine decarboxylase 1)

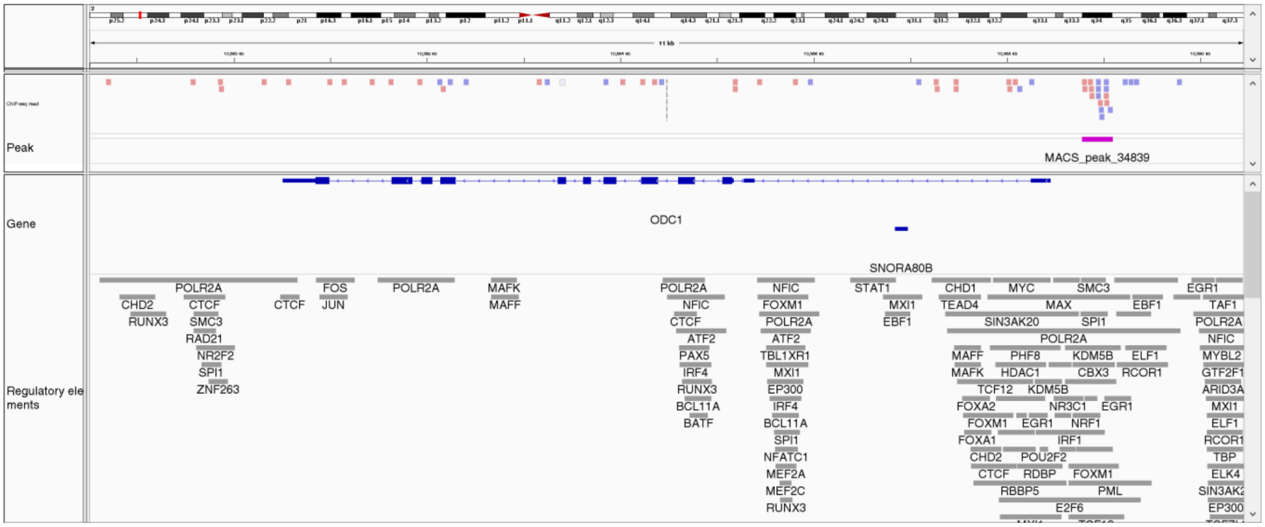

BIRC5 (baculoviral IAP repeat containing 5)

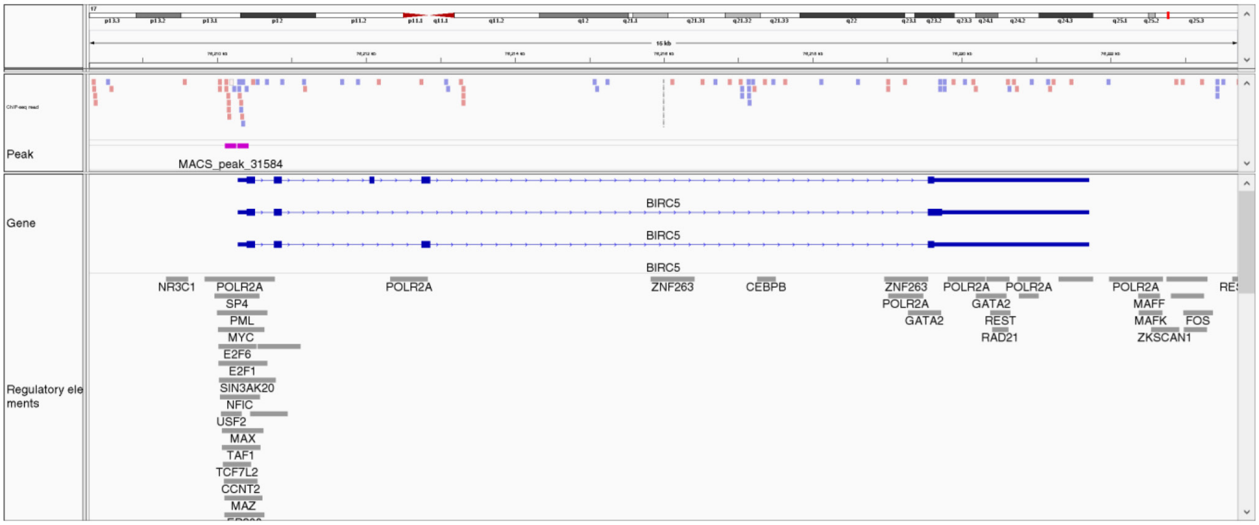

LUC7L (LUC7-like)

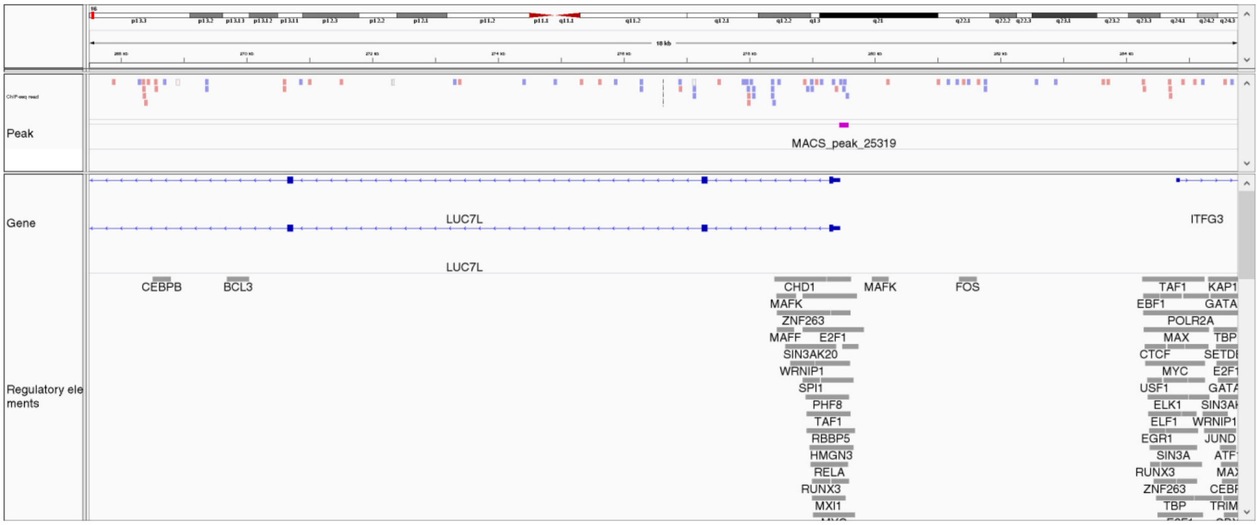

TWIST1 (twist family bHLH transcription factor 1)

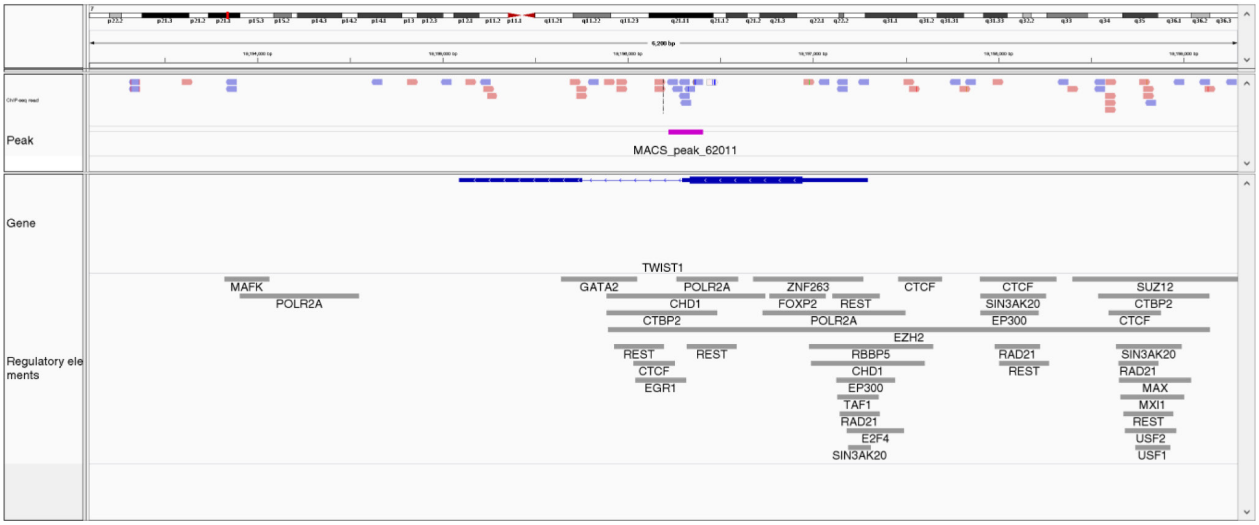

RAB5C (baculoviral IAP repeat containing 5)

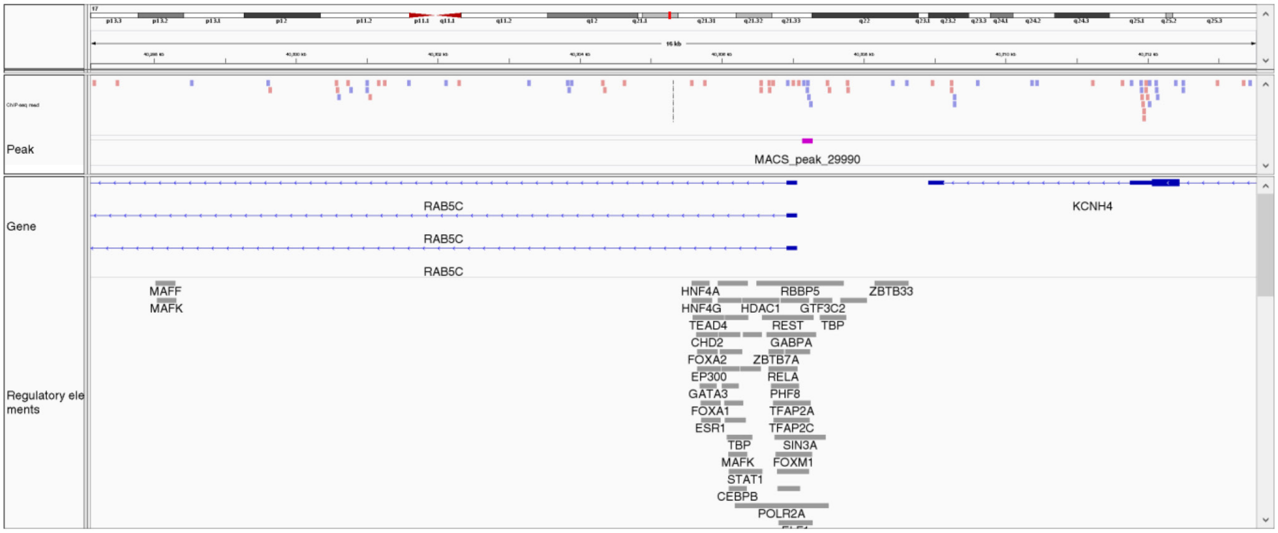

AURKA (aurora kinase A)

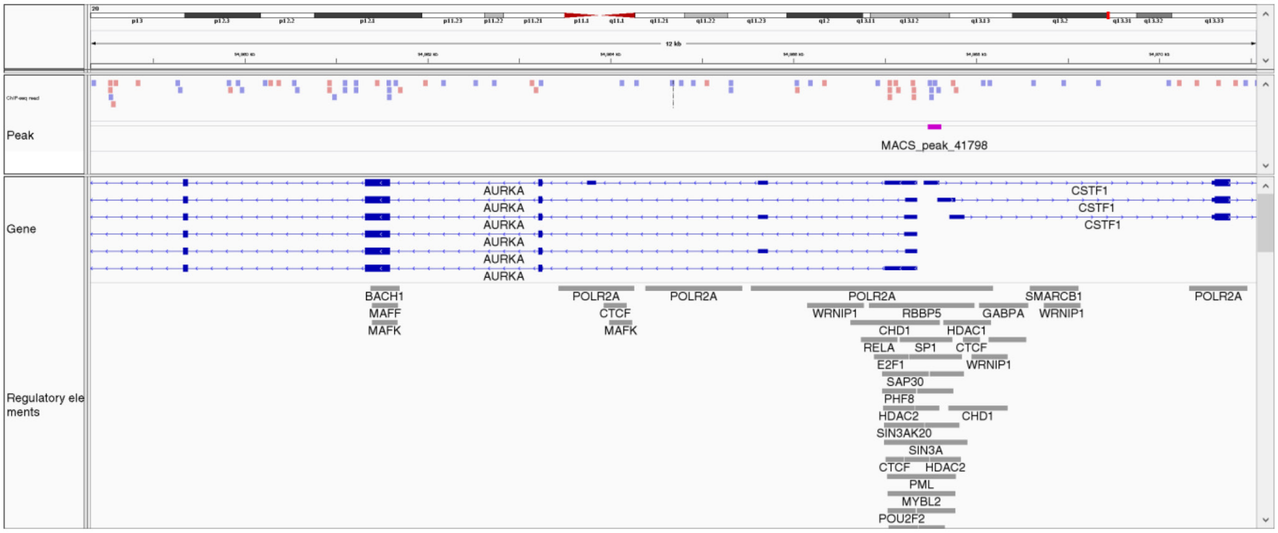

H1F0 (H1 histone family, member 0)

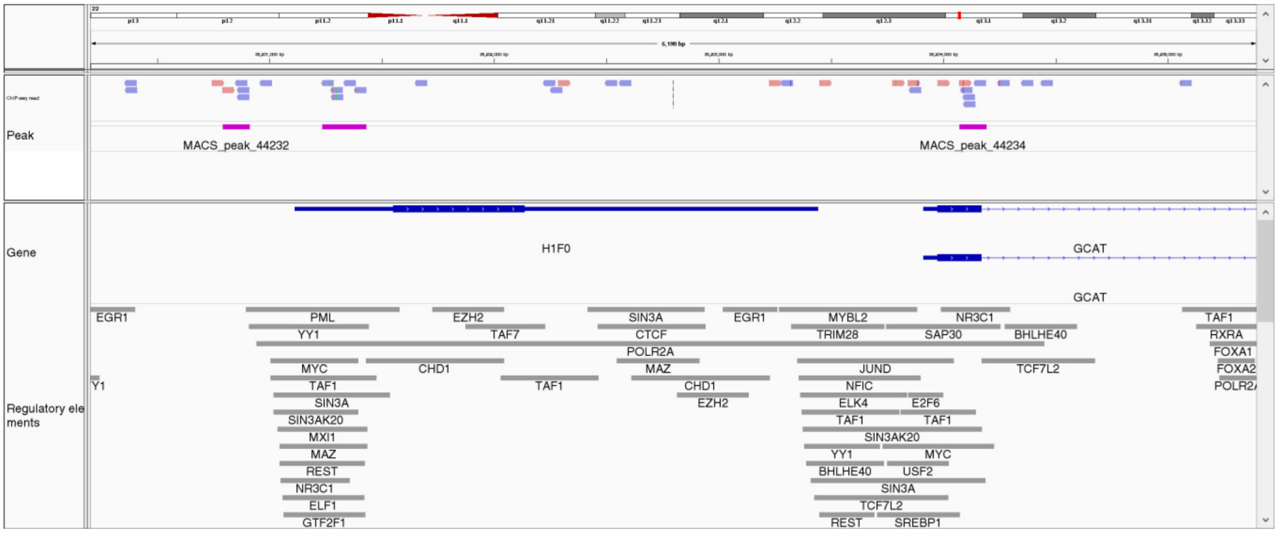

MYBL2 (MYB proto-oncogene like 2)

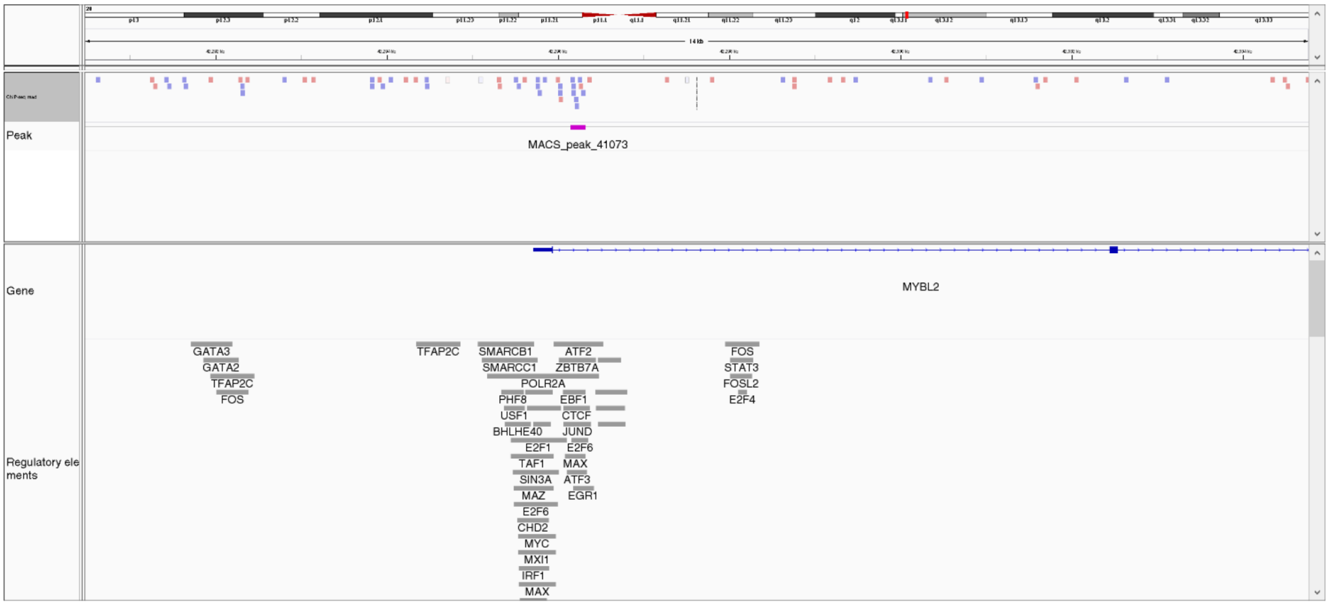

Supplementary Figure S2: Genomic mapping of ChIP-seq data reveals binding of MYCN on the promoters of known MYCN-bound genes. The ChIP-seq reads and peaks called by MACS were visualized by IGV (http://www.broadinstitute.org/igv/). The "regulatory elements" was obtained from UCSC genome browser (https://genome.ucsc.edu/).

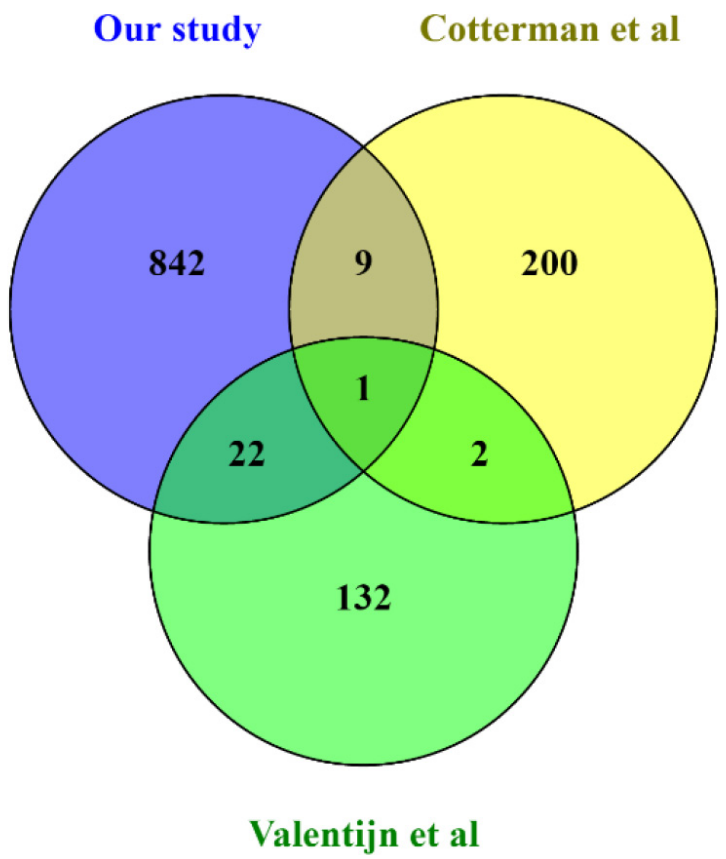

Supplementary Figure S3: The comparison of MYCN-regulated genes identified by our method and other studies. Cotterman et al. [2] and Valentijn et al. [3] identified 212 and 157 MYCN-regulated genes by integrating MYCN ChIP-chip and microarray experiments in IMR32 and TET21N cell lines, respectively.

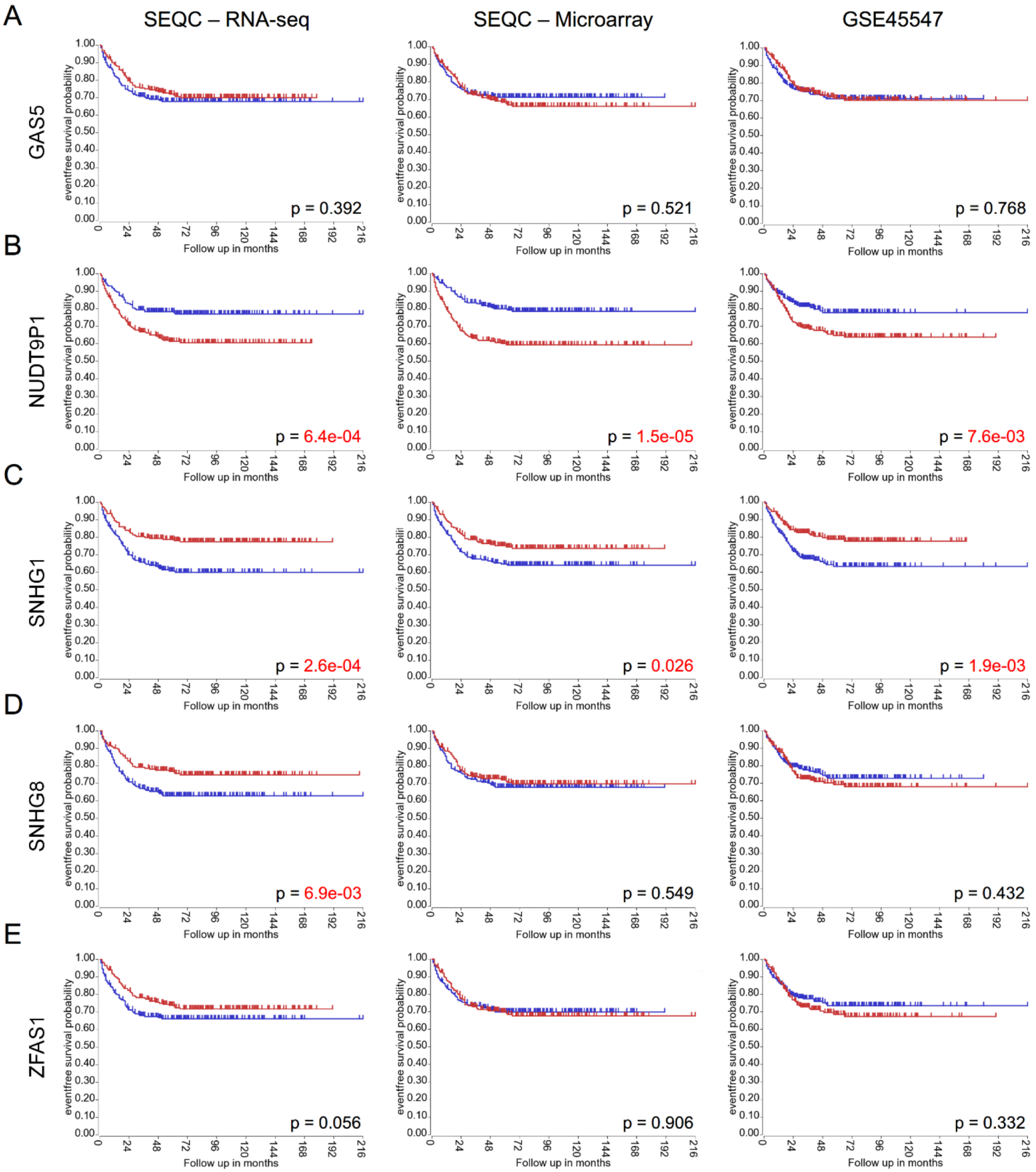

Supplementary Figure S4: Some of MYCN-regulated long non-coding RNAs (lncRNAs) as a prognostic marker for neuroblastoma. Kaplan-Meier curve indicating survival (in months) of patients with high (blue) and low (red) expression of five lncRNAs (GAS5 (**A**), NUTD9P1 (**B**), SNHG1 (**C**), SNHG8 (**D**), and ZFAS1 (**E**) based on median values in three different cohort data. The figures are generated by R2 bioinformatics tool (http://r2.amc.nl). The indicated p values were obtained from Log-rank test.

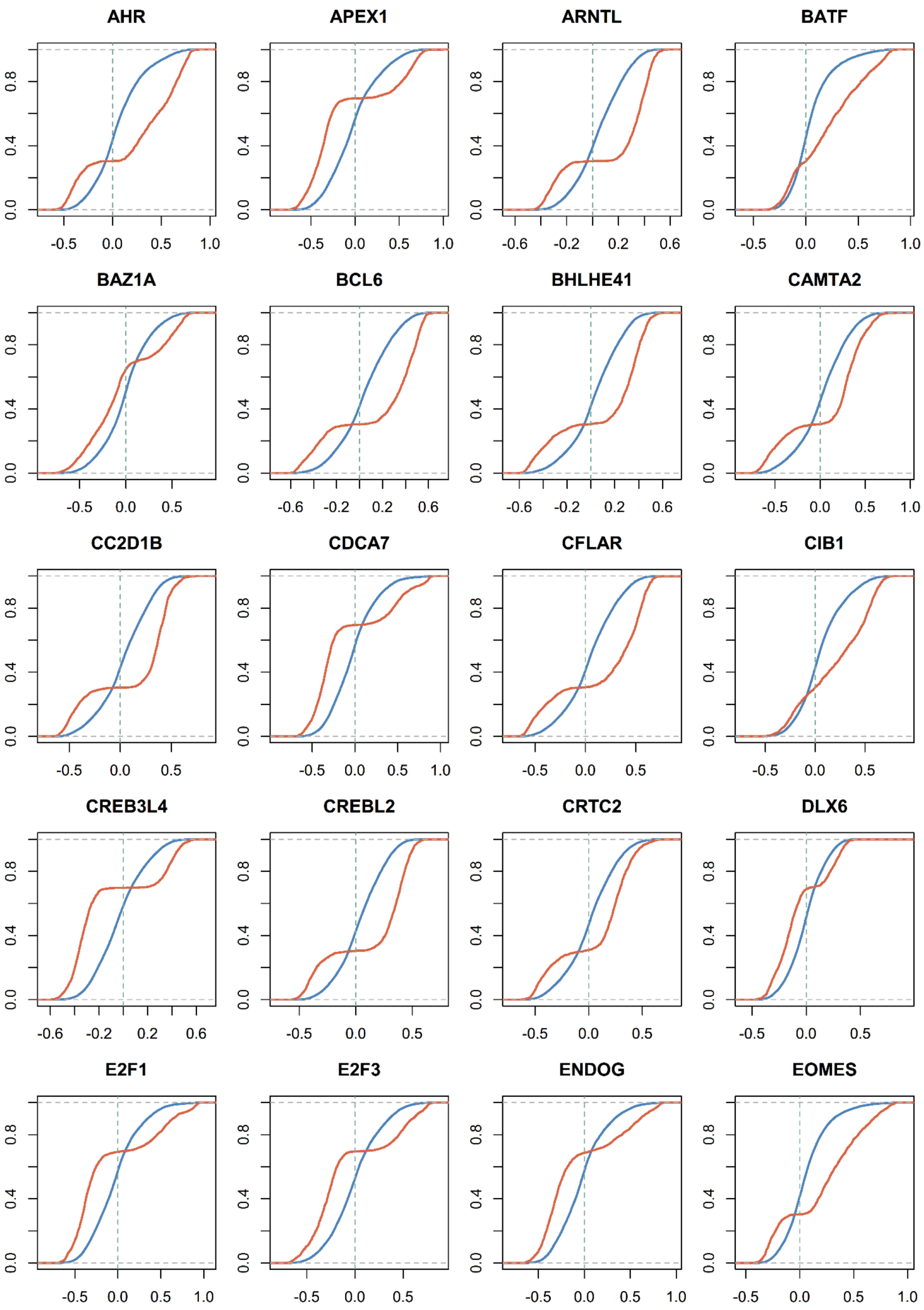
AHR
APEX1
ARNTL
BATF
BAZ1A
BCL6
BHLHE41
CAMTA2
CC2D1B
CDCA7
CFLAR
CIB1
CREB3L4
CREBL2
CRTC2
DLX6
E2F1
E2F3
ENDOG
EOMES
0.0
0.4
0.8
-0.5
0.0
0.5
1.0
-0.6
-0.2
0.2
0.6

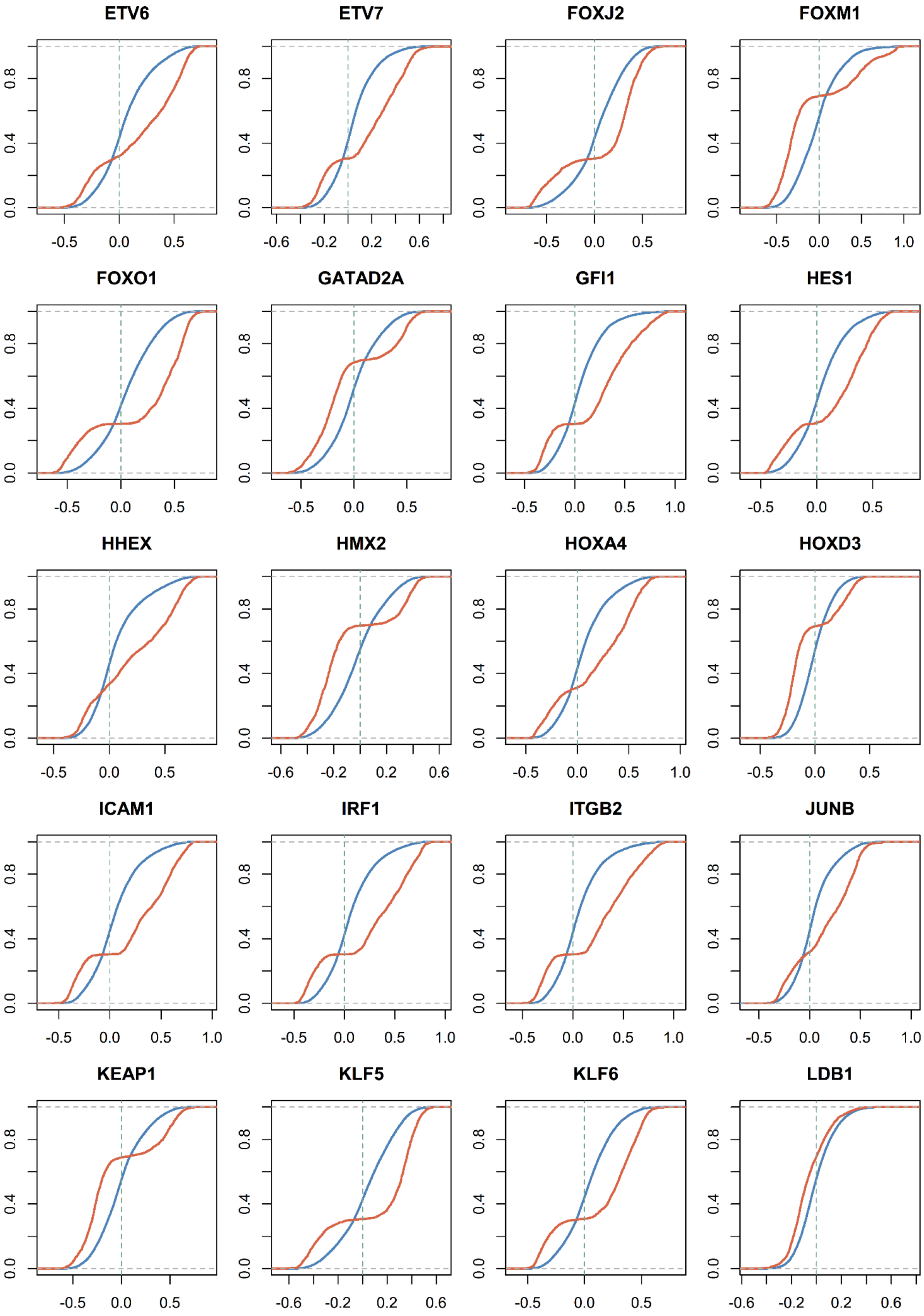
ETV6
0.0
0.4
0.8
-0.5
0.0
0.5
ETV7
0.0
0.4
0.8
-0.6
-0.2
0.2
0.6
FOXJ2
0.0
0.4
0.8
-0.5
0.0
0.5
FOXM1
0.0
0.4
0.8
-0.5
0.0
0.5
1.0
FOXO1
0.0
0.4
0.8
-0.5
0.0
0.5
GATAD2A
0.0
0.4
0.8
-0.5
0.0
0.5
GFI1
0.0
0.4
0.8
-0.5
0.0
0.5
1.0
HES1
0.0
0.4
0.8
-0.5
0.0
0.5
HHEX
0.0
0.4
0.8
-0.5
0.0
0.5
HMX2
0.0
0.4
0.8
-0.6
-0.2
0.2
0.6
HOXA4
0.0
0.4
0.8
-0.5
0.0
0.5
1.0
HOXD3
0.0
0.4
0.8
-0.5
0.0
0.5
ICAM1
0.0
0.4
0.8
-0.5
0.0
0.5
1.0
IRF1
0.0
0.4
0.8
-0.5
0.0
0.5
1.0
ITGB2
0.0
0.4
0.8
-0.5
0.0
0.5
1.0
JUNB
0.0
0.4
0.8
-0.5
0.0
0.5
1.0
KEAP1
0.0
0.4
0.8
-0.5
0.0
0.5
KLF5
0.0
0.4
0.8
-0.6
-0.2
0.2
0.6
KLF6
0.0
0.4
0.8
-0.5
0.0
0.5
LDB1
0.0
0.4
0.8
-0.6
-0.2
0.2
0.6

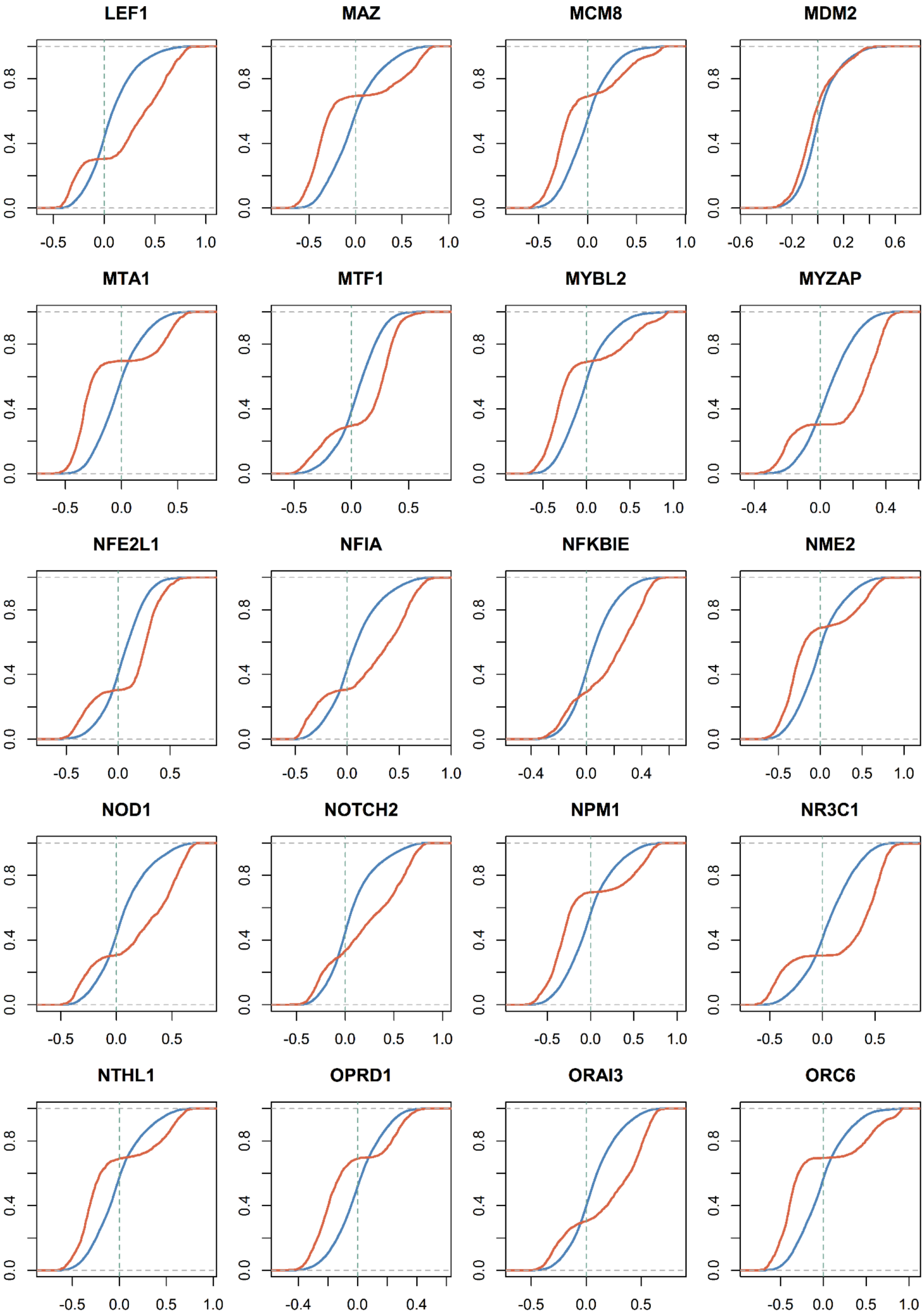

LEF1
MAZ
MCM8
MDM2
MTA1
MTF1
MYBL2
MYZAP
NFE2L1
NFIA
NFKBIE
NME2
NOD1
NOTCH2
NPM1
NR3C1
NTHL1
OPRD1
ORAI3
ORC6
0.0
0.4
0.8
-0.5
0.0
0.5
1.0
-0.6
-0.2
0.2
0.6
-0.4
0.4

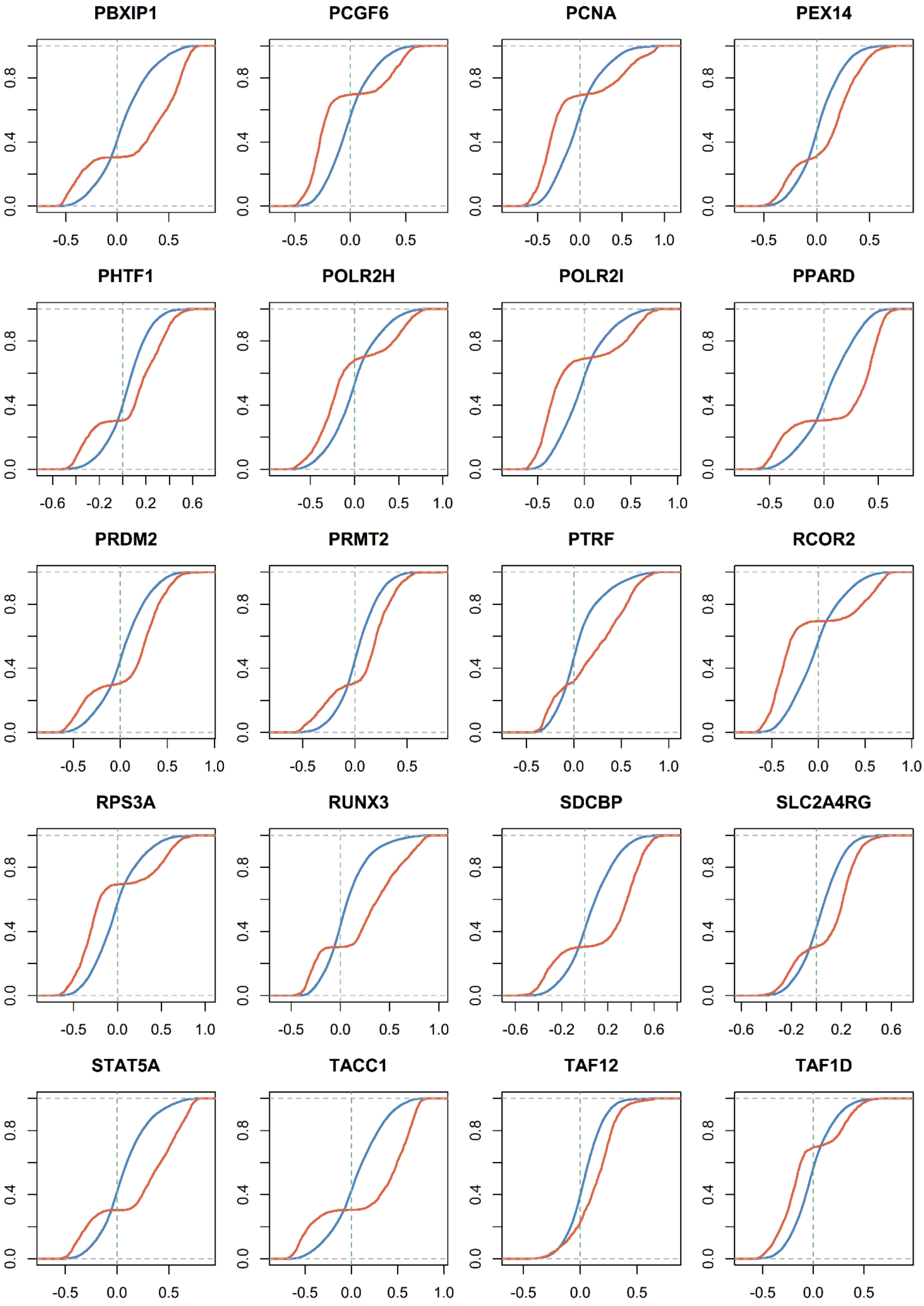
PBXIP1
PCGF6
PCNA
PEX14
PHTF1
POLR2H
POLR2I
PPARD
PRDM2
PRMT2
PTRF
RCOR2
RPS3A
RUNX3
SDCBP
SLC2A4RG
STAT5A
TACC1
TAF12
TAF1D

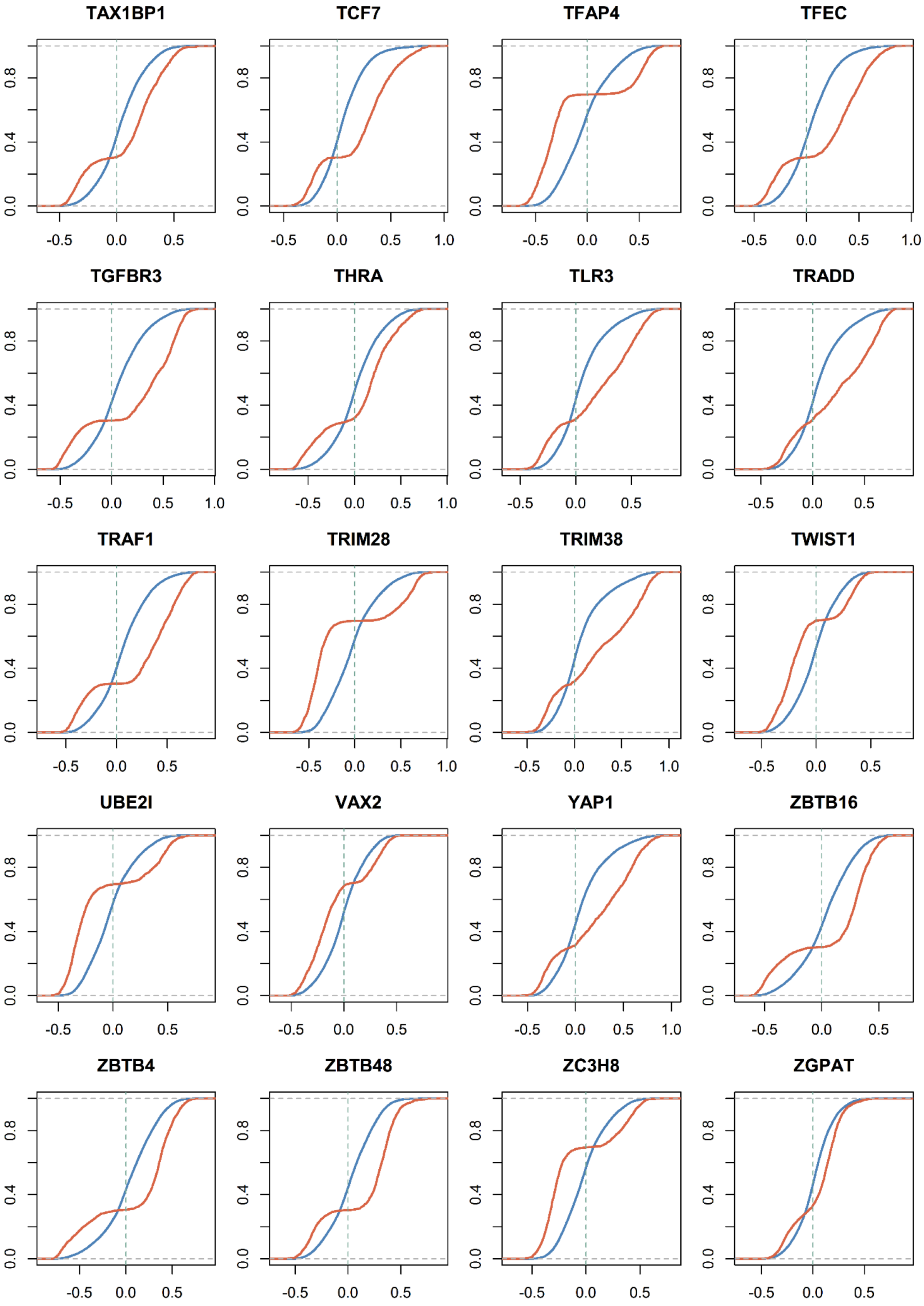
TAX1BP1
TCF7
TFAP4
TFEC
TGFBR3
THRA
TLR3
TRADD
TRAF1
TRIM28
TRIM38
TWIST1
UBE2I
VAX2
YAP1
ZBTB16
ZBTB4
ZBTB48
ZC3H8
ZGPAT
0.0
0.4
0.8
-0.5
0.0
0.5
1.0

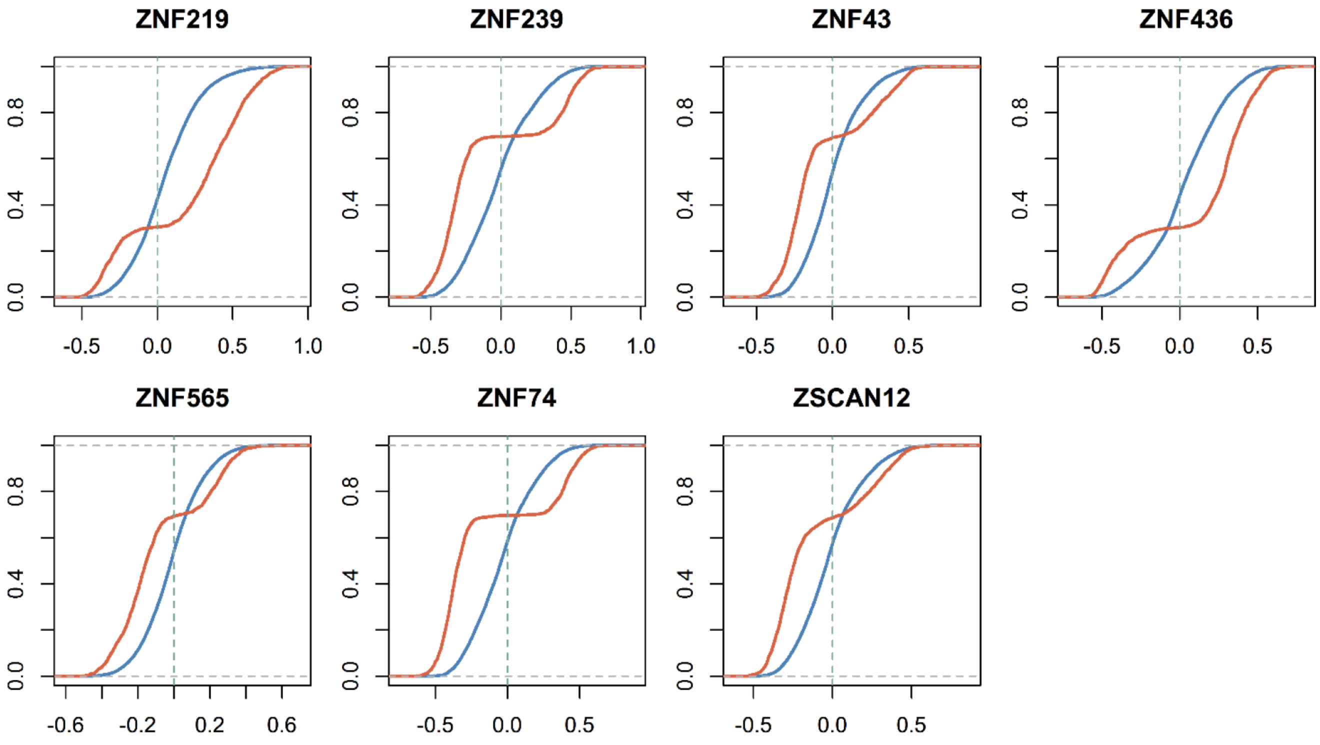

Supplementary Figure S5: Correlation analysis between MYCN-correlated genes and MYCN-regulated transcription factors. Red and green lines denote the correlation distribution of a given TF to MYCN-correlated genes and other genes, respectively. The x-axis is the Spearman correlation coefficient and the y-axis is the cumulative probability.

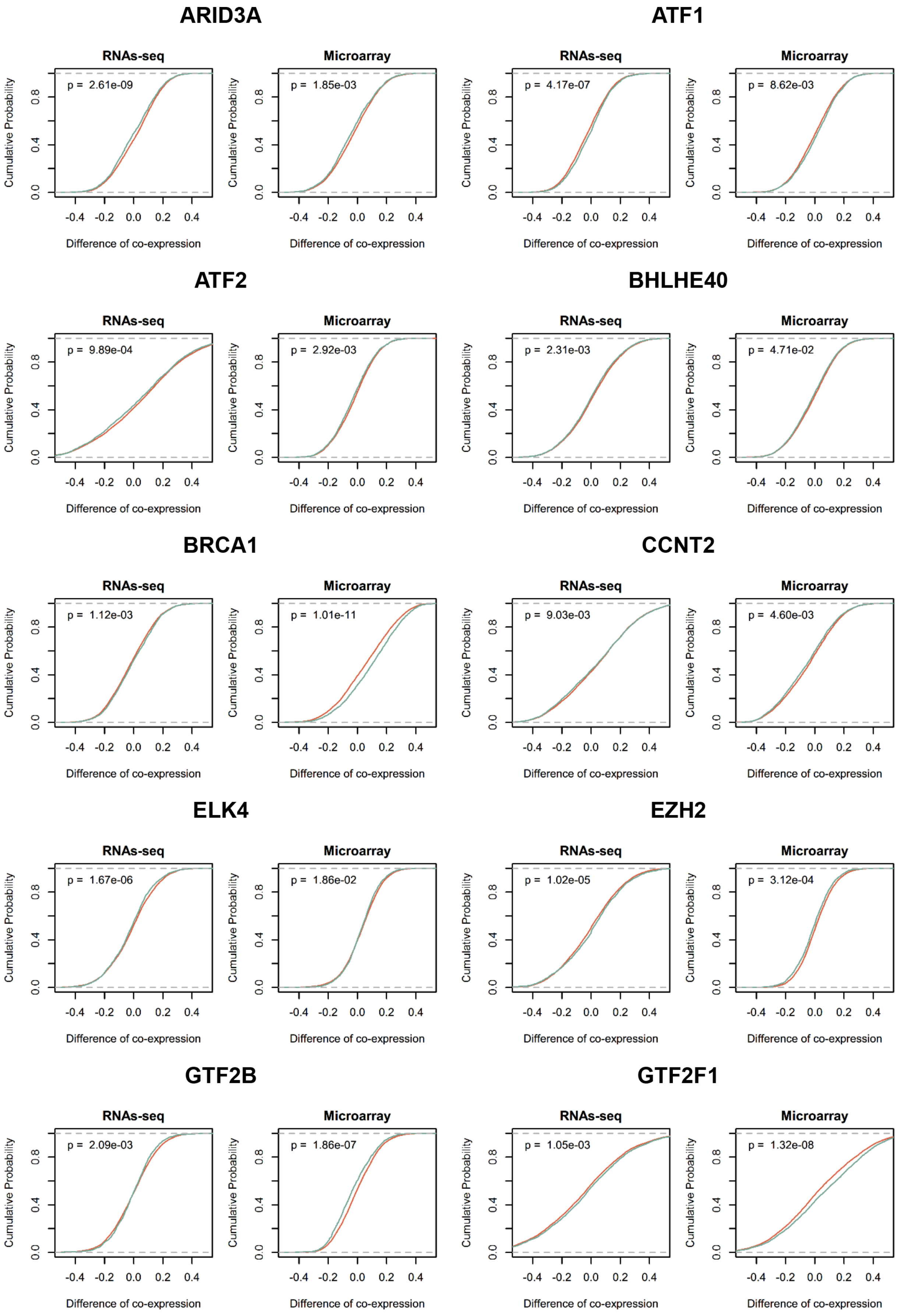

ARID3A
RNAs-seq
p = 2.61e-09
Microarray
p = 1.85e-03
ATF1
RNAs-seq
p = 4.17e-07
Microarray
p = 8.62e-03
ATF2
RNAs-seq
p = 9.89e-04
Microarray
p = 2.92e-03
BHLHE40
RNAs-seq
p = 2.31e-03
Microarray
p = 4.71e-02
BRCA1
RNAs-seq
p = 1.12e-03
Microarray
p = 1.01e-11
CCNT2
RNAs-seq
p = 9.03e-03
Microarray
p = 4.60e-03
ELK4
RNAs-seq
p = 1.67e-06
Microarray
p = 1.86e-02
EZH2
RNAs-seq
p = 1.02e-05
Microarray
p = 3.12e-04
GTF2B
RNAs-seq
p = 2.09e-03
Microarray
p = 1.86e-07
GTF2F1
RNAs-seq
p = 1.05e-03
Microarray
p = 1.32e-08
Cumulative Probability
0.0
0.4
0.8
-0.4
-0.2
0.0
0.2
0.4
Difference of co-expression

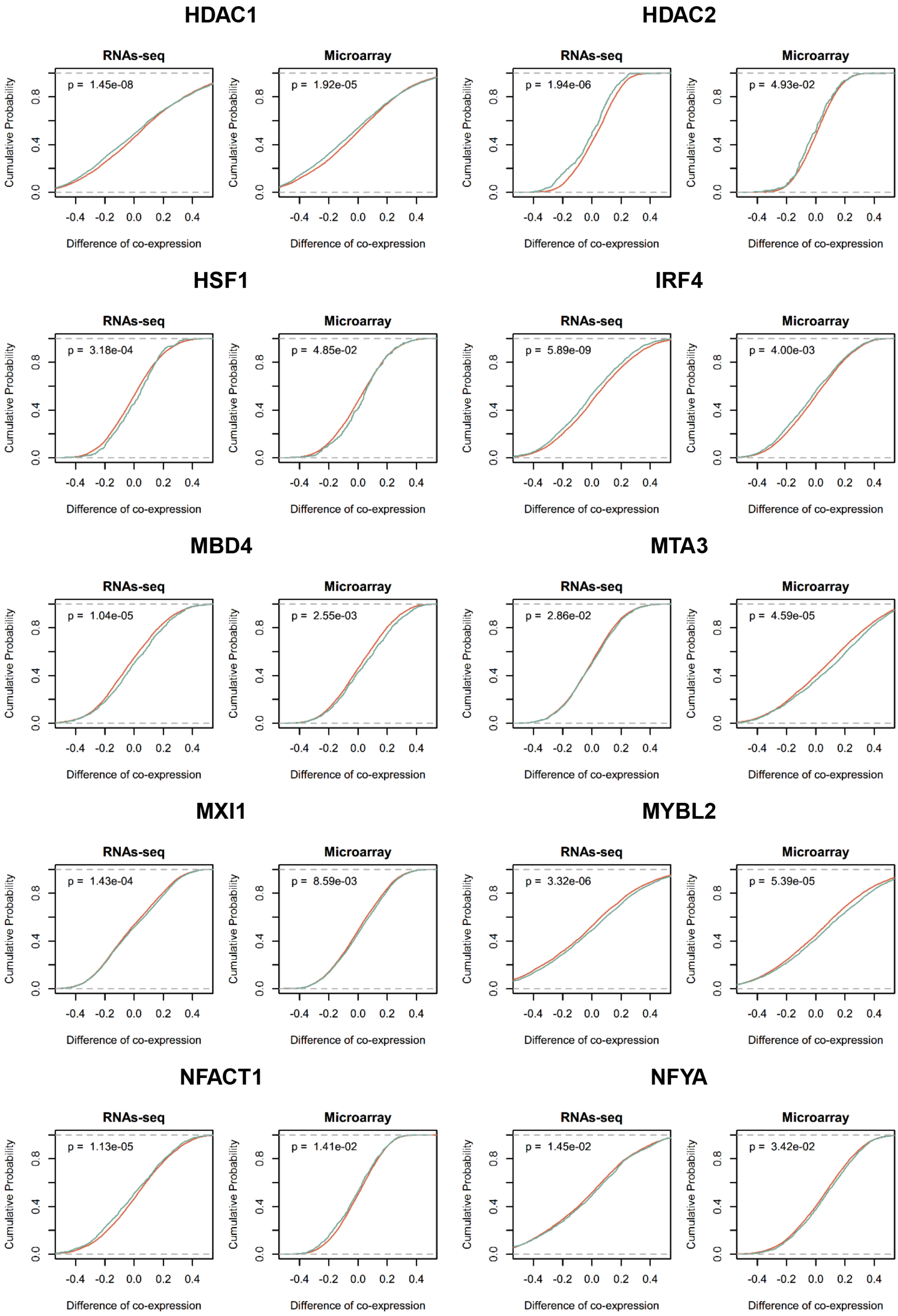

HDAC1
RNAs-seq
p = 1.45e-08
Microarray
p = 1.92e-05
HDAC2
RNAs-seq
p = 1.94e-06
Microarray
p = 4.93e-02
HSF1
RNAs-seq
p = 3.18e-04
Microarray
p = 4.85e-02
IRF4
RNAs-seq
p = 5.89e-09
Microarray
p = 4.00e-03
MBD4
RNAs-seq
p = 1.04e-05
Microarray
p = 2.55e-03
MTA3
RNAs-seq
p = 2.86e-02
Microarray
p = 4.59e-05
MXI1
RNAs-seq
p = 1.43e-04
Microarray
p = 8.59e-03
MYBL2
RNAs-seq
p = 3.32e-06
Microarray
p = 5.39e-05
NFACT1
RNAs-seq
p = 1.13e-05
Microarray
p = 1.41e-02
NFYA
RNAs-seq
p = 1.45e-02
Microarray
p = 3.42e-02
Cumulative Probability
Difference of co-expression

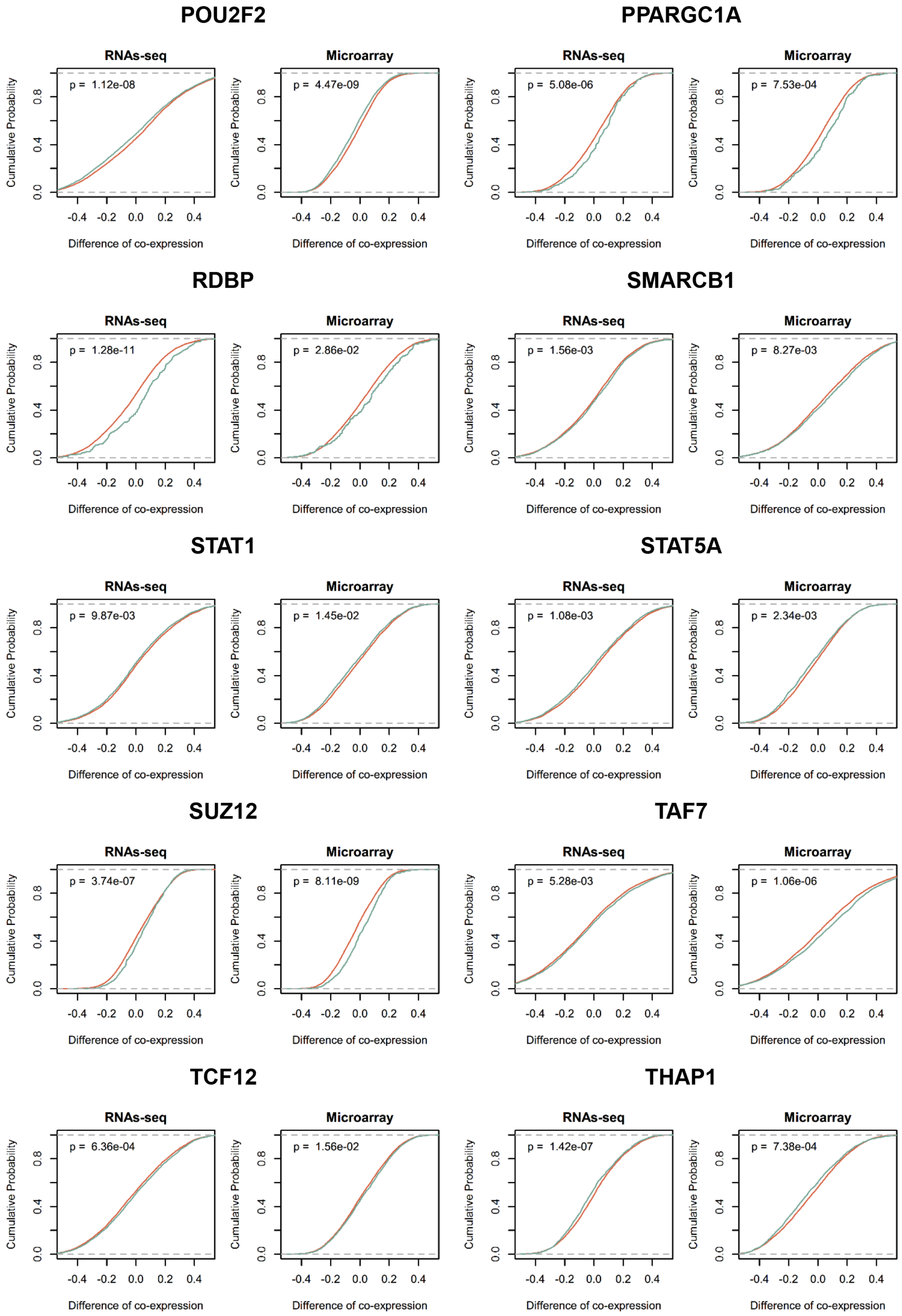
POU2F2
PPARGC1A
RDBP
SMARCB1
STAT1
STAT5A
SUZ12
TAF7
TCF12
THAP1
RNAs-seq
Microarray
Cumulative Probability
Difference of co-expression
p = 1.12e-08
p = 4.47e-09
p = 5.08e-06
p = 7.53e-04
p = 1.28e-11
p = 2.86e-02
p = 1.56e-03
p = 8.27e-03
p = 9.87e-03
p = 1.45e-02
p = 1.08e-03
p = 2.34e-03
p = 3.74e-07
p = 8.11e-09
p = 5.28e-03
p = 1.06e-06
p = 6.36e-04
p = 1.56e-02
p = 1.42e-07
p = 7.38e-04

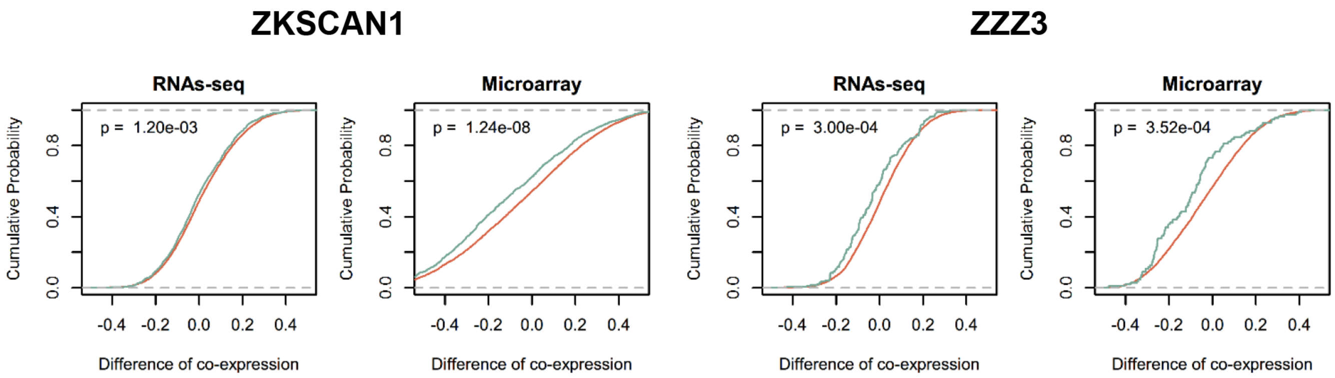

Supplementary Figure S6: Differential coexpression of MYCN and other regulators to their co-targets. Red and green lines denote the difference coexpression between MYCN and genes bound by MYCN as well as both MYCN and the given regulator, respectively. The *p* values are obtained from Kolmogorov–Smirnov test.

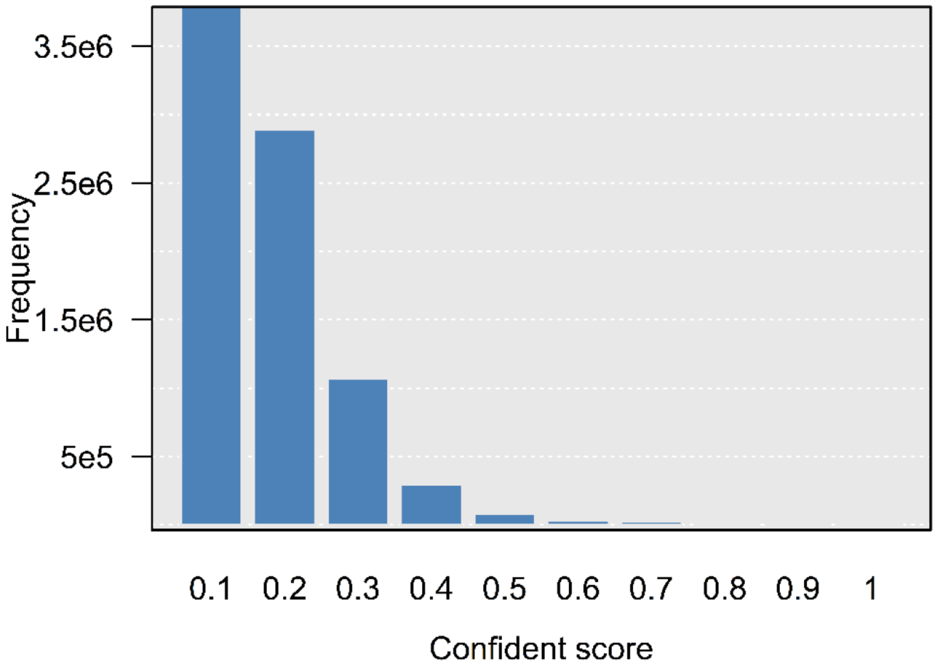

Supplementary Figure S7: Distribution of confident score for miRNA-target relations.

REFERENCES

1. Murphy DM, Buckley PG, Bryan K, Das S, Alcock L, Foley NH, Prenter S, Bray I, Watters KM, Higgins D, Stallings RL. Global MYCN transcription factor binding analysis in neuroblastoma reveals association with distinct E-box motifs and regions of DNA hypermethylation. PLoS One. 2009; 4:e8154.
2. Cotterman R, Jin VX, Krig SR, Lemen JM, Wey A, Farnham PJ, Knoepfler PS. N-Myc regulates a widespread euchromatic program in the human genome partially independent of its role as a classical transcription factor. Cancer Res. 2008; 68:9654–9662.
3. Valentijn LJ, Koster J, Haneveld F, Aissa RA, van Sluis P, Broekmans ME, Molenaar JJ, van Nes J, Versteeg R. Functional MYCN signature predicts outcome of neuroblastoma irrespective of MYCN amplification. Proc Natl Acad Sci U S A. 2012; 109:19190–19195.
4. Lodrini M, Oehme I, Schroeder C, Milde T, Schier MC, Kopp-Schneider A, Schulte JH, Fischer M, De Preter K, Pattyn F, Castoldi M, Muckenthaler MU, Kulozik AE, et al. MYCN and HDAC2 cooperate to repress miR-183 signaling in neuroblastoma. Nucleic Acids Res. 2013; 41:6018–6033.
5. Shohet JM, Ghosh R, Coarfa C, Ludwig A, Benham AL, Chen Z, Patterson DM, Barbieri E, Mestdagh P, Sikorski DN, Milosavljevic A, Kim ES, Gunaratne PH. A genome-wide search for promoters that respond to increased MYCN reveals both new oncogenic and tumor suppressor microRNAs associated with aggressive neuroblastoma. Cancer Res. 2011; 71:3841–3851.

Supplementary Table S1: Genome-wide identification of binding sites for MYCN in neuroblastoma

Cell line	Platform	Reference
Kelly, SK-N-AS, and TET21N	ChIP-chip	[1]
TET21N	ChIP-chip	[2]
IMR32	ChIP-chip	[3]
Kelly	ChIP-seq	[4]
MYCN-3	ChIP-seq	[5]

Supplementary Table S2: MYCN-correlated genes and their correlations with other transcription factors

Supplementary Table S3: List of MYCN-regulated genes

GeneID	Symbol	Regulation direction by MYCN	Gene Information				miRNA regulation		
			Gene type	TF	Kinase	Phosphotase	Number of MYCN-regulated miRNA targets	Ajusted *p* value	List of targets of MYCN-regulated miRNAs (delimited by "|")
10347	ABCA7	repression	protein-coding				0	1.00E+00	
8714	ABCC3	repression	protein-coding				0	1.00E+00	
222166	MTURN	repression	protein-coding				10	6.87E-07	hsa-miR-330-3p|hsa-miR-24-3p|hsa-miR-93-5p|hsa-miR-7-5p|hsa-miR-181d-5p|hsa-miR-181a-5p|hsa-miR-181b-5p|hsa-miR-124-3p|hsa-miR-27b-3p|hsa-miR-27a-3p
84448	ABLIM2	repression	protein-coding				1	2.48E-01	hsa-miR-24-3p
37	ACADVL	repression	protein-coding				1	5.33E-01	hsa-miR-124-3p
9744	ACAP1	repression	protein-coding				0	1.00E+00	
84320	ACBD6	activation	protein-coding				0	1.00E+00	
80221	ACSF2	repression	protein-coding				1	7.56E-02	hsa-miR-24-3p
51703	ACSL5	repression	protein-coding				0	1.00E+00	
23305	ACSL6	repression	protein-coding				2	4.00E-01	hsa-miR-24-3p|hsa-miR-7-5p
79611	ACSS3	repression	protein-coding				1	2.10E-01	hsa-miR-124-3p
119	ADD2	activation	protein-coding				4	8.03E-02	hsa-miR-33a-5p|hsa-miR-24-3p|hsa-miR-7-5p|hsa-miR-760
57211	ADGRG6	repression	protein-coding				0	1.00E+00	
54936	ADPRHL2	repression	protein-coding				0	1.00E+00	
84830	ADTRP	repression	protein-coding				0	1.00E+00	
2059	EPS8	repression	protein-coding				9	4.70E-06	hsa-miR-330-3p|hsa-miR-25-3p|hsa-miR-181d-5p|hsa-miR-181a-5p|hsa-miR-181b-5p|hsa-miR-124-3p|hsa-miR-27a-3p|hsa-miR-345-5p|hsa-miR-27b-3p
191	AHCY	activation	protein-coding				0	1.00E+00	
196	AHR	repression	protein-coding	Yes			2	1.00E+00	hsa-miR-124-3p|hsa-miR-7-5p
202	AIM1	repression	protein-coding				0	1.00E+00	
7965	AIMP2	activation	protein-coding				0	1.00E+00	
79647	AKIRIN1	repression	protein-coding				5	5.30E-02	hsa-miR-181d-5p|hsa-miR-27b-3p|hsa-miR-27a-3p|hsa-miR-181a-5p|hsa-miR-181b-5p
5832	ALDH18A1	activation	protein-coding				0	1.00E+00	
230	ALDOC	repression	protein-coding				1	4.68E-01	hsa-miR-7-5p
10195	ALG3	activation	protein-coding				0	1.00E+00	
262	AMD1	activation	protein-coding				4	6.58E-02	hsa-miR-2110|hsa-miR-7-5p|hsa-miR-27a-3p|hsa-miR-27b-3p
54522	ANKRD16	activation	protein-coding				0	1.00E+00	

148741	ANKRD35	repression	protein-coding				0	1.00E+00	
10541	ANP32B	activation	protein-coding			Yes	3	7.30E-02	hsa-miR-330-3p|hsa-miR-27a-3p|hsa-miR-27b-3p
301	ANXA1	repression	protein-coding				1	3.76E-01	hsa-miR-2110
328	APEX1	activation	protein-coding	Yes			0	1.00E+00	
57136	APMAP	repression	protein-coding				0	1.00E+00	
1316	KLF6	repression	protein-coding	Yes			9	4.70E-06	hsa-miR-25-3p|hsa-miR-181a-5p|hsa-miR-24-3p|hsa-miR-7-5p|hsa-miR-181d-5p|hsa-miR-181b-5p|hsa-miR-124-3p|hsa-miR-27a-3p|hsa-miR-27b-3p
5594	MAPK1	repression	protein-coding		Yes		13	4.70E-06	hsa-miR-2110|hsa-miR-181d-5p|hsa-miR-124-3p|hsa-miR-24-3p|hsa-miR-93-5p|hsa-miR-320a|hsa-miR-27b-3p|hsa-miR-330-3p|hsa-miR-27a-3p|hsa-miR-7-5p|hsa-miR-181a-5p|hsa-miR-181b-5p|hsa-miR-345-5p
395	ARHGAP6	repression	protein-coding				3	1.63E-01	hsa-miR-330-3p|hsa-miR-320a|hsa-miR-345-5p
64333	ARHGAP9	repression	protein-coding				0	1.00E+00	
84722	PSRC1	activation	protein-coding				6	3.02E-05	hsa-miR-7-5p|hsa-miR-181d-5p|hsa-miR-181a-5p|hsa-miR-181b-5p|hsa-miR-27a-3p|hsa-miR-27b-3p
10095	ARPC1B	repression	protein-coding				1	2.10E-01	hsa-miR-124-3p
10092	ARPC5	repression	protein-coding				4	1.38E-01	hsa-miR-124-3p|hsa-miR-93-5p|hsa-miR-320a|hsa-miR-330-3p
27106	ARRDC2	repression	protein-coding				0	1.00E+00	
55723	ASF1B	activation	protein-coding				1	6.28E-01	hsa-miR-24-3p
443	ASPA	repression	protein-coding				1	2.84E-01	hsa-miR-124-3p
79915	ATAD5	activation	protein-coding				0	1.00E+00	
84971	ATG4D	activation	protein-coding				0	1.00E+00	
471	ATIC	activation	protein-coding				0	1.00E+00	
521	ATP5I	activation	protein-coding				0	1.00E+00	
10159	ATP6AP2	repression	protein-coding				0	1.00E+00	
535	ATP6V0A1	repression	protein-coding				0	1.00E+00	
533	ATP6V0B	repression	protein-coding				0	1.00E+00	
9114	ATP6V0D1	repression	protein-coding				0	1.00E+00	
56674	TMEM9B	repression	protein-coding				8	3.02E-05	hsa-miR-25-3p|hsa-miR-33a-5p|hsa-miR-181d-5p|hsa-miR-181a-5p|hsa-miR-181b-5p|hsa-miR-27b-3p|hsa-miR-27a-3p|hsa-miR-320a

6790	AURKA	activation	protein-coding		Yes		2	1.71E-01	hsa-miR-25-3p|hsa-miR-124-3p
8705	B3GALT4	repression	protein-coding				0	1.00E+00	
10678	B3GNT2	repression	protein-coding				1	9.60E-01	hsa-miR-124-3p
8938	BAIAP3	repression	protein-coding				0	1.00E+00	
10538	BATF	repression	protein-coding	Yes			0	1.00E+00	
11177	BAZ1A	activation	protein-coding	Yes			2	4.89E-01	hsa-miR-7-5p|hsa-miR-320a
27241	BBS9	repression	protein-coding				0	1.00E+00	
8412	BCAR3	repression	protein-coding				0	1.00E+00	
593	BCKDHA	activation	protein-coding				1	1.72E-01	hsa-miR-124-3p
9802	DAZAP2	repression	protein-coding				9	1.07E-04	hsa-miR-181d-5p|hsa-miR-124-3p|hsa-miR-7-5p|hsa-miR-93-5p|hsa-miR-27b-3p|hsa-miR-27a-3p|hsa-miR-24-3p|hsa-miR-181a-5p|hsa-miR-181b-5p
8678	BECN1	repression	protein-coding				1	8.89E-01	hsa-miR-93-5p
79365	BHLHE41	repression	protein-coding	Yes			3	3.46E-01	hsa-miR-93-5p|hsa-miR-27b-3p|hsa-miR-27a-3p
23299	BICD2	repression	protein-coding				7	5.75E-02	hsa-miR-124-3p|hsa-miR-24-3p|hsa-miR-93-5p|hsa-miR-320a|hsa-miR-330-3p|hsa-miR-25-3p|hsa-miR-27a-3p
637	BID	activation	protein-coding				1	5.11E-01	hsa-miR-124-3p
332	BIRC5	activation	protein-coding				0	1.00E+00	
22884	WDR37	repression	protein-coding				10	3.44E-04	hsa-miR-760|hsa-miR-181d-5p|hsa-miR-24-3p|hsa-miR-93-5p|hsa-miR-27b-3p|hsa-miR-330-3p|hsa-miR-25-3p|hsa-miR-27a-3p|hsa-miR-181a-5p|hsa-miR-181b-5p
91272	BOD1	activation	protein-coding				0	1.00E+00	
23246	BOP1	activation	protein-coding				0	1.00E+00	
54836	BSPRY	repression	protein-coding				0	1.00E+00	
55643	BTBD2	activation	protein-coding				1	2.10E-01	hsa-miR-33a-5p
28969	BZW2	activation	protein-coding				1	9.72E-01	hsa-miR-320a
83938	C10orf11	repression	protein-coding				0	1.00E+00	
79703	C11orf80	repression	protein-coding				0	1.00E+00	
60686	C14orf93	activation	protein-coding				0	1.00E+00	
84326	C16orf13	activation	protein-coding				0	1.00E+00	
80178	C16orf59	activation	protein-coding				1	2.10E-01	hsa-miR-24-3p
90835	C16orf93	repression	protein-coding				0	1.00E+00	
55337	C19orf66	repression	protein-coding				0	1.00E+00	
55150	C19orf73	activation	protein-coding				0	1.00E+00	
127687	C1orf122	repression	protein-coding				0	1.00E+00	
54964	C1orf56	repression	protein-coding				1	3.76E-01	hsa-miR-93-5p
715	C1R	repression	protein-coding				0	1.00E+00	

154313	C6orf165	repression	protein-coding				0	1.00E+00	
93589	CACNA2D4	repression	protein-coding				0	1.00E+00	
790	CAD	activation	protein-coding				0	1.00E+00	
163688	CALML6	repression	protein-coding				0	1.00E+00	
50632	CALY	repression	protein-coding				0	1.00E+00	
23261	CAMTA1	repression	protein-coding				7	5.09E-02	hsa-miR-181d-5p|hsa-miR-124-3p|hsa-miR-93-5p|hsa-miR-320a|hsa-miR-25-3p|hsa-miR-181a-5p|hsa-miR-181b-5p
84910	TMEM87B	repression	protein-coding				6	3.48E-04	hsa-miR-181d-5p|hsa-miR-181a-5p|hsa-miR-181b-5p|hsa-miR-124-3p|hsa-miR-27a-3p|hsa-miR-27b-3p
10487	CAP1	repression	protein-coding				0	1.00E+00	
84698	CAPS2	repression	protein-coding				1	4.00E-01	hsa-miR-93-5p
29775	CARD10	repression	protein-coding				1	5.33E-01	hsa-miR-24-3p
831	CAST	repression	protein-coding				1	9.48E-01	hsa-miR-124-3p
875	CBS	activation	protein-coding				0	1.00E+00	
200014	CC2D1B	repression	protein-coding	Yes			2	3.23E-01	hsa-miR-24-3p|hsa-miR-124-3p
115098	CCDC124	activation	protein-coding				0	1.00E+00	
90693	CCDC126	repression	protein-coding				0	1.00E+00	
57639	CCDC146	repression	protein-coding				0	1.00E+00	
126075	CCDC159	repression	protein-coding				0	1.00E+00	
149473	CCDC24	repression	protein-coding				0	1.00E+00	
1910	EDNRB	repression	protein-coding				8	5.81E-04	hsa-miR-33a-5p|hsa-miR-181d-5p|hsa-miR-124-3p|hsa-miR-27b-3p|hsa-miR-25-3p|hsa-miR-27a-3p|hsa-miR-181a-5p|hsa-miR-181b-5p
890	CCNA2	activation	protein-coding				2	3.50E-01	hsa-miR-27a-3p|hsa-miR-27b-3p
10161	LPAR6	repression	protein-coding				3	5.97E-04	hsa-miR-27a-3p|hsa-miR-24-3p|hsa-miR-27b-3p
3609	ILF3	activation	protein-coding				7	1.09E-03	hsa-miR-25-3p|hsa-miR-24-3p|hsa-miR-181b-5p|hsa-miR-7-5p|hsa-miR-181d-5p|hsa-miR-181a-5p|hsa-miR-320a
9236	CCPG1	repression	protein-coding				1	1.00E+00	hsa-miR-320a
1236	CCR7	repression	protein-coding				1	7.92E-01	hsa-miR-320a
27342	RABGEF1	repression	protein-coding				6	1.19E-03	hsa-miR-33a-5p|hsa-miR-7-5p|hsa-miR-181d-5p|hsa-miR-181a-5p|hsa-miR-181b-5p|hsa-miR-124-3p
8763	CD164	repression	protein-coding				3	5.12E-01	hsa-miR-124-3p|hsa-miR-24-3p|hsa-miR-93-5p
51293	CD320	activation	protein-coding				0	1.00E+00	

969	CD69	repression	protein-coding					3	1.97E-01	hsa-miR-25-3p|hsa-miR-7-5p|hsa-miR-93-5p
9308	CD83	repression	protein-coding					0	1.00E+00	
925	CD8A	repression	protein-coding					0	1.00E+00	
928	CD9	repression	protein-coding					0	1.00E+00	
56990	CDC42SE2	repression	protein-coding					2	6.90E-01	hsa-miR-27a-3p|hsa-miR-345-5p
83879	CDCA7	activation	protein-coding	Yes				3	1.05E-01	hsa-miR-93-5p|hsa-miR-124-3p|hsa-miR-320a
984	CDK11B	repression	protein-coding		Yes			0	1.00E+00	
81620	CDT1	activation	protein-coding					0	1.00E+00	
9620	CELSR1	repression	protein-coding					1	8.78E-01	hsa-miR-124-3p
1062	CENPE	activation	protein-coding					0	1.00E+00	
79019	CENPM	activation	protein-coding					0	1.00E+00	
55839	CENPN	activation	protein-coding					0	1.00E+00	
79682	CENPU	activation	protein-coding					0	1.00E+00	
9731	CEP104	repression	protein-coding					0	1.00E+00	
80217	CFAP43	repression	protein-coding					0	1.00E+00	
8837	CFLAR	repression	protein-coding	Yes				0	1.00E+00	
84952	CGNL1	repression	protein-coding					0	1.00E+00	
10669	CGREF1	activation	protein-coding					0	1.00E+00	
11261	CHP1	repression	protein-coding			Yes		2	4.42E-01	hsa-miR-7-5p|hsa-miR-124-3p
10519	CIB1	repression	protein-coding	Yes				0	1.00E+00	
160365	CLECL1	repression	protein-coding					0	1.00E+00	
9022	CLIC3	repression	protein-coding					0	1.00E+00	
11276	SYNRG	repression	protein-coding					6	1.19E-03	hsa-miR-93-5p|hsa-miR-181d-5p|hsa-miR-181a-5p|hsa-miR-181b-5p|hsa-miR-27b-3p|hsa-miR-27a-3p
54982	CLN6	activation	protein-coding					0	1.00E+00	
9746	CLSTN3	repression	protein-coding					0	1.00E+00	
1213	CLTC	repression	protein-coding					2	8.78E-01	hsa-miR-2110|hsa-miR-24-3p
84319	CMSS1	activation	protein-coding					0	1.00E+00	
26507	CNNM1	activation	protein-coding					2	4.54E-01	hsa-miR-25-3p|hsa-miR-33a-5p
1690	COCH	activation	protein-coding					0	1.00E+00	
10087	COL4A3BP	repression	protein-coding					3	1.21E-01	hsa-miR-25-3p|hsa-miR-7-5p|hsa-miR-124-3p
1286	COL4A4	repression	protein-coding					0	1.00E+00	
10391	CORO2B	repression	protein-coding					2	5.26E-01	hsa-miR-2110|hsa-miR-93-5p
9377	COX5A	activation	protein-coding					0	1.00E+00	
10815	CPLX1	activation	protein-coding					0	1.00E+00	
594855	CPLX3	activation	protein-coding					1	7.56E-02	hsa-miR-24-3p
57699	CPNE5	repression	protein-coding					1	2.84E-01	hsa-miR-24-3p
51692	CPSF3	activation	protein-coding					0	1.00E+00	
54504	CPVL	repression	protein-coding					0	1.00E+00	

119587	CPXM2	repression	protein-coding					0	1.00E+00	
1382	CRABP2	activation	protein-coding					0	1.00E+00	
148327	CREB3L4	activation	protein-coding	Yes				0	1.00E+00	
7529	YWHAB	repression	protein-coding					7	1.19E-03	hsa-miR-330-3p|hsa-miR-27a-3p|hsa-miR-7-5p|hsa-miR-181a-5p|hsa-miR-181b-5p|hsa-miR-27b-3p|hsa-miR-181d-5p
1393	CRHBP	repression	protein-coding					0	1.00E+00	
200186	CRTC2	repression	protein-coding	Yes				0	1.00E+00	
1434	CSE1L	activation	protein-coding					0	1.00E+00	
1465	CSRP1	repression	protein-coding					0	1.00E+00	
1520	CTSS	repression	protein-coding					2	2.89E-01	hsa-miR-24-3p|hsa-miR-93-5p
348180	CTU2	activation	protein-coding					0	1.00E+00	
51706	CYB5R1	repression	protein-coding					1	4.29E-01	hsa-miR-25-3p
79901	CYBRD1	repression	protein-coding					2	7.92E-01	hsa-miR-124-3p|hsa-miR-93-5p
1594	CYP27B1	activation	protein-coding					0	1.00E+00	
285440	CYP4V2	repression	protein-coding					1	1.72E-01	hsa-miR-124-3p
1601	DAB2	repression	protein-coding					3	2.66E-01	hsa-miR-93-5p|hsa-miR-320a|hsa-miR-124-3p
26528	DAZAP1	activation	protein-coding					2	1.78E-01	hsa-miR-24-3p|hsa-miR-320a
84067	FAM160A2	repression	protein-coding					4	1.45E-03	hsa-miR-25-3p|hsa-miR-181d-5p|hsa-miR-181a-5p|hsa-miR-181b-5p
10926	DBF4	activation	protein-coding					2	1.29E-01	hsa-miR-330-3p|hsa-miR-320a
220042	DDIAS	activation	protein-coding					2	1.99E-01	hsa-miR-93-5p|hsa-miR-124-3p
1653	DDX1	activation	protein-coding					0	1.00E+00	
79039	DDX54	activation	protein-coding					1	5.59E-01	hsa-miR-320a
55601	DDX60	repression	protein-coding					0	1.00E+00	
50619	DEF6	repression	protein-coding					0	1.00E+00	
79961	DENND2D	repression	protein-coding					0	1.00E+00	
55789	DEPDC1B	activation	protein-coding					2	8.43E-02	hsa-miR-27a-3p|hsa-miR-27b-3p
1719	DHFR	activation	protein-coding					0	1.00E+00	
56919	DHX33	activation	protein-coding					1	8.11E-01	hsa-miR-24-3p
79132	DHX58	repression	protein-coding					0	1.00E+00	
26118	WSB1	repression	protein-coding					7	1.47E-03	hsa-miR-320a|hsa-miR-181d-5p|hsa-miR-181a-5p|hsa-miR-181b-5p|hsa-miR-124-3p|hsa-miR-27a-3p|hsa-miR-27b-3p
9077	DIRAS3	repression	protein-coding					0	1.00E+00	
1736	DKC1	activation	protein-coding					0	1.00E+00	
10683	DLL3	activation	protein-coding					0	1.00E+00	
1750	DLX6	activation	protein-coding	Yes				1	8.04E-01	hsa-miR-330-3p
55172	DNAAF2	activation	protein-coding					0	1.00E+00	

7049	TGFBR3	repression	protein-coding	Yes			7	1.65E-03	hsa-miR-330-3p|hsa-miR-181a-5p|hsa-miR-181d-5p|hsa-miR-181b-5p|hsa-miR-27b-3p|hsa-miR-27a-3p|hsa-miR-296-5p
9829	DNAJC6	repression	protein-coding			Yes	4	1.38E-01	hsa-miR-181d-5p|hsa-miR-27b-3p|hsa-miR-27a-3p|hsa-miR-181b-5p
8448	DOC2A	activation	protein-coding				0	1.00E+00	
406	ARNTL	repression	protein-coding	Yes			5	1.70E-03	hsa-miR-7-5p|hsa-miR-760|hsa-miR-27a-3p|hsa-miR-320a|hsa-miR-27b-3p
55332	DRAM1	repression	protein-coding				1	1.00E+00	hsa-miR-124-3p
92749	DRC1	repression	protein-coding				0	1.00E+00	
79075	DSCC1	activation	protein-coding				1	6.28E-01	hsa-miR-320a
113878	DTX2	repression	protein-coding				1	6.13E-01	hsa-miR-25-3p
1841	DTYMK	activation	protein-coding				1	7.56E-02	hsa-miR-7-5p
56931	DUS3L	activation	protein-coding				0	1.00E+00	
3675	ITGA3	repression	protein-coding				6	2.44E-03	hsa-miR-24-3p|hsa-miR-296-5p|hsa-miR-181d-5p|hsa-miR-181a-5p|hsa-miR-181b-5p|hsa-miR-124-3p
54808	DYM	activation	protein-coding				0	1.00E+00	
1869	E2F1	activation	protein-coding	Yes			3	1.25E-01	hsa-miR-330-3p|hsa-miR-296-5p|hsa-miR-93-5p
51512	GTSE1	activation	protein-coding				3	2.54E-03	hsa-miR-181d-5p|hsa-miR-181a-5p|hsa-miR-181b-5p
1889	ECE1	repression	protein-coding				3	5.64E-02	hsa-miR-27a-3p|hsa-miR-124-3p|hsa-miR-27b-3p
55268	ECHDC2	repression	protein-coding				0	1.00E+00	
1894	ECT2	activation	protein-coding				3	1.33E-01	hsa-miR-33a-5p|hsa-miR-27a-3p|hsa-miR-27b-3p
55629	PNRC2	repression	protein-coding				6	2.54E-03	hsa-miR-2110|hsa-miR-181d-5p|hsa-miR-181a-5p|hsa-miR-181b-5p|hsa-miR-27a-3p|hsa-miR-27b-3p
1933	EEF1B2	activation	protein-coding				0	1.00E+00	
1937	EEF1G	activation	protein-coding				0	1.00E+00	
9813	EFCAB14	repression	protein-coding				5	7.49E-02	hsa-miR-124-3p|hsa-miR-27a-3p|hsa-miR-24-3p|hsa-miR-93-5p|hsa-miR-27b-3p
1978	EIF4EBP1	activation	protein-coding				0	1.00E+00	
1995	ELAVL3	activation	protein-coding				1	1.00E+00	hsa-miR-296-5p
80237	ELL3	repression	protein-coding				0	1.00E+00	
63916	ELMO2	repression	protein-coding				2	6.28E-01	hsa-miR-24-3p|hsa-miR-124-3p

79071	ELOVL6	activation	protein-coding					3	4.01E-01	hsa-miR-320a|hsa-miR-330-3p|hsa-miR-25-3p
23065	EMC1	repression	protein-coding					1	7.80E-01	hsa-miR-24-3p
51016	EMC9	activation	protein-coding					0	1.00E+00	
256364	EML3	repression	protein-coding					0	1.00E+00	
2013	EMP2	repression	protein-coding					1	6.86E-01	hsa-miR-24-3p
23052	ENDOD1	repression	protein-coding					1	8.11E-01	hsa-miR-124-3p
2021	ENDOG	activation	protein-coding	Yes				0	1.00E+00	
83786	FRMD8	repression	protein-coding					4	2.77E-03	hsa-miR-124-3p|hsa-miR-181d-5p|hsa-miR-181a-5p|hsa-miR-181b-5p
2036	EPB41L1	repression	protein-coding					4	3.30E-01	hsa-miR-33a-5p|hsa-miR-27b-3p|hsa-miR-330-3p|hsa-miR-27a-3p
58513	EPS15L1	activation	protein-coding					0	1.00E+00	
10492	SYNCRIP	activation	protein-coding					9	3.04E-03	hsa-miR-33a-5p|hsa-miR-181d-5p|hsa-miR-124-3p|hsa-miR-93-5p|hsa-miR-320a|hsa-miR-27b-3p|hsa-miR-27a-3p|hsa-miR-181a-5p|hsa-miR-181b-5p
51752	ERAP1	repression	protein-coding					3	2.16E-01	hsa-miR-330-3p|hsa-miR-33a-5p|hsa-miR-93-5p
55914	ERBB2IP	repression	protein-coding					3	7.69E-01	hsa-miR-33a-5p|hsa-miR-93-5p|hsa-miR-25-3p
11228	RASSF8	repression	protein-coding					7	4.04E-03	hsa-miR-33a-5p|hsa-miR-181d-5p|hsa-miR-124-3p|hsa-miR-320a|hsa-miR-27b-3p|hsa-miR-181a-5p|hsa-miR-181b-5p
5547	PRCP	repression	protein-coding					4	4.51E-03	hsa-miR-7-5p|hsa-miR-181d-5p|hsa-miR-181a-5p|hsa-miR-181b-5p
157570	ESCO2	activation	protein-coding					1	6.86E-01	hsa-miR-24-3p
2120	ETV6	repression	protein-coding	Yes				0	1.00E+00	
51513	ETV7	repression	protein-coding	Yes				0	1.00E+00	
56915	EXOSC5	activation	protein-coding					0	1.00E+00	
3995	FADS3	repression	protein-coding					1	7.56E-02	hsa-miR-7-5p
114625	ERMAP	repression	protein-coding					3	4.72E-03	hsa-miR-181d-5p|hsa-miR-181a-5p|hsa-miR-181b-5p
63901	FAM111A	repression	protein-coding					0	1.00E+00	
5863	RGL2	repression	protein-coding					3	4.72E-03	hsa-miR-27a-3p|hsa-miR-24-3p|hsa-miR-27b-3p
84908	FAM136A	activation	protein-coding					0	1.00E+00	
135892	TRIM50	repression	protein-coding					2	4.82E-03	hsa-miR-27a-3p|hsa-miR-27b-3p
85395	FAM207A	activation	protein-coding					0	1.00E+00	
127281	FAM213B	repression	protein-coding					0	1.00E+00	

29902	FAM216A	activation	protein-coding				0	1.00E+00	
10447	FAM3C	repression	protein-coding				4	6.06E-02	hsa-miR-181a-5p|hsa-miR-93-5p|hsa-miR-181d-5p|hsa-miR-181b-5p
58516	FAM60A	activation	protein-coding				1	1.00E+00	hsa-miR-93-5p
54478	FAM64A	activation	protein-coding				1	3.18E-01	hsa-miR-330-3p
9750	FAM65B	repression	protein-coding				2	2.58E-01	hsa-miR-27a-3p|hsa-miR-27b-3p
138311	FAM69B	activation	protein-coding				1	2.10E-01	hsa-miR-320a
728833	FAM72D	activation	protein-coding				0	1.00E+00	
374986	FAM73A	repression	protein-coding				0	1.00E+00	
50618	ITSN2	repression	protein-coding				5	5.22E-03	hsa-miR-93-5p|hsa-miR-320a|hsa-miR-124-3p|hsa-miR-27b-3p|hsa-miR-27a-3p
81610	FAM83D	activation	protein-coding				0	1.00E+00	
2177	FANCD2	activation	protein-coding				0	1.00E+00	
92565	FANK1	repression	protein-coding				0	1.00E+00	
84108	PCGF6	activation	protein-coding	Yes			5	5.64E-03	hsa-miR-25-3p|hsa-miR-7-5p|hsa-miR-93-5p|hsa-miR-181a-5p|hsa-miR-181b-5p
2194	FASN	activation	protein-coding				0	1.00E+00	
5583	PRKCH	repression	protein-coding		Yes		4	5.98E-03	hsa-miR-24-3p|hsa-miR-93-5p|hsa-miR-27a-3p|hsa-miR-27b-3p
2091	FBL	activation	protein-coding				0	1.00E+00	
26273	FBXO3	repression	protein-coding				3	1.45E-01	hsa-miR-330-3p|hsa-miR-7-5p|hsa-miR-124-3p
54455	FBXO42	repression	protein-coding				0	1.00E+00	
51060	TXNDC12	repression	protein-coding				4	5.98E-03	hsa-miR-24-3p|hsa-miR-181d-5p|hsa-miR-181a-5p|hsa-miR-181b-5p
26268	FBXO9	repression	protein-coding				2	4.68E-01	hsa-miR-33a-5p|hsa-miR-93-5p
26127	FGFR1OP2	repression	protein-coding				1	4.89E-01	hsa-miR-24-3p
24147	FJX1	activation	protein-coding				2	5.26E-01	hsa-miR-93-5p|hsa-miR-320a
2286	FKBP2	repression	protein-coding				0	1.00E+00	
2288	FKBP4	activation	protein-coding				0	1.00E+00	
51661	FKBP7	activation	protein-coding				0	1.00E+00	
11328	FKBP9	repression	protein-coding				0	1.00E+00	
80308	FLAD1	activation	protein-coding				1	1.27E-01	hsa-miR-7-5p
55640	FLVCR2	repression	protein-coding				0	1.00E+00	
55810	FOXJ2	repression	protein-coding	Yes			3	4.94E-01	hsa-miR-7-5p|hsa-miR-93-5p|hsa-miR-330-3p
2305	FOXM1	activation	protein-coding	Yes			1	7.05E-01	hsa-miR-320a
2308	FOXO1	repression	protein-coding	Yes			4	3.17E-01	hsa-miR-320a|hsa-miR-27b-3p|hsa-miR-330-3p|hsa-miR-27a-3p
8790	FPGT	repression	protein-coding				0	1.00E+00	

6249	CLIP1	repression	protein-coding				6	7.15E-03	hsa-miR-330-3p|hsa-miR-7-5p|hsa-miR-181d-5p|hsa-miR-181a-5p|hsa-miR-181b-5p|hsa-miR-124-3p
10272	FSTL3	repression	protein-coding				1	5.59E-01	hsa-miR-296-5p
24140	FTSJ1	activation	protein-coding				0	1.00E+00	
282969	FUOM	repression	protein-coding				0	1.00E+00	
2521	FUS	activation	protein-coding				1	3.76E-01	hsa-miR-320a
23710	GABARAPL1	repression	protein-coding				0	1.00E+00	
1647	GADD45A	repression	protein-coding				0	1.00E+00	
10634	GAS2L1	repression	protein-coding				2	1.03E-01	hsa-miR-124-3p|hsa-miR-760
60674	GAS5	activation	ncRNA				0	1.00E+00	
54815	GATAD2A	activation	protein-coding	Yes			3	6.61E-02	hsa-miR-124-3p|hsa-miR-27a-3p|hsa-miR-27b-3p
2628	GATM	repression	protein-coding				3	2.32E-01	hsa-miR-181a-5p|hsa-miR-181d-5p|hsa-miR-181b-5p
2642	GCGR	repression	protein-coding				0	1.00E+00	
2672	GFI1	repression	protein-coding	Yes			0	1.00E+00	
51659	GINS2	activation	protein-coding				0	1.00E+00	
10052	GJC1	activation	protein-coding				5	5.72E-02	hsa-miR-124-3p|hsa-miR-24-3p|hsa-miR-27b-3p|hsa-miR-27a-3p|hsa-miR-7-5p
8833	GMPS	activation	protein-coding				0	1.00E+00	
2768	GNA12	repression	protein-coding				1	7.80E-01	hsa-miR-7-5p
2782	GNB1	repression	protein-coding				0	1.00E+00	
10399	GNB2L1	activation	protein-coding				0	1.00E+00	
55970	GNG12	repression	protein-coding				2	1.00E+00	hsa-miR-27b-3p|hsa-miR-27a-3p
26354	GNL3	activation	protein-coding				0	1.00E+00	
84572	GNPTG	repression	protein-coding				0	1.00E+00	
2806	GOT2	activation	protein-coding				3	8.58E-02	hsa-miR-181b-5p|hsa-miR-181d-5p|hsa-miR-181a-5p
10243	GPHN	activation	protein-coding				1	8.11E-01	hsa-miR-330-3p
81491	GPR63	activation	protein-coding				1	8.11E-01	hsa-miR-93-5p
2874	GPS2	repression	protein-coding				0	1.00E+00	
160622	GRASP	repression	protein-coding				0	1.00E+00	
83903	GSG2	activation	protein-coding		Yes		0	1.00E+00	
9567	GTPBP1	activation	protein-coding				1	8.78E-01	hsa-miR-24-3p
51422	PRKAG2	repression	protein-coding				6	7.15E-03	hsa-miR-7-5p|hsa-miR-181a-5p|hsa-miR-181b-5p|hsa-miR-320a|hsa-miR-124-3p|hsa-miR-181d-5p
8971	H1FX	activation	protein-coding				0	1.00E+00	
55506	H2AFY2	activation	protein-coding				0	1.00E+00	
93323	HAUS8	activation	protein-coding				1	6.13E-01	hsa-miR-93-5p
3049	HBQ1	activation	protein-coding				0	1.00E+00	

3068	HDGF	activation	protein-coding				1	1.00E+00	hsa-miR-345-5p
65009	NDRG4	repression	protein-coding				5	8.06E-03	hsa-miR-24-3p|hsa-miR-760|hsa-miR-181d-5p|hsa-miR-181a-5p|hsa-miR-181b-5p
3280	HES1	repression	protein-coding	Yes			0	1.00E+00	
3087	HHEX	repression	protein-coding	Yes			0	1.00E+00	
4853	NOTCH2	repression	protein-coding	Yes			8	8.12E-03	hsa-miR-181d-5p|hsa-miR-124-3p|hsa-miR-7-5p|hsa-miR-93-5p|hsa-miR-27b-3p|hsa-miR-27a-3p|hsa-miR-181a-5p|hsa-miR-181b-5p
80201	HKDC1	repression	protein-coding				0	1.00E+00	
3106	HLA-B	repression	protein-coding				0	1.00E+00	
3113	HLA-DPA1	repression	protein-coding				0	1.00E+00	
3167	HMX2	activation	protein-coding	Yes			0	1.00E+00	
10949	HNRNPA0	activation	protein-coding				0	1.00E+00	
220988	HNRNPA3	activation	protein-coding				1	1.00E+00	hsa-miR-33a-5p
9987	HNRNPDL	activation	protein-coding				1	1.00E+00	hsa-miR-330-3p
3201	HOXA4	repression	protein-coding	Yes			0	1.00E+00	
3232	HOXD3	activation	protein-coding	Yes			0	1.00E+00	
84842	HPDL	activation	protein-coding				0	1.00E+00	
11255	HRH3	activation	protein-coding				0	1.00E+00	
3313	HSPA9	activation	protein-coding				0	1.00E+00	
26353	HSPB8	repression	protein-coding				1	4.00E-01	hsa-miR-124-3p
3336	HSPE1	activation	protein-coding				0	1.00E+00	
81888	HYI	repression	protein-coding				0	1.00E+00	
23125	CAMTA2	repression	protein-coding	Yes			4	8.19E-03	hsa-miR-93-5p|hsa-miR-181d-5p|hsa-miR-181a-5p|hsa-miR-181b-5p
3383	ICAM1	repression	protein-coding	Yes			0	1.00E+00	
25900	IFFO1	repression	protein-coding				0	1.00E+00	
80212	CCDC92	repression	protein-coding				3	8.22E-03	hsa-miR-181d-5p|hsa-miR-181a-5p|hsa-miR-181b-5p
57549	IGSF9	activation	protein-coding				0	1.00E+00	
3597	IL13RA1	repression	protein-coding				4	5.48E-02	hsa-miR-330-3p|hsa-miR-7-5p|hsa-miR-27a-3p|hsa-miR-27b-3p
55707	NECAP2	repression	protein-coding				3	8.22E-03	hsa-miR-124-3p|hsa-miR-27b-3p|hsa-miR-27a-3p
50615	IL21R	repression	protein-coding				1	2.10E-01	hsa-miR-24-3p
9235	IL32	repression	protein-coding				0	1.00E+00	
3570	IL6R	repression	protein-coding				2	4.68E-01	hsa-miR-7-5p|hsa-miR-124-3p
5464	PPA1	activation	protein-coding			Yes	3	8.22E-03	hsa-miR-27a-3p|hsa-miR-320a|hsa-miR-27b-3p
3611	ILK	repression	protein-coding		Yes		0	1.00E+00	

11313	LYPLA2	repression	protein-coding				5	8.33E-03	hsa-miR-24-3p|hsa-miR-296-5p|hsa-miR-181a-5p|hsa-miR-181b-5p|hsa-miR-27b-3p
9657	IQCB1	activation	protein-coding				0	1.00E+00	
8826	IQGAP1	repression	protein-coding				4	5.65E-02	hsa-miR-24-3p|hsa-miR-7-5p|hsa-miR-2110|hsa-miR-124-3p
10955	SERINC3	repression	protein-coding				5	8.91E-03	hsa-miR-330-3p|hsa-miR-33a-5p|hsa-miR-27a-3p|hsa-miR-320a|hsa-miR-27b-3p
10745	PHTF1	repression	protein-coding	Yes			2	9.45E-03	hsa-miR-27a-3p|hsa-miR-27b-3p
3679	ITGA7	repression	protein-coding				1	2.48E-01	hsa-miR-124-3p
3687	ITGAX	repression	protein-coding				0	1.00E+00	
3689	ITGB2	repression	protein-coding	Yes			0	1.00E+00	
150771	ITPRIPL1	activation	protein-coding				0	1.00E+00	
10826	FAXDC2	repression	protein-coding				3	9.66E-03	hsa-miR-124-3p|hsa-miR-27a-3p|hsa-miR-27b-3p
9767	JADE3	activation	protein-coding				1	1.00E+00	hsa-miR-7-5p
84522	JAGN1	activation	protein-coding				0	1.00E+00	
3716	JAK1	repression	protein-coding		Yes		1	1.00E+00	hsa-miR-93-5p
8629	JRK	activation	protein-coding				0	1.00E+00	
3726	JUNB	repression	protein-coding	Yes			0	1.00E+00	
10300	KATNB1	activation	protein-coding				1	4.55E-01	hsa-miR-24-3p
8514	KCNAB2	repression	protein-coding				0	1.00E+00	
115207	KCTD12	repression	protein-coding				5	1.55E-01	hsa-miR-33a-5p|hsa-miR-24-3p|hsa-miR-296-5p|hsa-miR-27b-3p|hsa-miR-27a-3p
23081	KDM4C	repression	protein-coding				2	2.48E-01	hsa-miR-27a-3p|hsa-miR-27b-3p
9817	KEAP1	activation	protein-coding	Yes			0	1.00E+00	
79932	KIAA0319L	repression	protein-coding				2	2.48E-01	hsa-miR-27a-3p|hsa-miR-27b-3p
57456	KIAA1143	activation	protein-coding				1	4.68E-01	hsa-miR-7-5p
56243	KIAA1217	repression	protein-coding				2	6.45E-01	hsa-miR-7-5p|hsa-miR-320a
56992	KIF15	activation	protein-coding				0	1.00E+00	
130271	PLEKHH2	repression	protein-coding				5	9.92E-03	hsa-miR-25-3p|hsa-miR-24-3p|hsa-miR-7-5p|hsa-miR-27a-3p|hsa-miR-27b-3p
64782	AEN	activation	protein-coding				4	1.08E-02	hsa-miR-93-5p|hsa-miR-27a-3p|hsa-miR-320a|hsa-miR-27b-3p
3833	KIFC1	activation	protein-coding				0	1.00E+00	
90990	KIFC2	repression	protein-coding				0	1.00E+00	
9365	KL	repression	protein-coding				4	8.30E-02	hsa-miR-33a-5p|hsa-miR-181d-5p|hsa-miR-181a-5p|hsa-miR-181b-5p

688	KLF5	repression	protein-coding	Yes			3	3.35E-01	hsa-miR-25-3p|hsa-miR-320a|hsa-miR-124-3p
4189	DNAJB9	repression	protein-coding				5	1.08E-02	hsa-miR-25-3p|hsa-miR-27b-3p|hsa-miR-93-5p|hsa-miR-124-3p|hsa-miR-27a-3p
79786	KLHL36	repression	protein-coding				1	3.76E-01	hsa-miR-320a
57563	KLHL8	activation	protein-coding				1	8.89E-01	hsa-miR-124-3p
23633	KPNA6	repression	protein-coding				5	2.39E-01	hsa-miR-124-3p|hsa-miR-24-3p|hsa-miR-27b-3p|hsa-miR-27a-3p|hsa-miR-7-5p
144811	LACC1	repression	protein-coding				0	1.00E+00	
114294	LACTB	repression	protein-coding				1	6.28E-01	hsa-miR-33a-5p
3909	LAMA3	repression	protein-coding				1	5.98E-01	hsa-miR-93-5p
3916	LAMP1	repression	protein-coding				2	2.39E-01	hsa-miR-93-5p|hsa-miR-320a
3927	LASP1	repression	protein-coding				5	5.72E-02	hsa-miR-24-3p|hsa-miR-93-5p|hsa-miR-27b-3p|hsa-miR-27a-3p|hsa-miR-7-5p
26524	LATS2	repression	protein-coding		Yes		4	1.69E-01	hsa-miR-93-5p|hsa-miR-27b-3p|hsa-miR-25-3p|hsa-miR-27a-3p
3932	LCK	repression	protein-coding		Yes		0	1.00E+00	
3937	LCP2	repression	protein-coding				0	1.00E+00	
8861	LDB1	activation	protein-coding	Yes			0	1.00E+00	
26119	LDLRAP1	repression	protein-coding				3	6.61E-02	hsa-miR-93-5p|hsa-miR-124-3p|hsa-miR-320a
51176	LEF1	repression	protein-coding	Yes			2	6.54E-01	hsa-miR-93-5p|hsa-miR-320a
3953	LEPR	repression	protein-coding				2	3.02E-01	hsa-miR-24-3p|hsa-miR-320a
10186	LHFP	repression	protein-coding				2	2.89E-01	hsa-miR-27a-3p|hsa-miR-27b-3p
3980	LIG3	activation	protein-coding				0	1.00E+00	
1326	MAP3K8	repression	protein-coding		Yes		5	1.08E-02	hsa-miR-93-5p|hsa-miR-181a-5p|hsa-miR-181b-5p|hsa-miR-124-3p|hsa-miR-181d-5p
55788	LMBRD1	repression	protein-coding				1	4.68E-01	hsa-miR-7-5p
84823	LMNB2	activation	protein-coding				2	6.45E-02	hsa-miR-24-3p|hsa-miR-760
57121	LPAR5	repression	protein-coding				0	1.00E+00	
57326	PBXIP1	repression	protein-coding	Yes			3	1.08E-02	hsa-miR-27b-3p|hsa-miR-27a-3p|hsa-miR-24-3p
10162	LPCAT3	repression	protein-coding				1	7.24E-01	hsa-miR-124-3p
26018	LRIG1	repression	protein-coding				2	6.13E-01	hsa-miR-93-5p|hsa-miR-124-3p
83450	LRRC48	repression	protein-coding				0	1.00E+00	
149499	LRRC71	repression	protein-coding				0	1.00E+00	

50999	TMED5	repression	protein-coding				7	1.08E-02	hsa-miR-33a-5p|hsa-miR-27a-3p|hsa-miR-93-5p|hsa-miR-27b-3p|hsa-miR-25-3p|hsa-miR-181a-5p|hsa-miR-181b-5p
57633	LRRN1	activation	protein-coding				1	5.98E-01	hsa-miR-181b-5p
84967	LSM10	repression	protein-coding				1	7.56E-02	hsa-miR-24-3p
57819	LSM2	activation	protein-coding				0	1.00E+00	
27258	LSM3	activation	protein-coding				0	1.00E+00	
25804	LSM4	activation	protein-coding				0	1.00E+00	
51690	LSM7	activation	protein-coding				0	1.00E+00	
7940	LST1	repression	protein-coding				0	1.00E+00	
4050	LTB	repression	protein-coding				0	1.00E+00	
10006	ABI1	repression	protein-coding				6	1.08E-02	hsa-miR-33a-5p|hsa-miR-181d-5p|hsa-miR-93-5p|hsa-miR-25-3p|hsa-miR-181a-5p|hsa-miR-181b-5p
11127	KIF3A	repression	protein-coding				6	1.08E-02	hsa-miR-181d-5p|hsa-miR-124-3p|hsa-miR-27b-3p|hsa-miR-27a-3p|hsa-miR-181a-5p|hsa-miR-181b-5p
4869	NPM1	activation	protein-coding	Yes			5	1.08E-02	hsa-miR-33a-5p|hsa-miR-93-5p|hsa-miR-181d-5p|hsa-miR-181a-5p|hsa-miR-181b-5p
28992	MACROD1	activation	protein-coding				0	1.00E+00	
4121	MAN1A1	repression	protein-coding				3	3.35E-01	hsa-miR-25-3p|hsa-miR-7-5p|hsa-miR-320a
57134	MAN1C1	repression	protein-coding				1	9.60E-01	hsa-miR-93-5p
23324	MAN2B2	repression	protein-coding				0	1.00E+00	
4130	MAP1A	repression	protein-coding				2	4.68E-01	hsa-miR-24-3p|hsa-miR-2110
84557	MAP1LC3A	repression	protein-coding				0	1.00E+00	
7786	MAP3K12	repression	protein-coding		Yes		1	8.25E-01	hsa-miR-93-5p
4217	MAP3K5	repression	protein-coding		Yes		1	1.00E+00	hsa-miR-93-5p
7357	UGCG	repression	protein-coding				4	1.08E-02	hsa-miR-93-5p|hsa-miR-27b-3p|hsa-miR-27a-3p|hsa-miR-320a
377	ARF3	repression	protein-coding				7	1.12E-02	hsa-miR-181d-5p|hsa-miR-7-5p|hsa-miR-27b-3p|hsa-miR-320a|hsa-miR-181a-5p|hsa-miR-181b-5p|hsa-miR-27a-3p
10982	MAPRE2	repression	protein-coding				2	9.05E-01	hsa-miR-7-5p|hsa-miR-330-3p
115123	MARCH3	repression	protein-coding				1	8.04E-01	hsa-miR-124-3p
4150	MAZ	activation	protein-coding	Yes			1	8.49E-01	hsa-miR-7-5p
4171	MCM2	activation	protein-coding				0	1.00E+00	
4176	MCM7	activation	protein-coding				0	1.00E+00	
84515	MCM8	activation	protein-coding	Yes			0	1.00E+00	
255231	MCOLN2	repression	protein-coding				1	4.68E-01	hsa-miR-25-3p

79772	MCTP1	repression	protein-coding				0	1.00E+00	
4193	MDM2	activation	protein-coding	Yes			1	5.98E-01	hsa-miR-25-3p
64769	MEAF6	repression	protein-coding				3	1.72E-01	hsa-miR-181a-5p|hsa-miR-181b-5p|hsa-miR-124-3p
79006	METRN	repression	protein-coding				0	1.00E+00	
891	CCNB1	activation	protein-coding				4	1.12E-02	hsa-miR-93-5p|hsa-miR-181d-5p|hsa-miR-181a-5p|hsa-miR-181b-5p
399664	MEX3D	activation	protein-coding				2	4.89E-01	hsa-miR-93-5p|hsa-miR-320a
9927	MFN2	repression	protein-coding				1	1.00E+00	hsa-miR-93-5p
64747	MFSD1	repression	protein-coding				0	1.00E+00	
79778	MICALL2	repression	protein-coding				0	1.00E+00	
57708	MIER1	repression	protein-coding				3	8.48E-01	hsa-miR-7-5p|hsa-miR-320a|hsa-miR-25-3p
4282	MIF	activation	protein-coding				0	1.00E+00	
10643	IGF2BP3	activation	protein-coding				7	1.12E-02	hsa-miR-2110|hsa-miR-320a|hsa-miR-27b-3p|hsa-miR-330-3p|hsa-miR-27a-3p|hsa-miR-181a-5p|hsa-miR-181b-5p
197259	MLKL	repression	protein-coding		Yes		0	1.00E+00	
4343	MOV10	repression	protein-coding				0	1.00E+00	
255027	MPV17L	activation	protein-coding				0	1.00E+00	
6182	MRPL12	activation	protein-coding				0	1.00E+00	
29088	MRPL15	activation	protein-coding				0	1.00E+00	
11222	MRPL3	activation	protein-coding				0	1.00E+00	
51073	MRPL4	activation	protein-coding				1	2.10E-01	hsa-miR-24-3p
65080	MRPL44	activation	protein-coding				0	1.00E+00	
51642	MRPL48	activation	protein-coding				1	3.46E-01	hsa-miR-7-5p
51116	MRPS2	activation	protein-coding				0	1.00E+00	
9112	MTA1	activation	protein-coding	Yes			0	1.00E+00	
23787	MTCH1	repression	protein-coding				0	1.00E+00	
51001	MTERF3	activation	protein-coding				0	1.00E+00	
4520	MTF1	repression	protein-coding	Yes			2	6.13E-01	hsa-miR-93-5p|hsa-miR-124-3p
25902	MTHFD1L	activation	protein-coding				1	3.18E-01	hsa-miR-181d-5p
10797	MTHFD2	activation	protein-coding				3	1.25E-01	hsa-miR-25-3p|hsa-miR-7-5p|hsa-miR-33a-5p
4524	MTHFR	repression	protein-coding				1	1.00E+00	hsa-miR-24-3p
84206	MEX3B	activation	protein-coding				5	1.12E-02	hsa-miR-181d-5p|hsa-miR-181a-5p|hsa-miR-181b-5p|hsa-miR-320a|hsa-miR-27b-3p
79594	MUL1	repression	protein-coding				1	3.76E-01	hsa-miR-181d-5p
93343	MVB12A	activation	protein-coding				0	1.00E+00	
9961	MVP	repression	protein-coding				0	1.00E+00	
4605	MYBL2	activation	protein-coding	Yes			0	1.00E+00	
84073	MYCBPAP	repression	protein-coding				0	1.00E+00	

57644	MYH7B	repression	protein-coding				0	1.00E+00	
4641	MYO1C	repression	protein-coding				1	1.00E+00	hsa-miR-330-3p
55930	MYO5C	repression	protein-coding				1	8.49E-01	hsa-miR-93-5p
100820829	MYZAP	repression	protein-coding	Yes			0	1.00E+00	
653784	MZT2A	activation	protein-coding				0	1.00E+00	
8883	NAE1	activation	protein-coding				0	1.00E+00	
222236	NAPEPLD	repression	protein-coding				2	8.01E-01	hsa-miR-124-3p|hsa-miR-93-5p
79731	NARS2	activation	protein-coding				0	1.00E+00	
284565	NBPF15	repression	protein-coding				1	4.68E-01	hsa-miR-124-3p
64151	NCAPG	activation	protein-coding				0	1.00E+00	
23154	NCDN	repression	protein-coding				0	1.00E+00	
4691	NCL	activation	protein-coding				2	1.12E-01	hsa-miR-760|hsa-miR-181a-5p
4774	NFIA	repression	protein-coding	Yes			7	1.12E-02	hsa-miR-33a-5p|hsa-miR-124-3p|hsa-miR-27a-3p|hsa-miR-7-5p|hsa-miR-320a|hsa-miR-27b-3p|hsa-miR-25-3p
8509	NDST2	repression	protein-coding				1	5.98E-01	hsa-miR-24-3p
4731	NDUFV3	activation	protein-coding				0	1.00E+00	
204851	HIPK1	repression	protein-coding		Yes		9	1.19E-02	hsa-miR-33a-5p|hsa-miR-320a|hsa-miR-124-3p|hsa-miR-24-3p|hsa-miR-296-5p|hsa-miR-93-5p|hsa-miR-330-3p|hsa-miR-25-3p|hsa-miR-7-5p
26271	FBXO5	activation	protein-coding				3	1.32E-02	hsa-miR-181d-5p|hsa-miR-181a-5p|hsa-miR-181b-5p
79858	NEK11	repression	protein-coding		Yes		0	1.00E+00	
81831	NETO2	activation	protein-coding				3	6.13E-01	hsa-miR-93-5p|hsa-miR-27b-3p|hsa-miR-27a-3p
4779	NFE2L1	repression	protein-coding	Yes			2	4.63E-01	hsa-miR-330-3p|hsa-miR-124-3p
8809	IL18R1	repression	protein-coding				2	1.32E-02	hsa-miR-27a-3p|hsa-miR-27b-3p
4794	NFKBIE	repression	protein-coding	Yes			1	2.48E-01	hsa-miR-24-3p
9054	NFS1	repression	protein-coding				0	1.00E+00	
1389	CREBL2	repression	protein-coding	Yes			7	1.38E-02	hsa-miR-181d-5p|hsa-miR-24-3p|hsa-miR-27b-3p|hsa-miR-330-3p|hsa-miR-181a-5p|hsa-miR-181b-5p|hsa-miR-27a-3p
4830	NME1	activation	protein-coding				0	1.00E+00	
4831	NME2	activation	protein-coding	Yes			0	1.00E+00	
28987	NOB1	activation	protein-coding				0	1.00E+00	
10392	NOD1	repression	protein-coding	Yes			2	1.29E-01	hsa-miR-27b-3p|hsa-miR-27a-3p
8602	NOP14	activation	protein-coding				0	1.00E+00	

81669	CCNL2	repression	protein-coding				4	1.39E-02	hsa-miR-330-3p|hsa-miR-27a-3p|hsa-miR-320a|hsa-miR-27b-3p
148252	DIRAS1	activation	protein-coding				4	1.39E-02	hsa-miR-7-5p|hsa-miR-296-5p|hsa-miR-27a-3p|hsa-miR-27b-3p
8131	NPRL3	activation	protein-coding				0	1.00E+00	
4886	NPY1R	repression	protein-coding				0	1.00E+00	
4835	NQO2	repression	protein-coding				0	1.00E+00	
6641	SNTB1	repression	protein-coding				4	1.39E-02	hsa-miR-25-3p|hsa-miR-24-3p|hsa-miR-93-5p|hsa-miR-320a
3084	NRG1	repression	protein-coding				1	1.00E+00	hsa-miR-124-3p
4913	NTHL1	activation	protein-coding	Yes			0	1.00E+00	
55190	NUDT11	activation	protein-coding			Yes	1	1.00E+00	hsa-miR-7-5p
119369	NUDT9P1	repression	pseudo				0	1.00E+00	
51686	OAZ3	repression	protein-coding				1	2.48E-01	hsa-miR-25-3p
132299	OCIAD2	activation	protein-coding				0	1.00E+00	
4953	ODC1	activation	protein-coding				0	1.00E+00	
93145	OLFM2	activation	protein-coding				0	1.00E+00	
4985	OPRD1	activation	protein-coding	Yes			0	1.00E+00	
93129	ORAI3	repression	protein-coding	Yes			0	1.00E+00	
23594	ORC6	activation	protein-coding	Yes			0	1.00E+00	
5734	PTGER4	repression	protein-coding				5	1.57E-02	hsa-miR-25-3p|hsa-miR-24-3p|hsa-miR-93-5p|hsa-miR-27b-3p|hsa-miR-27a-3p
127700	OSCP1	repression	protein-coding				0	1.00E+00	
55214	P3H2	repression	protein-coding				0	1.00E+00	
8974	P4HA2	repression	protein-coding				1	8.11E-01	hsa-miR-124-3p
10606	PAICS	activation	protein-coding				0	1.00E+00	
51025	PAM16	activation	protein-coding				0	1.00E+00	
344838	PAQR9	activation	protein-coding				3	8.58E-02	hsa-miR-124-3p|hsa-miR-27a-3p|hsa-miR-27b-3p
54625	PARP14	repression	protein-coding				1	7.80E-01	hsa-miR-124-3p
51696	HECA	repression	protein-coding				6	1.61E-02	hsa-miR-33a-5p|hsa-miR-181d-5p|hsa-miR-93-5p|hsa-miR-320a|hsa-miR-181a-5p|hsa-miR-181b-5p
10336	PCGF3	activation	protein-coding				6	7.17E-02	hsa-miR-93-5p|hsa-miR-320a|hsa-miR-27b-3p|hsa-miR-330-3p|hsa-miR-25-3p|hsa-miR-27a-3p
5471	PPAT	activation	protein-coding				4	1.62E-02	hsa-miR-25-3p|hsa-miR-7-5p|hsa-miR-27a-3p|hsa-miR-27b-3p
23608	MKRN1	repression	protein-coding				6	1.65E-02	hsa-miR-33a-5p|hsa-miR-181d-5p|hsa-miR-93-5p|hsa-miR-330-3p|hsa-miR-181a-5p|hsa-miR-181b-5p

5111	PCNA	activation	protein-coding	Yes			0	1.00E+00	
8682	PEA15	repression	protein-coding				2	7.89E-01	hsa-miR-124-3p|hsa-miR-24-3p
4804	NGFR	repression	protein-coding				4	1.69E-02	hsa-miR-760|hsa-miR-296-5p|hsa-miR-27a-3p|hsa-miR-27b-3p
5195	PEX14	repression	protein-coding	Yes			0	1.00E+00	
5217	PFN2	activation	protein-coding				3	5.00E-01	hsa-miR-33a-5p|hsa-miR-7-5p|hsa-miR-93-5p
116154	PHACTR3	activation	protein-coding			Yes	0	1.00E+00	
148479	PHF13	repression	protein-coding				2	3.81E-01	hsa-miR-27a-3p|hsa-miR-27b-3p
112885	PHF21B	activation	protein-coding				0	1.00E+00	
8863	PER3	repression	protein-coding				4	1.69E-02	hsa-miR-181d-5p|hsa-miR-181a-5p|hsa-miR-181b-5p|hsa-miR-124-3p
254295	PHYHD1	repression	protein-coding				0	1.00E+00	
51604	PIGT	repression	protein-coding				0	1.00E+00	
57095	PITHD1	repression	protein-coding				2	1.48E-01	hsa-miR-27a-3p|hsa-miR-27b-3p
9600	PITPNM1	repression	protein-coding				0	1.00E+00	
57510	XPO5	activation	protein-coding				4	1.69E-02	hsa-miR-24-3p|hsa-miR-124-3p|hsa-miR-27a-3p|hsa-miR-27b-3p
11142	PKIG	repression	protein-coding				0	1.00E+00	
9088	PKMYT1	activation	protein-coding		Yes		2	7.19E-02	hsa-miR-24-3p|hsa-miR-93-5p
11145	PLA2G16	repression	protein-coding				0	1.00E+00	
51316	PLAC8	repression	protein-coding				0	1.00E+00	
5331	PLCB3	repression	protein-coding				0	1.00E+00	
55344	PLCXD1	activation	protein-coding				0	1.00E+00	
201164	PLD6	activation	protein-coding				0	1.00E+00	
79666	PLEKHF2	repression	protein-coding				1	1.00E+00	hsa-miR-124-3p
57449	PLEKHG5	repression	protein-coding				0	1.00E+00	
2908	NR3C1	repression	protein-coding	Yes			9	1.82E-02	hsa-miR-181d-5p|hsa-miR-124-3p|hsa-miR-92a-1-5p|hsa-miR-93-5p|hsa-miR-27b-3p|hsa-miR-27a-3p|hsa-miR-320a|hsa-miR-181a-5p|hsa-miR-181b-5p
9842	PLEKHM1	repression	protein-coding				3	1.90E-01	hsa-miR-25-3p|hsa-miR-93-5p|hsa-miR-760
80301	PLEKHO2	repression	protein-coding				1	8.89E-01	hsa-miR-93-5p
5347	PLK1	activation	protein-coding		Yes		0	1.00E+00	
57088	PLSCR4	repression	protein-coding				3	1.90E-01	hsa-miR-330-3p|hsa-miR-93-5p|hsa-miR-124-3p
84898	PLXDC2	repression	protein-coding				0	1.00E+00	
10154	PLXNC1	repression	protein-coding				1	9.48E-01	hsa-miR-320a
5376	PMP22	repression	protein-coding				0	1.00E+00	

57104	PNPLA2	repression	protein-coding				1	3.76E-01	hsa-miR-124-3p
50640	PNPLA8	repression	protein-coding				0	1.00E+00	
113402	SFT2D1	repression	protein-coding				3	1.82E-02	hsa-miR-181d-5p|hsa-miR-181a-5p|hsa-miR-181b-5p
5424	POLD1	activation	protein-coding				0	1.00E+00	
5427	POLE2	activation	protein-coding				0	1.00E+00	
54107	POLE3	activation	protein-coding				3	1.21E-01	hsa-miR-330-3p|hsa-miR-7-5p|hsa-miR-320a
5437	POLR2H	activation	protein-coding	Yes			0	1.00E+00	
5438	POLR2I	activation	protein-coding	Yes			0	1.00E+00	
661	POLR3D	activation	protein-coding				1	8.49E-01	hsa-miR-24-3p
84265	POLR3GL	repression	protein-coding				0	1.00E+00	
51728	POLR3K	activation	protein-coding				0	1.00E+00	
10585	POMT1	repression	protein-coding				0	1.00E+00	
6566	SLC16A1	activation	protein-coding				5	1.82E-02	hsa-miR-33a-5p|hsa-miR-320a|hsa-miR-124-3p|hsa-miR-27a-3p|hsa-miR-27b-3p
10248	POP7	activation	protein-coding				0	1.00E+00	
2065	ERBB3	repression	protein-coding		Yes		5	1.91E-02	hsa-miR-27a-3p|hsa-miR-24-3p|hsa-miR-93-5p|hsa-miR-124-3p|hsa-miR-27b-3p
8611	PPAP2A	repression	protein-coding			Yes	1	4.68E-01	hsa-miR-93-5p
5467	PPARD	repression	protein-coding	Yes			1	8.49E-01	hsa-miR-24-3p
9493	KIF23	activation	protein-coding				4	1.91E-02	hsa-miR-330-3p|hsa-miR-7-5p|hsa-miR-93-5p|hsa-miR-320a
8495	PPFIBP2	repression	protein-coding				1	3.46E-01	hsa-miR-124-3p
5525	PPP2R5A	repression	protein-coding			Yes	0	1.00E+00	
5526	PPP2R5B	repression	protein-coding			Yes	1	4.00E-01	hsa-miR-296-5p
6856	SYPL1	repression	protein-coding				5	2.24E-02	hsa-miR-25-3p|hsa-miR-181a-5p|hsa-miR-181d-5p|hsa-miR-181b-5p|hsa-miR-124-3p
7799	PRDM2	repression	protein-coding	Yes			2	1.00E+00	hsa-miR-33a-5p|hsa-miR-93-5p
7001	PRDX2	activation	protein-coding				0	1.00E+00	
10549	PRDX4	activation	protein-coding				0	1.00E+00	
5550	PREP	activation	protein-coding				1	4.00E-01	hsa-miR-27a-3p
8987	STBD1	repression	protein-coding				3	2.68E-02	hsa-miR-93-5p|hsa-miR-27a-3p|hsa-miR-27b-3p
9867	PJA2	repression	protein-coding				6	2.69E-02	hsa-miR-33a-5p|hsa-miR-181d-5p|hsa-miR-27b-3p|hsa-miR-27a-3p|hsa-miR-181a-5p|hsa-miR-181b-5p
5590	PRKCZ	repression	protein-coding		Yes		0	1.00E+00	
5613	PRKX	activation	protein-coding		Yes		3	7.91E-02	hsa-miR-124-3p|hsa-miR-27b-3p|hsa-miR-27a-3p
3276	PRMT1	activation	protein-coding				0	1.00E+00	
3275	PRMT2	repression	protein-coding	Yes			1	7.56E-02	hsa-miR-7-5p

29968	PSAT1	activation	protein-coding				0	1.00E+00	
84249	PSD2	repression	protein-coding				0	1.00E+00	
286826	LIN9	activation	protein-coding				4	2.77E-02	hsa-miR-33a-5p|hsa-miR-7-5p|hsa-miR-27a-3p|hsa-miR-27b-3p
9051	PSTPIP1	repression	protein-coding				0	1.00E+00	
5730	PTGDS	repression	protein-coding				0	1.00E+00	
23678	SGK3	repression	protein-coding		Yes		5	2.86E-02	hsa-miR-25-3p|hsa-miR-7-5p|hsa-miR-181d-5p|hsa-miR-181a-5p|hsa-miR-181b-5p
5757	PTMA	activation	protein-coding				0	1.00E+00	
8073	PTP4A2	repression	protein-coding			Yes	2	4.68E-01	hsa-miR-7-5p|hsa-miR-93-5p
5782	PTPN12	repression	protein-coding			Yes	2	6.13E-01	hsa-miR-320a|hsa-miR-124-3p
5784	PTPN14	repression	protein-coding			Yes	0	1.00E+00	
26191	PTPN22	repression	protein-coding			Yes	0	1.00E+00	
5777	PTPN6	repression	protein-coding			Yes	0	1.00E+00	
5792	PTPRF	repression	protein-coding			Yes	1	1.00E+00	hsa-miR-24-3p
284119	PTRF	repression	protein-coding	Yes			1	8.25E-01	hsa-miR-24-3p
10744	PTTG2	activation	protein-coding				0	1.00E+00	
54517	PUS7	activation	protein-coding				3	7.08E-02	hsa-miR-25-3p|hsa-miR-124-3p|hsa-miR-320a
221749	PXDC1	repression	protein-coding				0	1.00E+00	
5829	PXN	repression	protein-coding				2	2.66E-01	hsa-miR-2110|hsa-miR-7-5p
5831	PYCR1	activation	protein-coding				0	1.00E+00	
5837	PYGM	repression	protein-coding				0	1.00E+00	
5862	RAB2A	repression	protein-coding				1	8.04E-01	hsa-miR-124-3p
326624	RAB37	repression	protein-coding				0	1.00E+00	
5865	RAB3B	repression	protein-coding				0	1.00E+00	
339122	RAB43	repression	protein-coding				1	8.25E-01	hsa-miR-760
51762	RAB8B	repression	protein-coding				7	7.56E-02	hsa-miR-181a-5p|hsa-miR-181d-5p|hsa-miR-27a-3p|hsa-miR-93-5p|hsa-miR-27b-3p|hsa-miR-25-3p|hsa-miR-181b-5p
8320	EOMES	repression	protein-coding	Yes			3	2.89E-02	hsa-miR-330-3p|hsa-miR-25-3p|hsa-miR-124-3p
55684	RABL6	activation	protein-coding				0	1.00E+00	
25788	RAD54B	activation	protein-coding				0	1.00E+00	
5899	RALB	repression	protein-coding				0	1.00E+00	
5902	RANBP1	activation	protein-coding				0	1.00E+00	
10411	RAPGEF3	repression	protein-coding				1	3.46E-01	hsa-miR-25-3p
83937	RASSF4	repression	protein-coding				0	1.00E+00	
83593	RASSF5	repression	protein-coding				4	5.30E-02	hsa-miR-27b-3p|hsa-miR-93-5p|hsa-miR-124-3p|hsa-miR-27a-3p
8045	RASSF7	repression	protein-coding				0	1.00E+00	

9885	OSBPL2	repression	protein-coding					4	2.89E-02	hsa-miR-93-5p|hsa-miR-181d-5p|hsa-miR-181a-5p|hsa-miR-181b-5p
125950	RAVER1	activation	protein-coding					1	7.92E-01	hsa-miR-124-3p
388403	YPEL2	repression	protein-coding					6	3.30E-02	hsa-miR-92a-1-5p|hsa-miR-27a-3p|hsa-miR-7-5p|hsa-miR-93-5p|hsa-miR-27b-3p|hsa-miR-330-3p
83758	RBP5	repression	protein-coding					0	1.00E+00	
91433	RCCD1	activation	protein-coding					1	5.81E-01	hsa-miR-93-5p
283248	RCOR2	activation	protein-coding	Yes				0	1.00E+00	
5959	RDH5	repression	protein-coding					0	1.00E+00	
9401	RECQL4	activation	protein-coding					0	1.00E+00	
28954	REM1	repression	protein-coding					0	1.00E+00	
11079	RER1	repression	protein-coding					1	5.81E-01	hsa-miR-24-3p
79785	RERGL	repression	protein-coding					0	1.00E+00	
55159	RFWD3	activation	protein-coding					2	3.94E-01	hsa-miR-330-3p|hsa-miR-124-3p
23179	RGL1	repression	protein-coding					3	2.66E-01	hsa-miR-25-3p|hsa-miR-7-5p|hsa-miR-93-5p
8992	ATP6V0E1	repression	protein-coding					3	3.43E-02	hsa-miR-33a-5p|hsa-miR-93-5p|hsa-miR-124-3p
5998	RGS3	repression	protein-coding					1	6.86E-01	hsa-miR-25-3p
6006	RHCE	repression	protein-coding					0	1.00E+00	
389	RHOC	repression	protein-coding					1	6.86E-01	hsa-miR-93-5p
399	RHOH	repression	protein-coding					0	1.00E+00	
83547	RILP	repression	protein-coding					0	1.00E+00	
6015	RING1	repression	protein-coding					1	3.46E-01	hsa-miR-1268a
8737	RIPK1	repression	protein-coding		Yes			1	2.10E-01	hsa-miR-24-3p
11035	RIPK3	repression	protein-coding		Yes			0	1.00E+00	
134701	RIPPLY2	activation	protein-coding					0	1.00E+00	
6038	RNASE4	repression	protein-coding					0	1.00E+00	
10535	RNASEH2A	activation	protein-coding					0	1.00E+00	
26994	RNF11	repression	protein-coding					5	6.26E-02	hsa-miR-124-3p|hsa-miR-24-3p|hsa-miR-93-5p|hsa-miR-25-3p|hsa-miR-7-5p
9604	RNF14	repression	protein-coding					1	8.11E-01	hsa-miR-7-5p
57484	RNF150	activation	protein-coding					1	6.13E-01	hsa-miR-320a
114804	RNF157	activation	protein-coding					0	1.00E+00	
127544	RNF19B	repression	protein-coding					0	1.00E+00	
57674	RNF213	repression	protein-coding					1	7.45E-01	hsa-miR-124-3p
11237	RNF24	activation	protein-coding					2	4.42E-01	hsa-miR-24-3p|hsa-miR-93-5p
200916	RPL22L1	activation	protein-coding					0	1.00E+00	
6147	RPL23A	activation	protein-coding					0	1.00E+00	
6159	RPL29	activation	protein-coding					0	1.00E+00	
25873	RPL36	activation	protein-coding					0	1.00E+00	
6125	RPL5	activation	protein-coding					0	1.00E+00	

6205	RPS11	activation	protein-coding				0	1.00E+00	
6210	RPS15A	activation	protein-coding				0	1.00E+00	
6187	RPS2	activation	protein-coding				0	1.00E+00	
6189	RPS3A	activation	protein-coding	Yes			0	1.00E+00	
113000	RPUSD1	activation	protein-coding				0	1.00E+00	
64121	RRAGC	repression	protein-coding				0	1.00E+00	
9136	RRP9	activation	protein-coding				0	1.00E+00	
26156	RSL1D1	activation	protein-coding				0	1.00E+00	
345895	RSPH4A	repression	protein-coding				0	1.00E+00	
23348	DOCK9	repression	protein-coding				4	3.43E-02	hsa-miR-25-3p|hsa-miR-27a-3p|hsa-miR-93-5p|hsa-miR-27b-3p
57035	RSRP1	repression	protein-coding				2	3.34E-01	hsa-miR-24-3p|hsa-miR-93-5p
25914	RTTN	activation	protein-coding				1	5.11E-01	hsa-miR-124-3p
146923	RUNDC1	repression	protein-coding				1	6.28E-01	hsa-miR-93-5p
864	RUNX3	repression	protein-coding	Yes			3	2.03E-01	hsa-miR-330-3p|hsa-miR-93-5p|hsa-miR-345-5p
6281	S100A10	repression	protein-coding				0	1.00E+00	
6284	S100A13	repression	protein-coding				0	1.00E+00	
6285	S100B	repression	protein-coding				1	1.27E-01	hsa-miR-330-3p
56940	DUSP22	repression	protein-coding			Yes	3	3.43E-02	hsa-miR-181d-5p|hsa-miR-181a-5p|hsa-miR-181b-5p
113174	SAAL1	activation	protein-coding				0	1.00E+00	
154075	SAMD3	repression	protein-coding				0	1.00E+00	
8819	SAP30	activation	protein-coding				0	1.00E+00	
89958	SAPCD2	activation	protein-coding				0	1.00E+00	
54938	SARS2	activation	protein-coding				0	1.00E+00	
6303	SAT1	repression	protein-coding				1	5.98E-01	hsa-miR-320a
388228	SBK1	activation	protein-coding		Yes		1	1.00E+00	hsa-miR-124-3p
6332	SCN7A	repression	protein-coding				0	1.00E+00	
57758	SCUBE2	repression	protein-coding				1	4.68E-01	hsa-miR-7-5p
6386	SDCBP	repression	protein-coding	Yes			1	1.00E+00	hsa-miR-93-5p
51150	SDF4	repression	protein-coding				0	1.00E+00	
57001	SDHAF3	activation	protein-coding				0	1.00E+00	
1731	SEPT1	repression	protein-coding				0	1.00E+00	
55752	SEPT11	activation	protein-coding				3	4.01E-01	hsa-miR-124-3p|hsa-miR-27b-3p|hsa-miR-27a-3p
5414	SEPT4	repression	protein-coding				0	1.00E+00	
1871	E2F3	activation	protein-coding	Yes			7	3.43E-02	hsa-miR-33a-5p|hsa-miR-320a|hsa-miR-124-3p|hsa-miR-25-3p|hsa-miR-93-5p|hsa-miR-330-3p|hsa-miR-345-5p
1992	SERPINB1	repression	protein-coding				0	1.00E+00	
5271	SERPINB8	repression	protein-coding				0	1.00E+00	
6419	SETMAR	activation	protein-coding				0	1.00E+00	
8175	SF3A2	activation	protein-coding				0	1.00E+00	

57659	ZBTB4	repression	protein-coding	Yes			6	3.43E-02	hsa-miR-2110|hsa-miR-181d-5p|hsa-miR-7-5p|hsa-miR-93-5p|hsa-miR-181a-5p|hsa-miR-181b-5p
84826	SFT2D3	activation	protein-coding				1	8.41E-01	hsa-miR-124-3p
8910	SGCE	repression	protein-coding				0	1.00E+00	
4734	NEDD4	repression	protein-coding				6	3.50E-02	hsa-miR-33a-5p|hsa-miR-124-3p|hsa-miR-27a-3p|hsa-miR-7-5p|hsa-miR-93-5p|hsa-miR-27b-3p
151648	SGOL1	activation	protein-coding				0	1.00E+00	
10019	SH2B3	repression	protein-coding				5	8.82E-02	hsa-miR-33a-5p|hsa-miR-124-3p|hsa-miR-24-3p|hsa-miR-93-5p|hsa-miR-320a
10280	SIGMAR1	activation	protein-coding				1	7.24E-01	hsa-miR-7-5p
6494	SIPA1	repression	protein-coding				0	1.00E+00	
284611	FAM102B	repression	protein-coding				3	3.76E-02	hsa-miR-181d-5p|hsa-miR-181a-5p|hsa-miR-181b-5p
51296	SLC15A3	repression	protein-coding				0	1.00E+00	
116984	ARAP2	repression	protein-coding				5	3.77E-02	hsa-miR-181d-5p|hsa-miR-7-5p|hsa-miR-93-5p|hsa-miR-181a-5p|hsa-miR-181b-5p
6573	SLC19A1	activation	protein-coding				0	1.00E+00	
9673	SLC25A44	repression	protein-coding				2	4.00E-01	hsa-miR-27a-3p|hsa-miR-27b-3p
284129	SLC26A11	repression	protein-coding				2	8.43E-02	hsa-miR-27a-3p|hsa-miR-27b-3p
56731	SLC2A4RG	repression	protein-coding	Yes			2	1.22E-01	hsa-miR-320a|hsa-miR-93-5p
11182	SLC2A6	repression	protein-coding				0	1.00E+00	
7781	SLC30A3	activation	protein-coding				1	7.66E-01	hsa-miR-296-5p
1318	SLC31A2	repression	protein-coding				2	3.44E-01	hsa-miR-24-3p|hsa-miR-124-3p
92745	SLC38A5	activation	protein-coding				0	1.00E+00	
11309	SLCO2B1	repression	protein-coding				1	5.81E-01	hsa-miR-296-5p
64744	SMAP2	repression	protein-coding				1	1.00E+00	hsa-miR-2110
23642	SNHG1	activation	ncRNA				0	1.00E+00	
100093630	SNHG8	activation	ncRNA				0	1.00E+00	
6632	SNRPD1	activation	protein-coding				1	6.86E-01	hsa-miR-124-3p
6637	SNRPG	activation	protein-coding				0	1.00E+00	
3628	INPP1	repression	protein-coding			Yes	2	3.77E-02	hsa-miR-27a-3p|hsa-miR-27b-3p
257364	SNX33	repression	protein-coding				1	3.18E-01	hsa-miR-7-5p
51429	SNX9	repression	protein-coding				3	2.66E-01	hsa-miR-7-5p|hsa-miR-93-5p|hsa-miR-33a-5p
9576	SPAG6	repression	protein-coding				0	1.00E+00	
54558	SPATA6	repression	protein-coding				1	4.68E-01	hsa-miR-320a
9806	SPOCK2	repression	protein-coding				1	1.00E+00	hsa-miR-25-3p
84727	SPSB2	repression	protein-coding				0	1.00E+00	

6711	SPTBN1	repression	protein-coding				4	3.77E-01	hsa-miR-124-3p|hsa-miR-93-5p|hsa-miR-27b-3p|hsa-miR-27a-3p
58472	SQRDL	repression	protein-coding				0	1.00E+00	
8878	SQSTM1	repression	protein-coding				3	5.30E-02	hsa-miR-7-5p|hsa-miR-93-5p|hsa-miR-124-3p
80725	SRCIN1	repression	protein-coding				1	1.00E+00	hsa-miR-93-5p
6429	SRSF4	repression	protein-coding				0	1.00E+00	
6744	SSFA2	repression	protein-coding				3	4.32E-01	hsa-miR-124-3p|hsa-miR-25-3p|hsa-miR-93-5p
29101	SSU72	repression	protein-coding			Yes	0	1.00E+00	
10610	ST6GALNAC2	repression	protein-coding				0	1.00E+00	
6776	STAT5A	repression	protein-coding	Yes			0	1.00E+00	
388341	LRRC75A	repression	protein-coding				2	3.77E-02	hsa-miR-2110|hsa-miR-296-5p
6793	STK10	repression	protein-coding		Yes		3	5.64E-02	hsa-miR-24-3p|hsa-miR-27a-3p|hsa-miR-27b-3p
9263	STK17A	repression	protein-coding		Yes		0	1.00E+00	
8675	STX16	repression	protein-coding				5	8.30E-02	hsa-miR-2110|hsa-miR-24-3p|hsa-miR-27b-3p|hsa-miR-27a-3p|hsa-miR-7-5p
6813	STXBP2	repression	protein-coding				0	1.00E+00	
6832	SUPV3L1	activation	protein-coding				0	1.00E+00	
79723	SUV39H2	activation	protein-coding				2	6.54E-01	hsa-miR-33a-5p|hsa-miR-93-5p
55638	SYBU	repression	protein-coding				3	1.84E-01	hsa-miR-93-5p|hsa-miR-27b-3p|hsa-miR-27a-3p
10388	SYCP2	repression	protein-coding				0	1.00E+00	
10940	POP1	activation	protein-coding				2	3.77E-02	hsa-miR-27a-3p|hsa-miR-27b-3p
8871	SYNJ2	repression	protein-coding			Yes	1	6.71E-01	hsa-miR-24-3p
23336	SYNM	repression	protein-coding				1	1.00E+00	hsa-miR-93-5p
7185	TRAF1	repression	protein-coding	Yes			2	3.77E-02	hsa-miR-27b-3p|hsa-miR-27a-3p
84870	RSPO3	repression	protein-coding				3	3.95E-02	hsa-miR-24-3p|hsa-miR-27a-3p|hsa-miR-27b-3p
6867	TACC1	repression	protein-coding	Yes			3	9.04E-01	hsa-miR-124-3p|hsa-miR-93-5p|hsa-miR-25-3p
6883	TAF12	repression	protein-coding	Yes			1	9.72E-01	hsa-miR-7-5p
79101	TAF1D	activation	protein-coding	Yes			0	1.00E+00	
128989	TANGO2	repression	protein-coding				1	1.72E-01	hsa-miR-24-3p
8887	TAX1BP1	repression	protein-coding	Yes			0	1.00E+00	
55357	TBC1D2	repression	protein-coding				1	7.92E-01	hsa-miR-93-5p
6932	TCF7	repression	protein-coding	Yes			2	7.00E-01	hsa-miR-2110|hsa-miR-24-3p
7015	TERT	activation	protein-coding				0	1.00E+00	
7023	TFAP4	activation	protein-coding	Yes			2	4.20E-01	hsa-miR-760|hsa-miR-124-3p

22797	TFEC	repression	protein-coding	Yes			3	4.09E-01	hsa-miR-33a-5p|hsa-miR-124-3p|hsa-miR-7-5p
7048	TGFBR2	repression	protein-coding		Yes		5	1.05E-01	hsa-miR-181d-5p|hsa-miR-93-5p|hsa-miR-320a|hsa-miR-181a-5p|hsa-miR-181b-5p
286336	FAM78A	repression	protein-coding				4	4.34E-02	hsa-miR-33a-5p|hsa-miR-24-3p|hsa-miR-27a-3p|hsa-miR-27b-3p
51337	THEM6	activation	protein-coding				0	1.00E+00	
84321	THOC3	activation	protein-coding				0	1.00E+00	
7067	THRA	repression	protein-coding	Yes			4	5.09E-02	hsa-miR-2110|hsa-miR-93-5p|hsa-miR-27b-3p|hsa-miR-27a-3p
80745	THUMPD2	activation	protein-coding				0	1.00E+00	
7074	TIAM1	activation	protein-coding				4	1.74E-01	hsa-miR-320a|hsa-miR-24-3p|hsa-miR-93-5p|hsa-miR-7-5p
200765	TIGD1	activation	protein-coding				0	1.00E+00	
92609	TIMM50	activation	protein-coding			Yes	0	1.00E+00	
7098	TLR3	repression	protein-coding	Yes			0	1.00E+00	
3196	TLX2	activation	protein-coding				0	1.00E+00	
83941	TM2D1	repression	protein-coding				1	4.00E-01	hsa-miR-24-3p
51643	TMBIM4	repression	protein-coding				0	1.00E+00	
55330	BLOC1S4	activation	protein-coding				2	4.53E-02	hsa-miR-7-5p|hsa-miR-24-3p
284114	TMEM102	repression	protein-coding				0	1.00E+00	
222865	TMEM130	repression	protein-coding				0	1.00E+00	
25829	TMEM184B	repression	protein-coding				3	1.01E-01	hsa-miR-25-3p|hsa-miR-760|hsa-miR-124-3p
114801	TMEM200A	repression	protein-coding				0	1.00E+00	
84065	TMEM222	repression	protein-coding				0	1.00E+00	
56063	TMEM234	repression	protein-coding				0	1.00E+00	
55069	TMEM248	repression	protein-coding				4	9.06E-02	hsa-miR-33a-5p|hsa-miR-124-3p|hsa-miR-27b-3p|hsa-miR-27a-3p
23585	TMEM50A	repression	protein-coding				1	4.68E-01	hsa-miR-25-3p
283232	TMEM80	repression	protein-coding				0	1.00E+00	
7203	CCT3	activation	protein-coding				2	4.53E-02	hsa-miR-24-3p|hsa-miR-33a-5p
27346	TMEM97	activation	protein-coding				1	8.41E-01	hsa-miR-7-5p
355	FAS	repression	protein-coding				3	4.53E-02	hsa-miR-25-3p|hsa-miR-27a-3p|hsa-miR-27b-3p
286527	TMSB15B	activation	protein-coding				1	4.89E-01	hsa-miR-24-3p
25816	TNFAIP8	repression	protein-coding				2	4.98E-01	hsa-miR-93-5p|hsa-miR-124-3p
79626	TNFAIP8L2	repression	protein-coding				0	1.00E+00	
51330	TNFRSF12A	repression	protein-coding				0	1.00E+00	
8764	TNFRSF14	repression	protein-coding				0	1.00E+00	
23371	TNS2	repression	protein-coding			Yes	0	1.00E+00	

10043	TOM1	repression	protein-coding				1	5.81E-01	hsa-miR-124-3p
146691	TOM1L2	repression	protein-coding				0	1.00E+00	
9804	TOMM20	activation	protein-coding				3	3.18E-01	hsa-miR-181d-5p|hsa-miR-181a-5p|hsa-miR-181b-5p
58476	TP53INP2	repression	protein-coding				4	1.78E-01	hsa-miR-2110|hsa-miR-124-3p|hsa-miR-93-5p|hsa-miR-7-5p
27010	TPK1	repression	protein-coding				0	1.00E+00	
7172	TPMT	repression	protein-coding				1	5.11E-01	hsa-miR-25-3p
51673	TPPP3	repression	protein-coding				0	1.00E+00	
127262	TPRG1L	repression	protein-coding				1	5.98E-01	hsa-miR-93-5p
8717	TRADD	repression	protein-coding	Yes			0	1.00E+00	
130026	ICA1L	repression	protein-coding				3	4.53E-02	hsa-miR-33a-5p|hsa-miR-24-3p|hsa-miR-7-5p
9697	TRAM2	repression	protein-coding				3	4.74E-01	hsa-miR-124-3p|hsa-miR-7-5p|hsa-miR-25-3p
27095	TRAPPC3	repression	protein-coding				0	1.00E+00	
10155	TRIM28	activation	protein-coding	Yes			0	1.00E+00	
10475	TRIM38	repression	protein-coding	Yes			0	1.00E+00	
57468	SLC12A5	repression	protein-coding				5	4.53E-02	hsa-miR-33a-5p|hsa-miR-181d-5p|hsa-miR-25-3p|hsa-miR-181a-5p|hsa-miR-181b-5p
11078	TRIOBP	repression	protein-coding				2	2.66E-01	hsa-miR-24-3p|hsa-miR-93-5p
54802	TRIT1	repression	protein-coding				1	4.68E-01	hsa-miR-24-3p
55621	TRMT1	activation	protein-coding				0	1.00E+00	
7226	TRPM2	repression	protein-coding				0	1.00E+00	
25987	TSKU	activation	protein-coding				2	2.16E-01	hsa-miR-93-5p|hsa-miR-124-3p
7106	TSPAN4	repression	protein-coding				0	1.00E+00	
22996	TTC39A	repression	protein-coding				2	4.53E-02	hsa-miR-27a-3p|hsa-miR-27b-3p
7272	TTK	activation	protein-coding		Yes		0	1.00E+00	
23170	TTLL12	activation	protein-coding				0	1.00E+00	
7291	TWIST1	activation	protein-coding	Yes			1	6.28E-01	hsa-miR-33a-5p
200081	TXLNA	repression	protein-coding				2	9.13E-01	hsa-miR-124-3p|hsa-miR-93-5p
116068	LYSMD3	repression	protein-coding				6	4.54E-02	hsa-miR-33a-5p|hsa-miR-124-3p|hsa-miR-93-5p|hsa-miR-320a|hsa-miR-27b-3p|hsa-miR-27a-3p
10628	TXNIP	repression	protein-coding				4	7.26E-02	hsa-miR-93-5p|hsa-miR-320a|hsa-miR-27b-3p|hsa-miR-27a-3p
10907	TXNL4A	activation	protein-coding				0	1.00E+00	
7298	TYMS	activation	protein-coding				0	1.00E+00	
11338	U2AF2	activation	protein-coding				0	1.00E+00	
124402	UBALD1	activation	protein-coding				0	1.00E+00	
55833	UBAP2	activation	protein-coding				0	1.00E+00	

11065	UBE2C	activation	protein-coding				0	1.00E+00	
7328	UBE2H	repression	protein-coding				0	1.00E+00	
7329	UBE2I	activation	protein-coding	Yes			0	1.00E+00	
118424	UBE2J2	repression	protein-coding				0	1.00E+00	
27338	UBE2S	activation	protein-coding				0	1.00E+00	
23499	MACF1	repression	protein-coding				4	4.54E-02	hsa-miR-33a-5p|hsa-miR-296-5p|hsa-miR-93-5p|hsa-miR-124-3p
23352	UBR4	repression	protein-coding				0	1.00E+00	
84333	PCGF5	repression	protein-coding				6	4.54E-02	hsa-miR-33a-5p|hsa-miR-124-3p|hsa-miR-24-3p|hsa-miR-320a|hsa-miR-25-3p|hsa-miR-7-5p
201294	UNC13D	repression	protein-coding				0	1.00E+00	
7378	UPP1	repression	protein-coding				0	1.00E+00	
84300	UQCC2	activation	protein-coding				0	1.00E+00	
7386	UQCRFS1	activation	protein-coding				0	1.00E+00	
9816	URB2	activation	protein-coding				1	4.55E-01	hsa-miR-33a-5p
27340	UTP20	activation	protein-coding				0	1.00E+00	
7402	UTRN	repression	protein-coding				2	8.78E-01	hsa-miR-7-5p|hsa-miR-93-5p
7407	VARS	activation	protein-coding				0	1.00E+00	
10493	VAT1	repression	protein-coding				1	1.00E+00	hsa-miR-124-3p
57687	VAT1L	repression	protein-coding				3	1.25E-01	hsa-miR-24-3p|hsa-miR-27b-3p|hsa-miR-27a-3p
25806	VAX2	activation	protein-coding	Yes			0	1.00E+00	
9525	VPS4B	repression	protein-coding				3	1.33E-01	hsa-miR-25-3p|hsa-miR-124-3p|hsa-miR-320a
55759	WDR12	activation	protein-coding				0	1.00E+00	
57418	WDR18	activation	protein-coding				0	1.00E+00	
604	BCL6	repression	protein-coding	Yes			5	4.71E-02	hsa-miR-181d-5p|hsa-miR-124-3p|hsa-miR-93-5p|hsa-miR-181a-5p|hsa-miR-181b-5p
23160	WDR43	activation	protein-coding				3	5.90E-02	hsa-miR-345-5p|hsa-miR-27a-3p|hsa-miR-27b-3p
144406	WDR66	repression	protein-coding				0	1.00E+00	
79819	WDR78	repression	protein-coding				0	1.00E+00	
29062	WDR91	repression	protein-coding				0	1.00E+00	
7456	WIPF1	repression	protein-coding				4	2.52E-01	hsa-miR-124-3p|hsa-miR-24-3p|hsa-miR-320a|hsa-miR-7-5p
116496	FAM129A	repression	protein-coding				4	4.71E-02	hsa-miR-25-3p|hsa-miR-33a-5p|hsa-miR-93-5p|hsa-miR-124-3p
55841	WWC3	repression	protein-coding				1	1.00E+00	hsa-miR-124-3p
3659	IRF1	repression	protein-coding	Yes			4	4.71E-02	hsa-miR-24-3p|hsa-miR-93-5p|hsa-miR-124-3p|hsa-miR-345-5p
7516	XRCC2	activation	protein-coding				0	1.00E+00	

10413	YAP1	repression	protein-coding	Yes			5	8.82E-02	hsa-miR-33a-5p|hsa-miR-124-3p|hsa-miR-7-5p|hsa-miR-27b-3p|hsa-miR-27a-3p
51067	YARS2	activation	protein-coding				0	1.00E+00	
150223	YDJC	activation	protein-coding				0	1.00E+00	
54502	RBM47	repression	protein-coding				5	4.71E-02	hsa-miR-181d-5p|hsa-miR-124-3p|hsa-miR-25-3p|hsa-miR-181a-5p|hsa-miR-181b-5p
64766	S100PBP	repression	protein-coding				6	4.84E-02	hsa-miR-181d-5p|hsa-miR-124-3p|hsa-miR-7-5p|hsa-miR-93-5p|hsa-miR-181a-5p|hsa-miR-181b-5p
7704	ZBTB16	repression	protein-coding	Yes			0	1.00E+00	
49854	ZBTB21	activation	protein-coding				4	1.31E-01	hsa-miR-124-3p|hsa-miR-93-5p|hsa-miR-181b-5p|hsa-miR-345-5p
5412	UBL3	repression	protein-coding				5	4.84E-02	hsa-miR-181d-5p|hsa-miR-124-3p|hsa-miR-330-3p|hsa-miR-181b-5p|hsa-miR-181a-5p
140685	ZBTB46	repression	protein-coding				0	1.00E+00	
3104	ZBTB48	repression	protein-coding	Yes			0	1.00E+00	
339487	ZBTB8OS	repression	protein-coding				0	1.00E+00	
84524	ZC3H8	activation	protein-coding	Yes			0	1.00E+00	
219654	ZCCHC24	repression	protein-coding				5	1.07E-01	hsa-miR-124-3p|hsa-miR-24-3p|hsa-miR-27b-3p|hsa-miR-330-3p|hsa-miR-27a-3p
441951	ZFAS1	activation	ncRNA				0	1.00E+00	
7538	ZFP36	repression	protein-coding				3	7.30E-02	hsa-miR-7-5p|hsa-miR-27a-3p|hsa-miR-27b-3p
84619	ZGPAT	repression	protein-coding	Yes			0	1.00E+00	
284307	ZIK1	activation	protein-coding				0	1.00E+00	
9204	ZMYM6	repression	protein-coding				0	1.00E+00	
51222	ZNF219	activation	protein-coding	Yes			2	7.19E-02	hsa-miR-124-3p|hsa-miR-296-5p
8187	ZNF239	activation	protein-coding	Yes			0	1.00E+00	
55659	ZNF416	activation	protein-coding				0	1.00E+00	
7594	ZNF43	activation	protein-coding	Yes			0	1.00E+00	
80818	ZNF436	repression	protein-coding	Yes			4	8.03E-02	hsa-miR-24-3p|hsa-miR-181b-5p|hsa-miR-320a|hsa-miR-7-5p
197407	ZNF48	activation	protein-coding				0	1.00E+00	
147929	ZNF565	activation	protein-coding	Yes			0	1.00E+00	
51545	ZNF581	activation	protein-coding				0	1.00E+00	
7625	ZNF74	activation	protein-coding	Yes			0	1.00E+00	
7554	ZNF8	activation	protein-coding				0	1.00E+00	
57169	ZNFX1	repression	protein-coding				1	1.00E+00	hsa-miR-93-5p
9753	ZSCAN12	activation	protein-coding	Yes			2	1.48E-01	hsa-miR-25-3p|hsa-miR-7-5p

Supplementary Table S4: Primers listed in 5′ to 3′ for ChIP-qPCR

Peak	Forward	Reverse
peak_1427	CAGAGACCTCACTTCCTCGC	CAGGCAAGAGCACAACATGG
peak_3070	CTTGATACTAAGTAGCCCTCCCC	TCCCGTTTTTCTCAGATCTTCAGT
peak_3071	ATTTCAAAGGGCTTCGGGGT	GTCGCCACGAATCTGAGAGG
peak_4674	TCTGACCCAGCCTCTGCTTA	GCTGTCCGGGTGTGAAGTAA
peak_4675	GGGCAGAACAGATTCAGGCA	ACCGGTATAAAGCGGTAGGC
peak_4676	CGGCTGGGAGCTTTAGGAG	ACCCGCTTAGCTCCATTTCC
peak_4677	CACACGTGAGGTGCGTAATG	CTGGGATAGCTTCCTCCCCT
peak_4978	GGCCGGAGAAAACACGAGTA	ATGGCTGTTGCTTGGCAATG
peak_9403	GGTAACCTGTCTGCAGTGGG	AAGGTGGGGCCTCCTGG
peak_9404	GTGGGCACATTGAGTTGCAC	GAACCTGTCTCTGCGGTCTG
peak_17259	AGTTGGTCTTTTTGGAATGAGCC	AGCAAAGTTCCAGACAGAGGT
peak_39619	GAGATCCGTGGGCTCCTTTG	GATCTAGAGCTTTCCAGGCCC
peak_43652	CCCTCTCTCACCCCCGAG	AACTTTCCAACAGCCCGAGG
peak_44366	GAAGTGAGGCTAGCGGAGC	AGCAAAGCAACTGAAAGCGG
peak_44367	GGAGACATCGACGGTGAGTG	CGCCGTAACCCCTTCCTC
peak_66706	GGAGACACAGTGAGTGAGTGG	TGTCCCTTTCACGCTTCTCC
peak_66707	CTGTTCCCTTCCATTGGGGT	TGAGCCAAGTAACAAAGTCCAAC
peak_66488	CGAACCCAAGGTGCTTTGTC	ATCCACTGCCTTTGGTTGGG
